# Designing a Sulfur Vacancy Redox Disruptor for Photothermoelectric and Cascade-Catalytic-Driven Cuproptosis–Ferroptosis–Apoptosis Therapy

**DOI:** 10.1007/s40820-025-01828-8

**Published:** 2025-07-04

**Authors:** Mengshu Xu, Jingwei Liu, Lili Feng, Jiahe Hu, Wei Guo, Huiming Lin, Bin Liu, Yanlin Zhu, Shuyao Li, Elyor Berdimurodov, Avez Sharipov, Piaoping Yang

**Affiliations:** 1 Key Laboratory of Superlight Materials and Surface Technology, Ministry of Education, College of Materials Science and Chemical Engineering, Harbin Engineering University, Harbin, 150001 People’s Republic of China; 2 Key Laboratory of Photochemical Biomaterials and Energy Storage Materials, Heilongjiang Province and College of Chemistry and Chemical Engineering, Harbin Normal University, Harbin, 150025 People’s Republic of China; 3 Cancer Center, Department of Neurosurgery, Zhejiang Provincial People’s Hospital, Affiliated People’s Hospital, Zhejiang, 310014 People’s Republic of China; 4 Department of Physical Chemistry, National University of Uzbekistan, 100034 Tashkent, Uzbekistan; 5 Faculty of Pharmacy, Department of Inorganic, Physical and Colloidal Chemistry, Tashkent Pharmaceutical Institute, 100015 Tashkent, Uzbekistan

**Keywords:** Cuproptosis, Enzyme catalysis, Immunology, Photothermoelectric catalysis, Vacancy defects

## Abstract

The therapeutic efficacy of cuproptosis, ferroptosis, and apoptosis is hindered by inadequate intracellular copper and iron levels, hypoxia, and elevated glutathione (GSH) expression in tumor cells. Thermoelectric technology is an emerging frontier in medical therapy that aims to achieve efficient thermal and electrical transport characteristics within a narrow thermal range for biological systems. Here, we systematically constructed biodegradable Cu_2_MnS_3-x_-PEG/glucose oxidase (MCPG) with sulfur vacancies (S_V_) using photothermoelectric catalysis (PTEC), photothermal-enhanced enzyme catalysis, and starvation therapy. This triggers GSH consumption and disrupts intracellular redox homeostasis, leading to immunogenic cell death. Under 1064 nm laser irradiation, MCPG enriched with S_V_, owing to doping, generates a local temperature gradient that activates PTEC and produces toxic reactive oxygen species (ROS). Hydroxyl radicals and oxygen are generated through peroxide and catalase-like processes. Increased oxygen levels alleviate tumor hypoxia, whereas hydrogen peroxide production from glycometabolism provides sufficient ROS for a cascade catalytic reaction, establishing a self-reinforcing positive mechanism. Density functional theory calculations demonstrated that vacancy defects effectively enhanced enzyme catalytic activity. Multimodal imaging-guided synergistic therapy not only damages tumor cells, but also elicits an antitumor immune response to inhibit tumor metastasis. This study offers novel insights into the cuproptosis/ferroptosis/apoptosis pathways of Cu-based PTEC nanozymes.

**Supplementary Information:**

The online version contains supplementary material available at 10.1007/s40820-025-01828-8.

## Highlights

- The glycometabolism and enzyme activity of Cu_2_MnS_3-x_-PEG/glucose oxidase (MCPG) achieve a cascade catalytic reaction, continuously replenishing the deficient H_2_O_2_ and O_2_ and inducing adequate reactive oxygen species supply.
- Density functional theory calculations indicate that Mn doping facilitates the structural reconstruction and evolution of sulfur vacancies (S_V_) sites, leading to a remarkable increase in the catalytic activity of Cu_2_MnS_3-x_.
- Under 1064 nm laser irradiation, MCPG with abundant S_V_ drives charge carrier diffusion from hotter to cooler regions, thus creating a potential difference and activating the photothermoelectric catalysis.

**Supplementary Information:**

The online version contains supplementary material available at 10.1007/s40820-025-01828-8.

## Graphical abstract

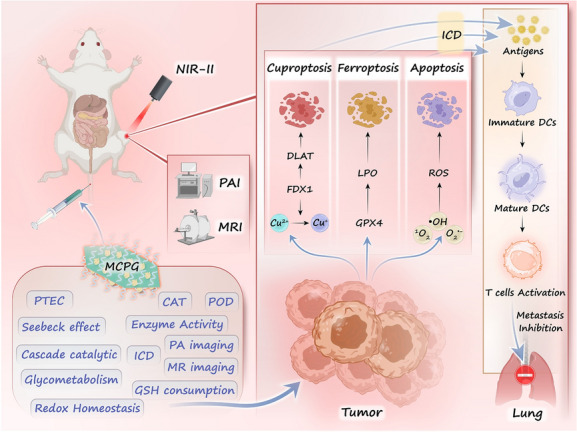

## Introduction

Cancer is a considerable challenge that necessitates the development of innovative therapeutic strategies. Thermoelectric catalytic therapy (TECT) is an emerging therapeutic modality that converts thermal energy into electrical electricity via a temperature gradient, separating electron–hole (e^−^–h^+^) pairs and generating an intrinsic electric field [1, 2]. This process triggers redox reactions and reactive oxygen species (ROS) production, subsequently triggering tumor cell apoptosis [3]. However, TECT efficacy is constrained by the limited temperature variation within the organism. Driven by the photothermoelectric (PTE) effect, using heat as an energy input demonstrates enhanced efficiency in light–thermal–electric energy conversion. Notably, PTE catalysis (PTEC) is an emerging intelligent strategy. When a PTE material is exposed to laser, a regional temperature elevation occurs because of its photothermal performance. This subsequently creates a temperature gradient, leading to the movement of charge carriers from the hotter to cooler regions and establishing a potential difference, which is known as the Seebeck effect [4–7]. In particular, TECT and PTEC exhibit distinct differences in mechanisms and energy source. TECT uses thermal energy and electrical energy as the primary driving forces, induces localized thermal effects via temperature gradients, and integrates the electric field regulation to catalyze reactions. Specifically, thermal energy facilitates the adsorption and dissociation processes on the catalyst surface, whereas electrical energy enhances the efficiency of redox reactions. In contrast, PTEC integrates light energy, thermal energy, and electrical energy into a unified system. Light energy serves as the excitation source and generates e^−^–h^+^ pairs via photocatalysts to participate in catalytic reactions. Thermal energy and electrical energy act as auxiliary means to further enhance the reaction rate and regulate carrier separation efficiency, respectively. Despite the examination of the PTEC in several materials, including Bi_0.5_Sb_1.5_Te_3_/Bi_2_Te_2.8_Se_0.2_ heterojunctions, Bi_0.5_Sb_1.5_Te_3_/CaO_2_ nanosheets, and Ag-Ag_2_S nanoparticles, these materials typically possess intricate structures and compositions, posing notable challenges in terms of synthesis, safety, and biocompatibility [8, 9]. Furthermore, owing to the low photothermal conversion efficiency, a limited temperature difference persists even with complex materials. Consequently, it is essential to prioritize the design of simple compositions for the PTEC processes.

Economical copper chalcogenides have emerged as promising candidates for thermoelectric applications owing to their favorable band gaps and phonon-liquid characteristics. However, conventional CuS has a limited power factor (PF), which limits its thermoelectric performance. Doping has emerged as a promising solution for this challenge [10]. Defect engineering is used to modify charge density and reduce energy potential [11]. Doping further optimizes the structure and introduces additional carriers to enhance the local electric field, thereby increasing the electrical conductivity [12, 13]. Moreover, Mn serves as an essential nutrient in numerous physiological processes and demonstrates distinct biodegradability and biosafety. Mn doping introduces a significant number of holes owing to the disparities in the outer electrons, enhancing the conductivity and modifying the internal electron state of the active materials. Thus, Cu_2_MnS_3-x_ can achieve exceptional degradation and PTEC performance. Nonetheless, despite the extensive application of CuS in photothermal therapy (PTT), the therapeutic mechanisms of Cu_2_MnS_3-x_ in PTEC remain unclear [14, 15].

Cuproptosis is an exceptional process of cell death caused by Cu-dependent mitochondrial dysfunction [16–18]. It selectively affects mitochondrial respiratory cells by disrupting tricarboxylic acid (TCA) cycle-related metabolites [19–22]. Based on Tsvetkov et al*.*, ferredoxin 1 (FDX1) and lipoyl synthase (LIAS) function as upstream regulators of protein lipidization, promoting the lipoylation of TCA cycle enzyme, particularly dihydrolipoamide S-acetyltransferase (DLAT) [23–25]. Conventionally, under elevated intracellular copper levels, FDX1 facilitates Cu^2+^ reduction to toxic Cu^+^, which directly binds to DLAT and disrupts Fe–S cluster proteins, thereby inducing cuproptosis [26]. The repression of DLAT leads to the downregulation of FDX1 and LIAS expression, which impairs the TCA cycle and causes cell death [27–29]. Unfortunately, current research on cuproptosis remains in its infancy, and several critical challenges necessitate urgent attention. First, the endogenous copper-chelating agent, glutathione (GSH), significantly impedes the occurrence of cuproptosis [30]. Second, the clinical applications of commonly used Cu carriers (such as elesclomol and disulfiram) are limited [31, 32]. Third, hypoxia and the presence of glucose diminish the responsiveness of tumor cells [33, 34]. Therefore, it is essential to design a copper carrier capable of depleting endogenous GSH, consuming glucose, and facilitating mitochondrial respiration through oxygen provision to induce cuproptosis.

**Scheme 1:**
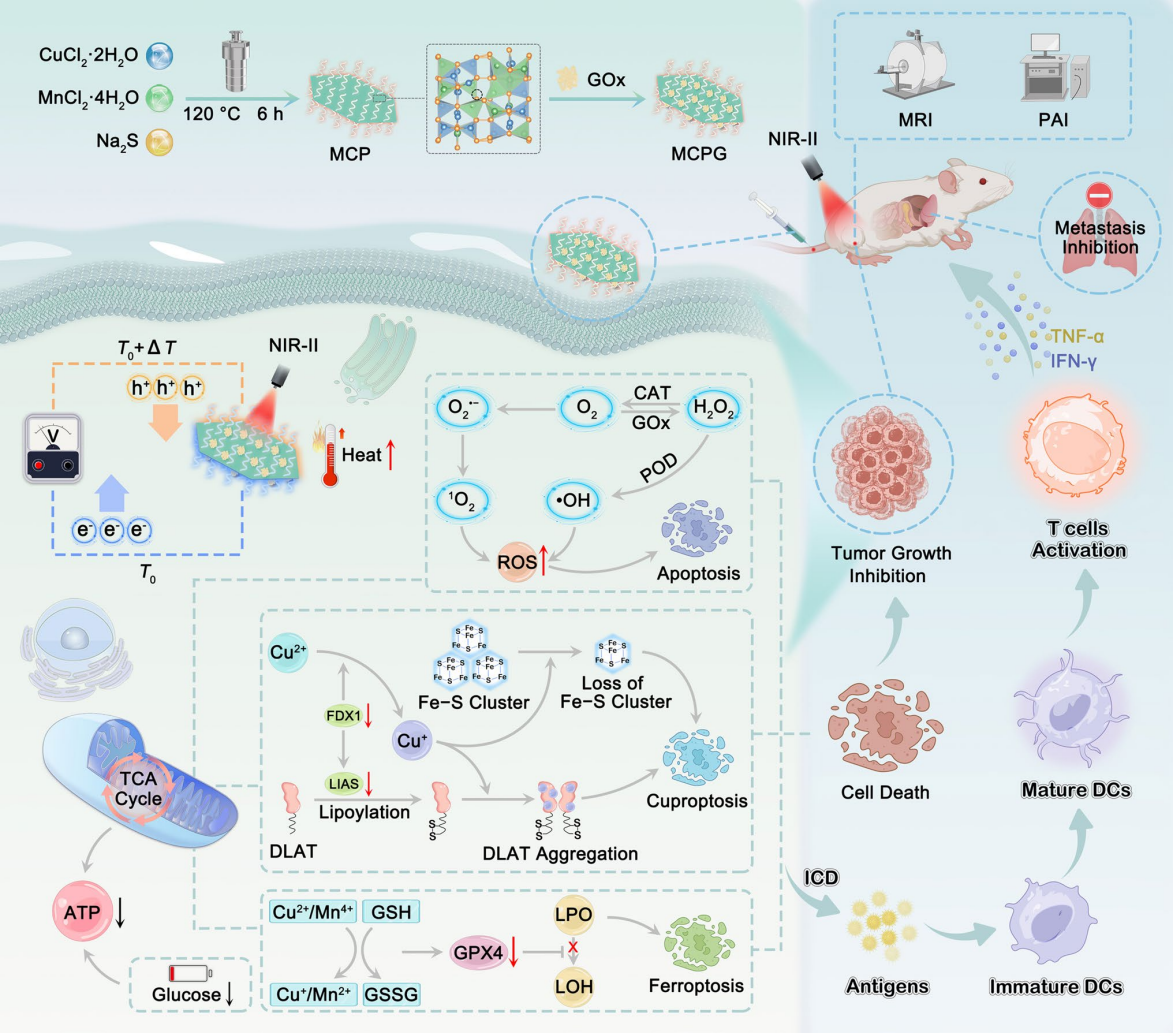
Scheme of MCPG synthesis process as well as synergistic antitumor mechanism of PTEC, photothermal-enhanced enzyme activity, and starvation therapy. The integration of defective MCP and GOx establishes a cascade catalytic mechanism to continuously replenish insufficient H_2_O_2_ and O_2_. Cuproptosis/ferroptosis/apoptosis are activated by disrupting tumor redox homeostasis. MCPG stimulates the immune system, induces ICD, and elicits a robust immune response

Herein, we utilize an innovative approach to synthesize two-dimensional ultrathin Cu_2_MnS_3-x_-PEG (MCP) nanosheets with vacancy defects, followed by the covalent immobilization of glucose oxidase (GOx) on their surfaces. In contrast to the traditional flower-like structure, the small ultrathin nanosheets displayed enhanced cellular uptake capability. The achieved Cu_2_MnS_3-x_-PEG/GOx (MCPG) nanosheets exhibited remarkable efficacy in inducing cuproptosis/ferroptosis/apoptosis and eliciting an antitumor immune response via oxidative stress triggered by PTEC, photothermal-enhanced enzyme catalysis, and starvation therapy (Scheme 1). The designed nanosheets catalyzed hydrogen peroxide (H_2_O_2_) decomposition and depleted overexpressed GSH, thereby interfering with intracellular redox homeostasis. By leveraging the elevated glucose consumption in tumor regions relative to normal tissues, GOx can effectively catalyze the conversion of excess glucose into gluconic acid and H_2_O_2_ [35–37]. Due to the highly reversible Cu^+^/Cu^2+^ redox pair and sulfur vacancy (S_V_)-induced peroxidase (POD)-like and catalase (CAT)-like enzyme activities, increased H_2_O_2_ further facilitates hydroxyl radicals (·OH) and O_2_ production. The release of O_2_ can ameliorate tumor hypoxia and serve as a reactant for starvation therapy, thereby promoting H_2_O_2_ generation, achieving cascade catalytic reactions and establishing a positive feedback loop. Density functional theory (DFT) calculations revealed that doping-engineered vacancy defects markedly enhanced enzyme catalytic activity. There is compelling evidence that PTT can expedite the catalytic process in favor of the enzyme catalytic therapeutic effect [38–41]. Therefore, integrating MCP with GOx triggers a self-reinforced ROS storm and intensifies oxidative stress. This approach not only damages tumor cells, but also induces immunogenic cell death (ICD), thereby eliciting an immune response and inhibiting tumor metastasis.

## Experimental Section

### Synthesis of MCP

CuCl_2_·2H_2_O (0.1705 g), MnCl_2_·4H_2_O (0.0989 g), and mPEG–COOH (0.1 g) were dissolved in ethylene glycol (20 mL). Next, an additional Na_2_S ethylene glycol solution (20 mL) was slowly introduced into the aforementioned mixture under vigorous stirring. After stirring for 2 h, the mixture was transferred into a stainless steel autoclave and heated at 120 °C for 6 h. The dark green precipitate was collected by centrifugation.

### Preparation of MCPG

GOx (2 mg), carbodiimide hydrochloride (EDC·HCl) (10 mg), and N-Hydroxysuccinimide (NHS) (5 mg) were dissolved in deionized water (10 mL) and stirred in the dark for 2 h. Thereafter, the prepared MCP (4 mL, 1 mg mL^−1^) was then added into the solution and stirred for 12 h in the dark. Eventually, the MCPG nanosheets were obtained by centrifugation.

### Photothermal Performance of MCPG

MCPG solutions with various concentrations were prepared and irradiated upon 1064 nm laser (0.7 W cm^−2^) for 600 s. Subsequently, the changes in temperature were recorded using a thermal imaging device at different time intervals. Additionally, the photothermal conversion efficiency (*η*) of MCPG was calculated based on the cooling period curve after irradiation of the solution, which was obtained using the following equation:

1 $$\eta_{T} = \frac{{hS\left( {T_{\max } - T_{{{\text{surr}}}} } \right) - Q_{{{\text{dis}}}} }}{{I(1 - 10^{{ - A_{\lambda } }} )}}$$

where *I* represents the laser power, *A*_*λ*_ reveals the absorbance value of MCPG at the excitation wavelength (*λ* = 1064 nm), *h* is the heat transfer coefficient, *S* is the superficial area, *T*_max_ and *T*_surr_ signify the highest temperature of MCPG after irradiation and ambient temperature, *Q*_dis_ = (5.4 × 10^−4^) *I J* s^−1^, and *η* demonstrates the photothermal conversion efficiency.

### Evaluation of ·OH Generation and Enzymatic Catalytic Activity of MCPG

The catalytic ability of the MCPG nanosheets was analyzed at 25 and 50 °C in the presence of H_2_O_2_ via 3,3′,5,5′-tetramethylbiphenylmethane (TMB), o-phenylenediamine (OPD), and 2,2′-amino-di(2-ethyl-benzothiazoline sulphonic acid-6) ammonium salt (ABTS), and the absorbance was measured. Changes in the absorbance of TMB at 652 nm were observed after treatment with various concentrations of H_2_O_2_ (6, 20, 40, 60, and 100 mM). The initial reaction rate (*v*_0_) to generate the ·OH value for each H_2_O_2_ concentration was calculated using the Beer–Lambert law (Eq. 2) (*ε* = 3.9 × 10^4^ M^−1^ cm^−1^). Subsequently, the Michaelis–Menten constant was calculated using the Michaelis–Menten equation. (Eq. 3). The Michaelis–Menten constant (*K*_m_) and maximum reaction velocity (*V*_max_) were obtained from Lineweaver–Burk plots (Eq. 4):

2 $$A = \varepsilon \,\cdot\,l\,\cdot\,c$$

3 $$v_{0} = \frac{{V_{\max } \cdot\left[ S \right]}}{{K_{m} + \left[ S \right]}}$$

4 $$\frac{1}{{v_{0} }} = \frac{{K_{m} }}{{V_{\max } }}\cdot\frac{1}{\left[ S \right]} + \frac{1}{{V_{\max } }}$$

To assess the nanozyme activity, different amounts of MCPG (0.004, 0.008, 0.016, 0.03, and 0.06 mg) were cultivated with H_2_O_2_ and TMB. The absorbance at 652 nm was measured every 10 s, and the nanozyme activity (units) was calculated using the following equation:

5 $$b_{{{\text{nanozyme}}}} = V/(\varepsilon \times l) \times \Delta A/\Delta t$$

where *l* represents the diameter of the cuvette, *ε* represents the molar absorption coefficient, *V* refers to the total volume of reaction solution, and Δ*A*/Δ*t* signifies the initial rate of the absorbance variation.

### DFT Calculation

In this study, DFT calculations were performed using a projector augmented-wave technique within the Vienna ab initio simulation package (VASP). The plane-wave basis sets were employed with an energy cutoff of 450 eV. The exchange–correlation potential was processed by adopting a generalized gradient approximation (GGA) with Perdew–Burke–Ernzerhof (PBE) parametrization. Brillouin zone integration was conducted on a 2 × 2 × 1 Г-centered Monkhorst–Pack grid via VASPKIT. The structure was completely relaxed until the maximum force applied to any atom was below 0.03 eV Å^−1^, and the energy convergence criterion was set to 10^−5^ eV. Additionally, Grimme’s DFT-D3 model was utilized to incorporate van der Waals corrections. To prevent the periodic interaction between interface structures, a vacuum layer of 15 Å was introduced along the c-axis perpendicular to the interface.

### GSH Depletion Evaluation

The GSH depletion effect of MCPG was investigated by using 5,5′-dithiobis-(2-nitrobenzoic acid) (DTNB) as a chemical probe. MCPG (200 μg mL^−1^) was homogeneously mixed with GSH (10 mM) in PBS (pH = 7.4) for various time intervals (0, 10, 20, 60, 120, 180, and 360 min). GSH was detected by adding DTNB (0.2 mM), and the change in absorbance at 412 nm was determined.

### Evaluation of Superoxide Radicals (O_2_^•−^) Generation

We examined O_2_^•−^ production using the Nitro-blue tetrazolium chloride (NBT) assay. The NBT solution (30 μg mL^−1^) in dimethyl sulfoxide (DMSO) was mixed with MCPG (200 μg mL^−1^). After irradiation with a 1064 nm laser, the solution was naturally cooled down (laser off). Five laser irradiation/cooling cycles were conducted, and the absorbance at 528 nm was recorded.

### Assessment of Singlet Oxygen (^1^O_2_) Production

MCPG (200 μg mL^−1^) was added to the 1,3-diphenylisobenzofuran (DPBF) solution (30 μg mL^−1^). After various laser irradiation/cooling cycles, the absorbance of the DPBF at 420 nm was determined. To demonstrate the thermoelectric effect, the laser irradiation was replaced by a hot water bath maintained at 55 °C, whereas a 15 °C ice water bath was used as a cold source to establish a temperature gradient. The ultraviolet–visible (UV–vis) spectra of DPBF were recorded during five heating/cooling cycles.

### In Vitro Cell Viability Evaluation

For biocompatibility, L929 cells were pre-inoculated in a 96-well plate and incubated with different concentrations of MCPG for 12 and 24 h, respectively. Methyl thiazolyl tetrazolium (MTT) (20 μL, 5 mg mL^−1^) was added into each well. After cultivation of 4 h, DMSO (150 μL) was added. Subsequently, the absorbance at 490 nm was monitored, and cell viability was calculated. To determine the cytotoxicity, 4T1 cells were treated with (1) control, (2) NIR-II laser, (3) MCP, (4) MCPG, (5) MCP + NIR-II, and (6) MCPG + NIR-II. After cultivation for 12 h, MTT (20 μL) was added and continued culture for 4 h. DMSO (150 μL) was added immediately, and the cell viability was calculated.

### Intracellular ROS Detection

4T1 cells were treated with the following conditions: (1) control, (2) MCP, (3) MCPG, (4) NIR-II laser, (5) MCP + NIR-II, and (6) MCPG + NIR-II. After cultivation for 4 h, the laser irradiated groups were exposed to a 1064 nm laser. Moreover, 2,7-dichlorofluorescein diacetate (DCFH-DA) (10 μM) was added, and the cells were cultivated for 30 min. Eventually, all fluorescence images were acquired using the fluorescence microscope.

### Intracellular Adenosine-5′-triphosphate (ATP) Detection

4T1 cells were cultivated overnight and incubated with the following treatments: (1) control, (2) MCP, (3) MCPG, (4) NIR-II laser, (5) MCP + NIR-II, and (6) MCPG + NIR-II. Furthermore, the cells were collected and lysed to determine the intracellular ATP levels.

### Lactate Dehydrogenase (LDH) Detection

4T1 cells were cultivated overnight and cultured with the following formulations: (1) control, (2) MCP, (3) MCPG, (4) NIR-II laser, (5) MCP + NIR-II, and (6) MCPG + NIR-II. The release of LDH was measured using an LDH cytotoxicity assay kit. In particular, the data for each treatment group were displayed as a percentage of the control group.

### Intracellular GSH Depletion

4T1 cells were incubated with various concentrations of MCPG for 24 h, with PBS as a control. Intracellular GSH levels were measured according to the instructions of the manufacturer. Therefore, the relative GSH levels were derived for each treatment group based on the control group.

### Living/Dead Cells Staining

4T1 cells were treated with (1) control, (2) MCP, (3) MCPG, (4) NIR-II laser, (5) MCP + NIR-II, and (6) MCPG + NIR-II, respectively. After 4 h of treatment, the 4T1 cells were stained with Calcein-AM (2 μM) and PI (4 μM) for 30 min. Finally, the cells were imaged using a fluorescence microscope.

### Intracellular Lipid Peroxide Detection

4T1 cells were cultured with the following formulations: (1) control, (2) MCP, (3) MCPG, (4) NIR-II laser, (5) MCP + NIR-II, and (6) MCPG + NIR-II. After 4 h of incubation, the 4T1 cells were stained with C11-BODIPY581/591 (10 μM) and incubated for 20 min. Finally, the fluorescence microscope was applied to detect the fluorescence images.

### Evaluation of DLAT Aggregation

4T1 cells were seeded and cultured overnight in a 6-well plate. The cells were subsequently treated with (1) control, (2) MCP, (3) MCPG, (4) NIR-II laser, (5) MCP + NIR-II, and (6) MCPG + NIR-II for 12 h. Furthermore, the cells were fixed with 4% paraformaldehyde and treated with the DLAT antibody overnight at 4 °C. Cells were then cultivated with the secondary antibody for 1 h and imaged using a fluorescence microscope.

### Western Blot Assays

4T1 cells were inoculated in a 6-well culture plate and treated with (1) control, (2) NIR-II laser, (3) MCP, (4) MCPG, (5) MCP + NIR-II, and (6) MCPG + NIR-II. 4T1 cells were treated with cold radioimmunoprecipitation assay (RIPA) lysis buffer (Easen, 20115ES60). The protein concentration in the cell lysates was determined using bicinchoninic acid (BCA) assay kit (Wanleibio, 15122020). Subsequently, proteins were isolated through sodium dodecyl sulfate–polyacrylamide gel electrophoresis (SDS-PAGE) and transferred to polyvinylidene fluoride membranes (Easen, 36126ES03). Rapid blocking western (Easen, 36122ES76) was used to block the membranes, followed by incubation with the primary antibodies DLAT (HUABIO, JE34-38), FDX1 (HUABIO, Cat# JE63-56), and LIAS (HUABIO, Cat# PSH04-04) overnight at 4 °C. After rinsing with TBST, the membranes were incubated for 1 h with a secondary antibody and rinsed again with TBST. Eventually, the protein signals were visualized using a Bio-Rad imaging systems.

### In Vitro ICD Effect

4T1 cells were treated with (1) control, (2) MCP, (3) MCPG, (4) NIR-II laser, (5) MCP + NIR-II, and (6) MCPG + NIR-II. After incubation for 30 min, the cells were fixed. Subsequently, the cells were incubated with 0.3% Triton X-100 for 30 min. Cells were incubated with anti-high-mobility group box 1 (HMGB1) for 30 min, followed by incubation with a secondary antibody. After DAPI treatment, the cells were rinsed and fluorescence images were recorded.

### Apoptosis Detection Assay

4T1 cells were randomly inoculated in a 6-well plate overnight, and the medium was replaced. The cells were divided into 6 groups and treated with (1) control, (2) MCP, (3) MCPG, (4) NIR-II laser, (5) MCP + NIR-II, and (6) MCPG + NIR-II. Cells from all groups were trypsinized and rinsed, followed by the addition of Annexin V-fluorescein isothiocyanate (FITC)/PI apoptosis detection reagent. Ultimately, the apoptosis rate of all treatment groups was quantified using a BD Accuri C6 flow cytometer.

### In Vivo PTT Performance

Animal experiments were approved by the Ethics Committee of the Second Affiliated Hospital of Harbin Medical University (No. SYDW 2019–82). Animal experiments were conducted in strict adherence to the Guidelines for the Care and Use of Laboratory Animals of the Drug Safety Evaluation Center of the Harbin Medical University. The mice were intravenously injected with MCPG (10 mg kg^−1^) for 12 h, and the tumor was irradiated upon 1064 nm laser. The temperature changes at different time intervals were monitored using an infrared camera.

### Photoacoustic (PA) and Magnetic Resonance (MR) Imaging Performance of MCPG

In vitro PA signals of MCPG dispersed in PBS at different concentrations were examined using Visual Sonics. In vivo PA signals were explored in mice after anesthesia and intravenous administration of MCPG solution (100 μL). For the In vitro *T*_1_-weighted MR images, MCPG with various Cu concentrations was dissolved in PBS and *r*_1_ relaxation was obtained by curve fitting with 1/*T*_1_ relaxation time (s^−1^). MCPG (100 μL) was injected into the mice, and imaging was performed at various times.

### In Vivo Biodistribution of MCPG

After the mice were intravenously injected with MCPG at varying time points, tumors and major organs were collected from the euthanized mice. Then, the biodistribution of Cu was performed by ICP–OES analysis.

### Hemolysis Assay

Red blood cells (RBCs) separated from mice blood were washed with PBS. The supernatant was diluted tenfold with PBS until it became clear. Subsequently, PBS, water, and MCPG at varying concentrations (0.9 mL) were added to diluted blood (0.1 mL). The mixture was centrifuged after cultivation at 37 °C for 1 h, and hemolysis was calculated using the following formula:

6 $${\text{Hemolysis\% = }}\frac{{A_{{{\text{sample}}}} - A_{{{\text{control(}} - {)}}} }}{{A_{{\text{control(+)}}} - A_{{{\text{control(}} - {)}}} }}$$

where* A* is the absorption rate of the supernatant.

### In Vivo Therapeutic Performance of MCPG

Four-week-old female BALB/c mice were selected for this assay. First, 4T1 cells (100 μL, 2 × 10^6^ cells) were subcutaneously inoculated into the right side of mice to construct the tumor model. The mice were then separated into 6 groups: (1) control, (2) MCP, (3) MCPG, (4) NIR-II laser, (5) MCP + NIR-II, (6) MCPG + NIR-II. Each group of mice was injected intravenously with different samples (100 μL). Then, the tumor size was then measured to assess the treatment effect. The tumor volumes were derived using the following equation:

7 $$V = l \times w^{2} /2$$

where *l*, *V* and *w* represent the length, volume, and width of the tumor, respectively.

### In Vivo Dendritic Cells (DCs) Stimulation

BALB/c mice were subcutaneously inoculated with 4T1 cells and divided into 6 groups: (1) control, (2) MCP, (3) MCPG, (4) NIR-II laser, (5) MCP + NIR-II, and (6) MCPG + NIR-II. After intravenous injection of 12 h, laser treatment was administered followed by serum collection. The collected samples were stained with anti-mouse CD 80 and CD 86 antibodies and analyzed using flow cytometry to detect their expression levels. Meanwhile, the cytokine levels of interferon γ (IFN-γ) and tumor necrosis factor α (TNF-α) in the samples were quantified using an enzyme-linked immune sorbent assay (ELISA) kit.

### Histological Analysis

Histological tests were performed after 14 days of treatment. Mice from varying treatment groups were sacrificed. Tumors and major organs were then gathered for histopathological examination, and photographs of the final stained sections were obtained.

## Results and Discussion

### Morphology and Composition

**Fig. 1:**
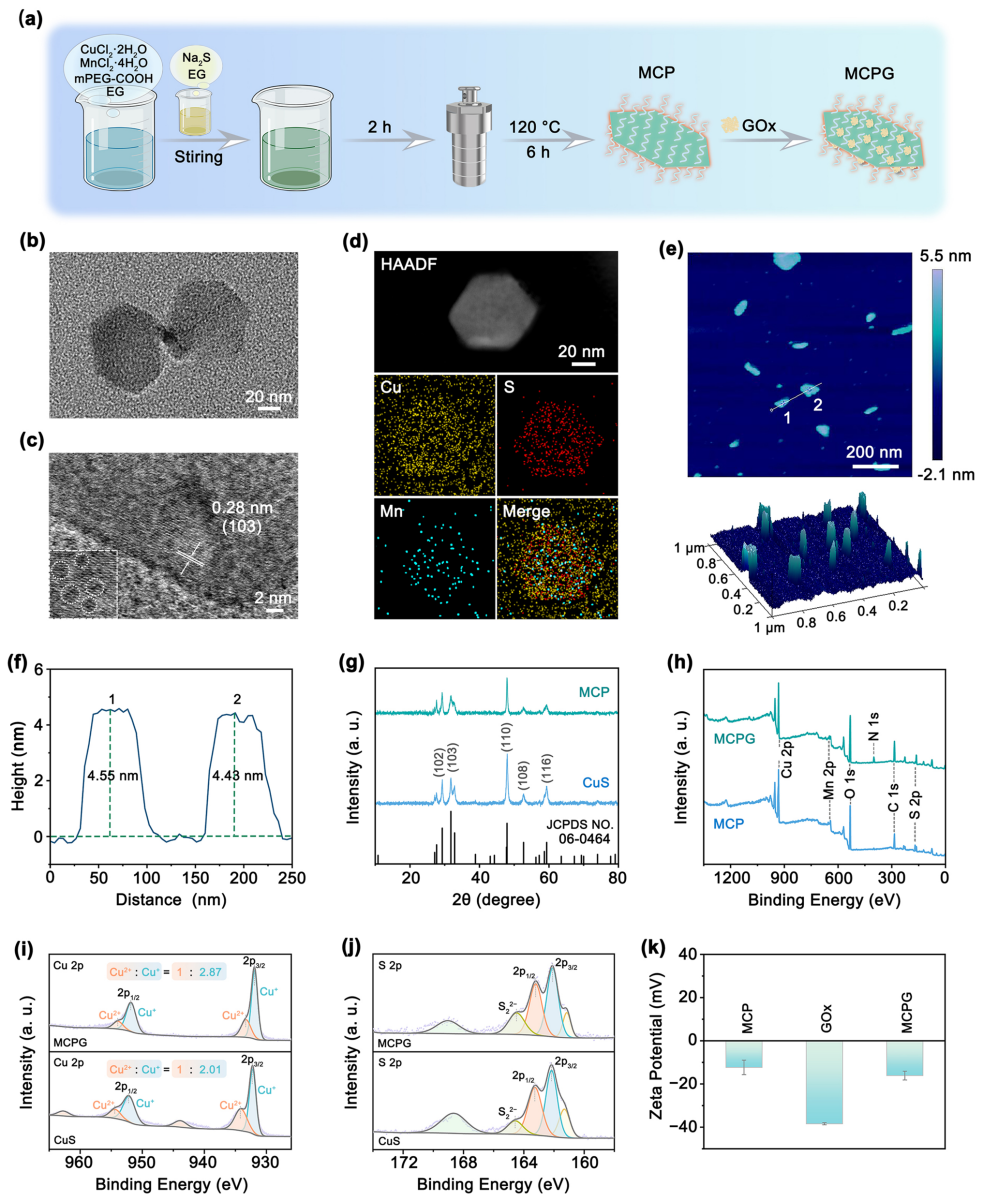
Morphology and composition assessment. **a** Schematic representation of MCPG preparation process. **b** TEM, **c** HRTEM, **d** EDS elemental mapping, **e** AFM image, and **f** the height profiles of MCP. **g** XRD patterns of CuS and MCP. **h** XPS survey spectra of MCP and MCPG. XPS high-resolution spectra of **i** Cu 2*p* and **j** S 2*p* for MCPG. **k** Zeta potentials of MCP, GOx, and MCPG

The main preparation steps for MCPG are shown in Fig. 1a. Initially, ultrathin MCP with S_V_ were prepared using an improved one-pot hydrothermal strategy. The transmission electron microscopy (TEM) image revealed that MCP nanosheets possess a length of approximately 80 nm and a width of about 60 nm (Fig. 1b). In the high-resolution TEM (HRTEM) image, the planar lattice spacing of 0.28 nm can be seen for the cubic phase (103) crystals plane (Fig. 1c). The missing points circled in the HRTEM image indicate atomic defects, revealing the presence of S_V_ on the surface of MCP (inset in Fig. 1c) [10, 42, 43]. The surface defect states on metal sulfides/oxides are crucial for enhancing the chemisorption of molecules [44]. The presence of anion vacancies can modulate the electronic structure and interface properties of MCP. Elemental mapping analysis revealed the uniform dispersion of Cu, S, and Mn on the MCP nanosheets (Fig. 1d). Atomic force microscopy (AFM) images manifested that the thickness of MCP was approximately 4–5 nm (Fig. 1e, f). The crystal structures were examined using X-ray diffraction (XRD) (Fig. 1g). Mn doping did not alter the crystal structure or introduce new impurity peaks. However, the crystallinity of the samples decreases, which can be attributed to the increase in disorder and the distortion of crystal structure during the alloying reaction [10, 42]. On the one hand, the atomic arrangement shifts from an ordered to a disordered state, thereby increasing defects and intensifying the disordered state. On the other hand, due to atomic size differences or internal stress causing lattice distortion, it deviates from the ideal periodicity, resulting in structural deformation.

Upon integration of GOx, the size of MCPG increased slightly compared to MCP (Fig. S1). A clear N signal was observed by X-ray photoelectron spectroscopy (XPS) after loading GOx onto the surface of the MCP (Fig. 1h), and the Cu 2*p* splitting peaks in MCPG shifted toward a lower energy region owing to the transition of Cu^2+^ to Cu^+^ (Figs. 1i and S2). The binding energy of orbital electrons decreases when the atom gains electrons, indicating a lower valence electron state. Consequently, the characteristic peaks were shifted toward a lower binding energy. The characteristic peaks of MCPG located at 954.0 eV (Cu^2+^) and 951.9 eV (Cu^+^) were indexed to the spin-splitting orbits of Cu 2*p*_1/2_, while the distinguished peaks at 933.3 eV (Cu^2+^) and 931.9 eV (Cu^+^) were ascribed to Cu 2*p*_3/2_. The Cu^2+^/Cu^+^ ratios of CuS and MCPG were calculated to be 1/2.01 and 1/2.87, respectively, confirming that the relative content of Cu^+^ increases with Mn substitution. The presence of Cu^+^ is widely acknowledged to induce a charge imbalance, thereby promoting S_V_ formation. Regarding the S 2*p* peaks of MCPG, binding energies at 163.2 and 162.1 eV corresponded to S 2*p*_1/2_ and S 2*p*_3/2_, respectively (Figs. 1j and S3). The peak at the binding energy of 161.1 eV represents the S 2*p* transition. Another peak at 164.5 eV was attributed to S_2_^2−^, indicating the presence of nonstoichiometric sulfide. Specifically, the peak area of S_2_^2−^ in MCPG was higher than that in pristine CuS, demonstrating an increase in the formation of S_V_ on the MCPG surface [45]. The high-resolution Mn 2*p* spectrum can be attributed to Mn^4+^ and Mn^2+^, with a Mn^4+^/Mn^2+^ of 1/1.92 (Fig. S4a). Moreover, the presence of Cu, S, Mn, N, O, and C in the MCPG was confirmed using energy-dispersive X-ray spectroscopy (EDS) (Fig. S4b). Notably, the K_α_ peak at 5.9 keV and the K_β_ peak at 6.5 keV serve as the primary characteristic peaks of Mn. To further confirm the presence of S_V_, electron paramagnetic resonance (EPR) spectroscopy was conducted on CuS and Cu_2_MnS_3-x_ (Fig. S5). After Mn doping, Cu_2_MnS_3-x_ exhibited a more pronounced S_V_ signal at g = 2.003, thereby verifying that the incorporation of Mn can generate abundant S_V_. The zeta potential values of MCP (− 12.32 mV), GOx (− 38.41 mV) and MCPG (− 16.11 mV) were measured, indicating the successful synthesis of MCPG nanosheets (Fig. 1k). The negatively charged surface is beneficial to reduce the blood clearance and promote the accumulation of nanoparticles in tumors, thereby ensuring excellent biocompatibility [46–49]. MCPG exhibited outstanding stability in different solutions even after 48 h of incubation (Fig. S6). Furthermore, the loading efficiency of GOx in MCPG was determined to be 24.62% using the Bradford method (Fig. S7). Thermogravimetric analysis (TGA) was applied to MCP and MCPG under heating conditions ranging from 30 to 600 °C. In comparison with MCP, the weight loss of MCPG was 25.82% owing to the loading of GOx (Fig. S8).

### Photothermal Performance and Enzyme Catalytic Activity

**Fig. 2:**
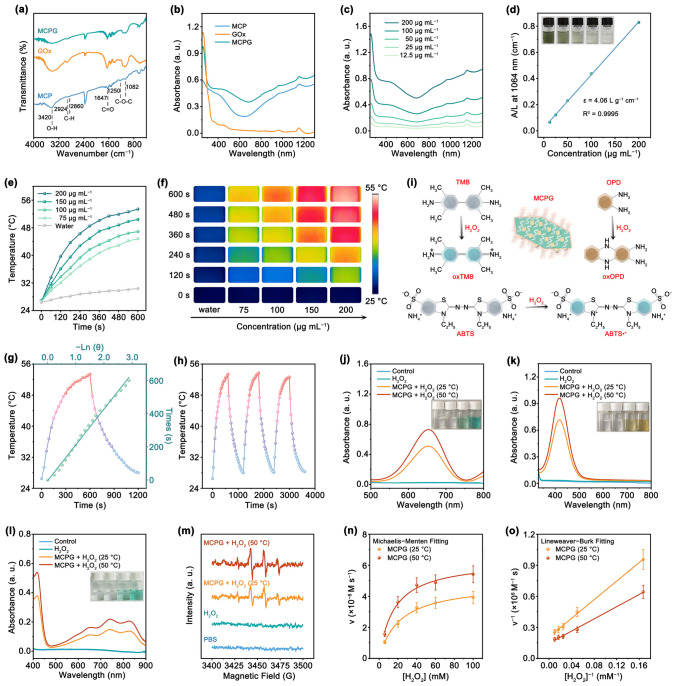
Photothermal performance and enzyme catalytic activity. **a** FT-IR and **b** UV–vis absorption spectra of MCP, GOx, and MCPG. **c** UV–vis absorption spectra of MCPG solution at varying concentrations. **d** The mass extinction coefficient of MCPG at 1064 nm. **e** Photothermal curves and **f** infrared thermal images of MCPG at varying concentrations irradiated with 1064 nm laser. **g** Heating and cooling processes of MCPG solution. **h** Photothermal stability of MCPG irradiated with 1064 nm laser. **i** Schematic illustration of the catalyzed oxidation for TMB, OPD, and ABTS. UV–vis absorption spectra of **j** oxTMB, **k** oxOPD, and **l** oxABTS after different treatments. **m** ESR spectra of various groups to detect •OH production. **n** Michaelis–Menten curves and **o** Lineweaver–Burk plotting of MCPG at 25 °C and 50 °C, respectively

The mPEG–COOH was combined with Cu_2_MnS_3-x_ via Cu-carboxylate coordinative couplings [50]. The broad characteristic peak at 3420 cm^−1^ is ascribed to O–H, while peaks at 2924 and 2860 cm^−1^ are assigned to C–H. The peak at 1647 cm^−1^ is attributed to C = O, and the peaks at 1250 and 1082 cm^−1^ are corresponded to C–O–C. These characteristic peaks signify the existence of mPEG–COOH on Cu_2_MnS_3-x_. For GOx and MCPG, a distinctive band around approximately 1660 cm^−1^ represents amide I of proteins (Fig. 2a) [34, 51]. The UV–vis absorption spectra of MCP, GOx, and MCPG were measured, revealing that the MCPG nanosheets exhibited a significant absorption in the near-infrared II (NIR-II) region (Fig. 2b). Meanwhile, the absorption intensity presented a linear correlation with the concentration of MCPG, indicating the extreme homogeneity of these samples without any aggregation (Fig. 2c). According to the Lambert–Beer law, the *ε* of MCPG at 1064 nm was computed to be 4.06 L g^−1^ cm^−1^ from the fitted curve (Fig. 2d). The photothermal conversion capability of MCPG was examined through the temperature variation curves of MCPG and corresponding images irradiated upon 1064 nm laser (0.7 W cm^−2^) (Fig. 2e, f). The temperature variation of MCPG aqueous solution was found to be concentration-dependent, manifesting a clear correlation between the two factors. The temperature of MCPG solution displayed a distinct upward trend of ascended by 26.9 °C at the 600 s time point, confirming the prominent photothermal conversion ability of MCPG. Meanwhile, the temperature of MCPG aqueous solution was positively correlated with the laser power density (Fig. S9). In addition, the photothermal conversion efficiency of MCPG was computed to be 43.77% (Fig. 2g). The photothermal performance of MCPG manifested no significant change over three heating/cooling cycles, demonstrating the satisfactory thermal stability (Fig. 2h). The results provided compelling evidence of the outstanding photothermal properties of MCPG nanosheets on account of the strong plasma absorption in the NIR-II region arising from the S_V_.

Due to the unique advantage of Cu, we assessed the POD-like enzymatic activity of MCPG by TMB, OPD, and ABTS (Fig. 2i). The peaks intensity increased with increasing temperature, suggesting that the thermal effect promoted the emergence of ·OH (Fig. 2j). Similarly, the OPD (Fig. 2k) and ABTS (Fig. 2l) colorimetric test results were consistent with the aforementioned findings. The peak of oxTMB was distinctly increased at a higher concentration of H_2_O_2_, further illustrating that MCPG indeed possesses POD-like activity (Fig. S10). Afterward, the ·OH production was surveyed by electron spin resonance (ESR) via 5,5-dimethyl-1-pyrroline N-oxide (DMPO) probe (Fig. 2m). It was evident that no detectable signal was produced in both control and H_2_O_2_ groups. Under acidic conditions where MCPG and H_2_O_2_ existed simultaneously, we collected peaks with the intensity ratio of 1:2:2:1, which validated the •OH production. Especially, the peak was increased at 50 °C compared to 25 °C, verifying that elevated temperature promotes the POD-like activity. Subsequently, the impact of hyperthermia on the catalytic characteristics of MCPG was investigated by employing H_2_O_2_ as a substrate (Fig. S11a, b). According to the Michaelis–Menten curves and Lineweaver–Burk plots, the *K*_m_ of MCPG was computed to be 21.69 and 19.67 mM, and the *V*_max_ was 4.83 × 10^−8^ and 6.90 × 10^−8^ M s^−1^ at 25 and 50 °C, respectively (Fig. 2n, o). Compared with other nanozymes, MCPG exhibited a relatively high *V*_max_ level and a low *K*_m_ value (Table S1), which confirmed its excellent catalytic activity. For MCPG substrate, the specific activity value was 0.61 U mg^−1^ (Fig. S11c, d). Furthermore, GOx consumes O_2_ to convert glucose into gluconic acid and H_2_O_2_ [52, 53]. Notably, nanomaterials with CAT-like activity can generate H_2_O and O_2_ via H_2_O_2_. This process provides positive feedback for cascade catalysis. The CAT-like activity of MCPG and the effect of GOx were systematically evaluated using Amplex Red as the detection reagent. In the presence of H_2_O_2_, the characteristic absorption intensity decreased as the concentration of MCPG increased, indicating the effectively consumption of H_2_O_2_ (Fig. S12a). Meanwhile, in the presence of glucose, the characteristic absorption intensity increased as the concentration of MCPG increased, confirming the generation of H_2_O_2_ (Fig. S12b). MCPG produced significant amounts of O_2_ in the presence of H_2_O_2_, and increasing temperature promoted O_2_ emergence. However, with the addition of glucose, the production of O_2_ decreased because the glucose catalyzed by GOx consumed O_2_ (Fig. S13a). In addition, it is widely recognized that heteroatom doping holds significant potential for enhancing the catalytic activity. Compared with CuS-PEG/GOx, the higher oxygen production of MCPG nanosheets can be attributed to the enhanced CAT-like catalytic activity due to doping. Meanwhile, the oxTMB absorption peak of MCP exhibits a notably stronger intensity compared to CuS-PEG (Fig. S13b). In comparison with MCPG, the *K*_m_ of CuS-PEG/GOx was computed to be 25.56 mM, and the *V*_max_ was only 2.38 × 10^−8^ M s^−1^ at 25 °C based on the Michaelis–Menten curves and Lineweaver–Burk plots, indicating the promising opportunity provided by doping in effectively regulating the POD-like activity of nanomaterials (Figs. S14 and S15). The collective findings above suggest that MCPG nanosheets with vacancy defects exhibited a distinguished enzymatic activity, and elevated temperature as well as doping favors the enhancement of its enzymatic activity.

### DFT Calculations

**Fig. 3:**
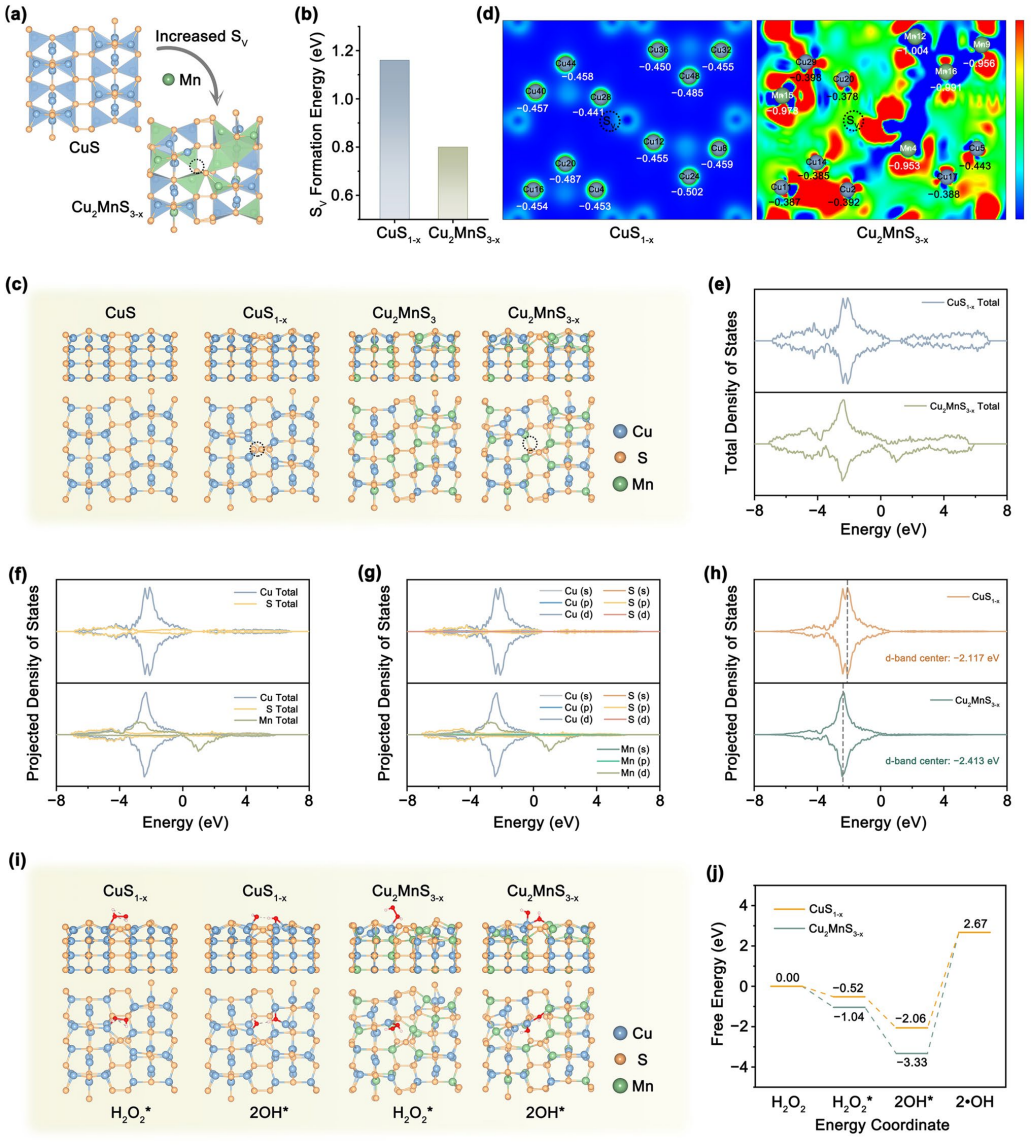
DFT calculations and revelation of enzyme catalysis system. **a** Schematic illustration of the S_V_ formation in Cu_2_MnS_3-x_. **b** Calculated S_V_ formation enthalpy of CuS and Cu_2_MnS_3-x_. **c** Optimized structure for ideal CuS, CuS_1-x_, Cu_2_MnS_3_, and Cu_2_MnS_3-x_ with (110) crystal facet. **d** The optimized differential charge density of CuS_1-x_ and Cu_2_MnS_3-x_ as well as corresponding effective charges of surface atoms calculated by Bader analysis program. **e–g** Total DOS and PDOS profiles. **h** Cu 3*d* states of CuS_1-x_ and Cu_2_MnS_3-x_. **i** The surface structure under the initial state (IS, H_2_O_2_) and final state (FS, 2OH*) of CuS_1-x_ and Cu_2_MnS_3-x_ with (110) crystal facet during the POD-like catalysis process. **j** Energy charts of the POD-like catalysis pathways of Cu_2_MnS_3-x_ with (110) crystal facet

The VASP was utilized for DFT calculations to evaluate the catalytic mechanisms underlying the exceptional POD simulation activity. Since Mn substitution could expedite structural reconstruction, a unique Mn − S − Cu arrangement was constructed at the surface (Fig. 3a). The energy required for S_V_ formation on the surface of ideal CuS_1-x_ is 1.16 eV, whereas on the Cu_2_MnS_3-x_ surface is significantly reduced to 0.80 eV, verifying that S_V_ is more easily formed after Mn doping (Fig. 3b). Four computational surface models, namely ideal CuS, CuS_1-x_, Cu_2_MnS_3_, and Cu_2_MnS_3-x_, were established to elucidate their microscopic electronic structures (Fig. 3c). Cu28 and Cu20 act as active sites for CuS_1-x_ and Cu_2_MnS_3-x_ to adsorb H_2_O_2_, respectively (Fig. 3d). Cu_2_MnS_3-x_ exhibited a higher affinity for H_2_O_2_ adsorption than CuS_1-x_ because of its small absolute value. In addition, CuS_1-x_ and Cu_2_MnS_3-x_ were used to examine the influence of doping on the projected density of states (PDOS) (Fig. 3e–g). Cu_2_MnS_3-x_ exhibited a narrower band than CuS_1-x_, which was primarily attributed to the additional defect levels. These defect states can effectively reduce the band gap, consequently lowering the energy required for electron transitions. The d-band centers of Cu on various surfaces were determined based on the PDOS of the Cu 3*d* spectrum (Fig. 3h). The d-band center of Cu for Cu_2_MnS_3-x_ exhibited a significant downward shift, which can be ascribed to the formation of abundant S_V_ with Mn replacement. This substitution remarkably enhanced the adsorption energy of the intermediates, thereby promoting the catalytic activity.

Based on the differences in crystal facets of Cu_2_MnS_3-x_, three surface models exposing the (102), (103), and (110) crystal facets were constructed to elucidate the catalytic mechanism (Fig. S16). To assess the electron transfer ability between the catalyst surface and the intermediate, the work functions of different crystal facets were measured (Fig. S17). Regarding the redistribution of surface charges, the work functions of the (102), (103), and (110) crystal facets are 5.00, 5.29, and 4.73 eV, respectively. These values suggested that the sequence of electron transfer from the catalyst surface to the intermediate follows the order: (110) > (102) > (103). To elucidate the corresponding reaction mechanism from a thermodynamic perspective, a comprehensive investigation was conducted on the intricate POD-like enzymatic catalytic processes using the optimized surfaces. According to the intermediates formed during the continuous reaction steps in the POD-like activity, all intermediates involved in the H_2_O_2_ catalytic reaction were systematically optimized for the (102), (103), and (110) crystal facets (Fig. S18). The computed free energy diagram indicates that the initial step entails the capture and adsorption of H_2_O_2_ onto the surface of Cu_2_MnS_3-x_. Subsequently, H_2_O_2_ decomposes into two OH* species, which is thermodynamically favorable on all three crystal facets. The third step entails the release of adsorbed OH* and the formation of ·OH radicals, which requires overcoming an energy barrier and thus serves as the rate-determining step (Fig. S19). Compared with the (102) and (103) crystal facets of − 0.90 and − 2.32 eV, the (110) crystal facets exhibit the lowest adsorption energies (Δ*G*_ads_) of − 3.33 eV, which display superior POD-like activity for the (110) crystal facet. Since a lower work function facilitates electron transfer from the surface to the reactants, the (110) crystal facet exhibits a stronger electron-donating capability, which is more favorable for promoting the activation of intermediates. Therefore, the (110) crystal facet was selected for further investigation of catalytic activity in doping.

To further investigate the influence of Mn doping and S_V_ defects, we examined the main intermediate conformations and the corresponding free energy diagrams of the POD-like processes for the CuS_1-x_, Cu_2_MnS_3_, and Cu_2_MnS_3-x_ (Figs. 3i, j and S20). The Δ*G*_ads_ of H_2_O_2_ molecules on CuS_1-x_, Cu_2_MnS_3_, and Cu_2_MnS_3-x_ are − 0.52, − 0.29, and − 1.04 eV, respectively. In comparison, H_2_O_2_ was easily adsorbed onto the surface of Cu_2_MnS_3-x_ because the energy required was low. The Δ*G*_ads_ required for 2OH* generation on the surface of Cu_2_MnS_3-x_ is only − 3.33 eV, which is lower than the − 2.06 eV of CuS_1-x_ and − 2.72 eV of Cu_2_MnS_3_. These results indicate that Mn doping and the formation of sufficient S_V_ defects hinder the stabilization of H_2_O_2_ molecules on the Cu_2_MnS_3-x_ surface, consequently leading to their direct dissociation into low-energy 2OH*. Thus, the substitution of Mn as well as the reconstruction and evolution of S_V_ results in a remarkable increase in the POD-like enzymatic catalytic activity of Cu_2_MnS_3-x_.

### Degradation Characteristics and PTEC Mechanism

**Fig. 4:**
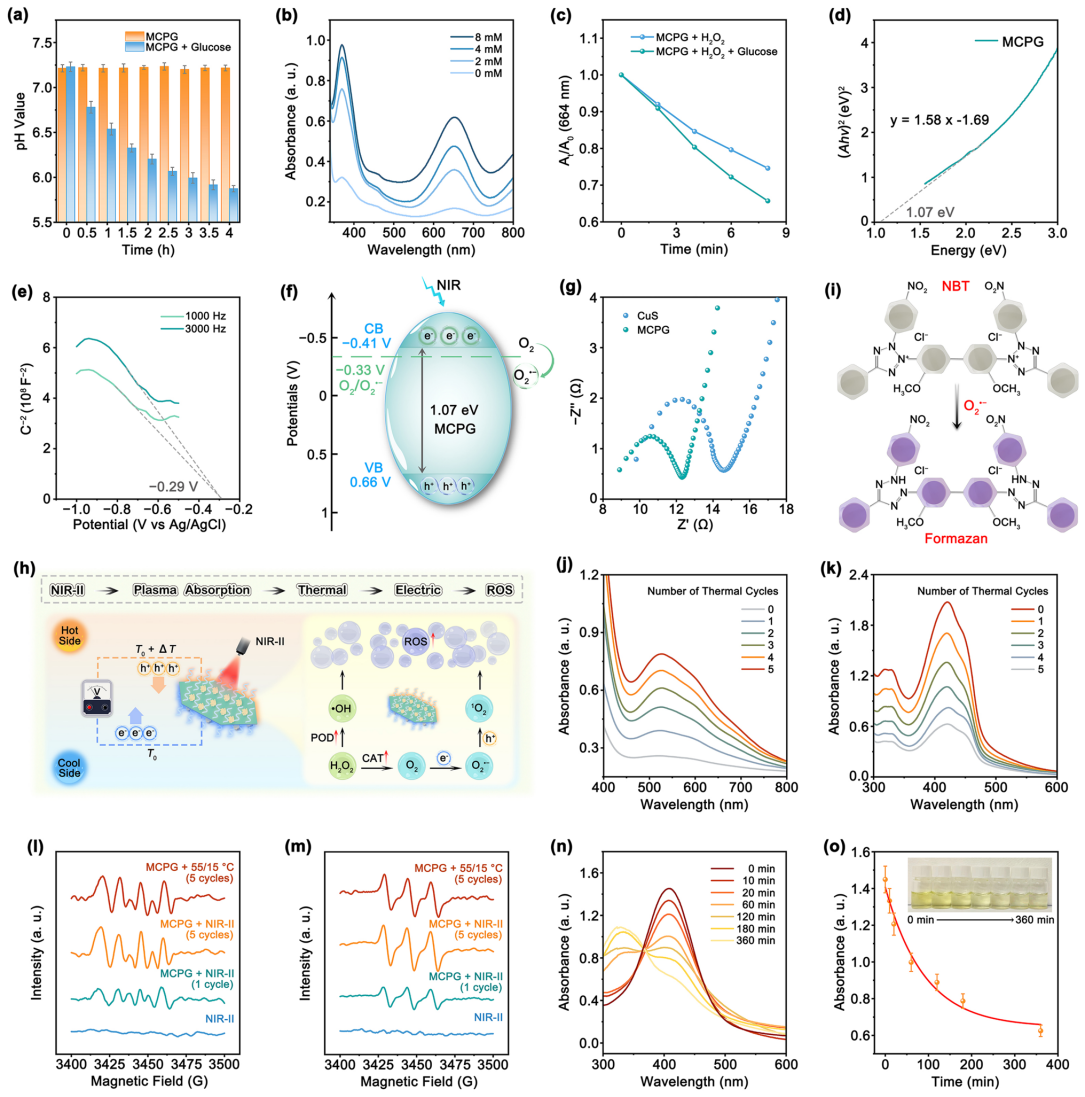
Degradation characteristics and PTEC mechanism. **a** pH variation of MCPG solutions with or without glucose addition. **b** oxTMB spectra cultivated with MCPG and various glucose concentrations. **c** Effects of glucose addition or omission on the degradation of MB. **d** Tauc plot of MCPG according to UV–vis diffuse reflectance spectrum. **e** Mott–Schottky plot of MCPG nanosheets. **f** Schematic representation on the energy bands structure of MCPG. **g** EIS Nyquist plots of CuS and MCPG. **h** Schematic representation of PTEC mechanism. **i** Schematic diagram of the redox reaction of NBT with O_2_^•−^. UV–vis absorption spectra of **j** NBT and **k** DPBF of laser irradiation cycle in MCPG solutions. ESR spectra for the examination of **l** O_2_^•−^ and **m** ^1^O_2_. **n, o** Time-dependent GSH depletion by MCPG

The pH value of MCPG solution decreased drastically after glucose addition, which indicated that MCPG nanosheets facilitated the oxidation of glucose into gluconic acid (Fig. 4a). After that, TMB was employed as an indicator to monitor the changes in oxTMB absorption peak intensity at various glucose concentrations without H_2_O_2_ addition (Fig. 4b). The ascension of absorbance with the rising glucose concentration further illustrated the ability of MCPG to catalyze glucose to produce H_2_O_2_. Similar results were obtained from Methylene blue (MB) degradation experiments (Figs. S21 and 4c). In the presence of glucose, the absorbance of the solution at 664 nm decreased more markedly, suggesting that MCPG catalyzes the conversion of glucose and O_2_ into H_2_O_2_, which subsequently facilitates the production of ·OH.

The vacancy defects can effectively capture electrons or holes, thereby improving the carrier separation efficiency and inhibiting photogenerated carrier recombination [1]. To begin with, the band gap energy (*E*_g_) of MCPG was obtained based on the Kubelka–Munk (K-M) formula derived from UV–vis diffuse reflectance spectroscopy, and the band gap value of MCPG was computed to be 1.07 eV (Fig. 4d). The valence band (VB) position of MCPG was appraised via high-resolution VB XPS spectra, and the deviation between the Fermi energy level (*E*_f_) and the *E*_VB_ can be determined to be 0.34 eV (Fig. S22). Based on the Mott–Schottky plots, MCPG displays characteristics of a *p*-type semiconductor because of the negative slopes (Fig. 4e). The flat band potential versus Ag/AgCl (*E*_fb_ vs Ag/AgCl) of MCPG is − 0.29 V, and *E*_fb_ versus the normal hydrogen electrode (NHE) can be calculated as follows:

8 $$E_{{{\text{fb}}}} \left( {vs{\text{NHE}}} \right) = E_{{{\text{Ag}}/{\text{AgCl}}}} + E_{{{\text{fb}}}} \left( {vs{\text{Ag}}/{\text{AgCl}}} \right) + 0.0{59} \times {\text{pH}}$$

where *E*_Ag/AgCl_ is 0.199 V at 25 °C, and the pH of the Na_2_SO_4_ solution is 7. The acquired *E*_fb_ (*vs* NHE) value for MCPG is 0.32 V. Thus, the VB of MCPG was computed to be 0.66 V. The conduction band (CB) potential was then determined to be − 0.41 V based on the formula *E*_CB_ = *E*_VB_ − *E*_g_ (Fig. 4f). This result manifested that nanosheets have the potential to form O_2_^•−^ under 1064 nm laser excitation, which are ascribed to the redox potential of O_2_/O_2_^•−^ (− 0.33 V). Doping not only increases the carrier density but also enhances the electrical conductivity [10]. Incorporating doped atoms with few valence electrons generates holes, whereas introducing doped atoms with valence electrons donates free electrons [54]. The small arc diameter of MCPG in electrochemical impedance spectroscopy (EIS) results in a low charge transfer resistance (Fig. 4g). The thermoelectric effect leads to the formation of an intrinsic electric field in MCPG, thereby facilitating a high electron transfer rate. Consequently, Mn doping and the resulting S_V_ can remarkably augment the density of photogenerated carrier and enhance the e^−^–h^+^ separation efficiency, ultimately enhancing the catalytic reaction process.

To explain the In vitro thermoelectrically coupled enzyme catalytic activity of MCPG, the PTEC mechanism was investigated based on the plasmon heat and the Seebeck effect (Fig. 4h). Upon absorption of 1064 nm photons on MCPG nanosheets, plasmon heat and temperature gradient act as driving forces for the directional movement of e^−^ and h^+^. As a *p*-type semiconductor, MCPG tends to provide holes that contribute to carrier diffusion during the heating/cooling cycle, leading to the emergence of an intrinsic electric field. The electric field is used to amplify the separation and transport of e^−^–h^+^, which are subsequently directed toward the surface of MCPG nanosheets for further participation in catalytic reaction. Eventually, electrons in CB effectively catalyze the formation of O_2_ to O_2_^•−^, whereas the holes generated in VB facilitate oxidation reaction, further driving the conversion of O_2_^•−^ into ^1^O_2_. Subsequently, the MCPG nanosheets were irradiated with a 1064 nm laser and then cooled naturally to room temperature (25 °C). The underlying mechanism of PTEC was investigated using five on/off laser cycles. NBT was used to assess O_2_^•−^ levels produced by PTEC (Fig. 4i). As the number of cycles increased, the characteristic peak intensity gradually increased (Fig. 4j). Subsequently, a series of steps of NIR-II → plasma absorption → thermal → electric → ROS was validated by employing DPBF indicator (Fig. 4k). This phenomenon can be attributed to the outstanding thermoelectric performance of MCPG nanosheets, which facilitates the efficient carrier separation and migration. In addition, considering the crucial role of temperature variation in TECT, a water bath was used to simulate a fluctuating temperature environment and assess ROS formation (Fig. S23). Rapid temperature fluctuations (d*T*/d*t*) can induce substantial thermal differences, thereby generating thermoelectric signals. MCPG nanosheets exhibited DPBF degradation under hot water bath, indicating an enhanced capability to generate O_2_^•−^ and ^1^O_2_ with the increases in heating/cooling cycles. Notably, the absorbance values changed consistently for each additional cycle, confirming the stable PTEC of the MCPG nanosheets. Moreover, the ESR spectra evidenced the sextuplet and triplet peaks that corresponded to the O_2_^•−^ and ^1^O_2_ species, further implying that MCPG could generate O_2_^•−^ and ^1^O_2_ during alternating hot and cold cycles (Fig. 4l, m).

Thermoelectric materials exhibit the capability to transform heat into electricity. During the process of thermoelectric conversion, the Seebeck coefficient (*S*) quantifies the capability of a thermoelectric material to transform a temperature gradient into a voltage, mathematically expressed as *S* =  − Δ*V*/Δ*T*. To fully evaluate the efficiency of thermoelectric conversion, dimensionless figure of merit (*ZT*) is described as *ZT* = *S*^2^⋅σ *T*/κ, where *S* denotes the Seebeck coefficient, *T* is the absolute temperature, κ is the thermal conductivity, and *σ* is the electrical conductivity. *PF* can be represented by* S*^2^⋅*σ*. In general, there are various approaches to enhancing the value of *ZT*, such as doping techniques, material advancements, decoupling thermoelectric parameters, and engineering band structure modifications [55–57]. MCP is a material with abundant S_V_, which facilitates the regulation of its thermoelectric performance. First, the thermoelectric performance of MCP was evaluated after spark plasma sintering (SPS). The MCP nanosheets demonstrate satisfactory conductivity across a temperature range of approximately 309–375 K, with a low resistivity *ρ* of 1.05 mΩ cm at 309 K (Fig. S24a). By taking the reciprocal of the resistivity, the *σ* of MCP is obtained to be 95.09 × 10^3^ S m^−1^ (Fig. S24b). The *ρ* exhibits a rapid increase in the high temperature region, reaching 1.13 milliohm·cm at 375 K, where *σ* is determined to be 88.47 × 10^3^ S m^−1^. The positive *S* value certifies that MCP functions as a *p*-type semiconductor, and the *S* value rises to 14.10 μV K^−1^ with ascending temperature (Fig. S24c). Based on the *σ* and *S* values, the *PF* is obtained to be 16.25 and 17.59 μW m^−1^ K^−2^ at 309 and 375 K, respectively (Fig. S24d). The thermal transport performance of the MCP was measured at 298, 313, 333, 353, and 373 K, including the specific heat capacity (*C*_P_), thermal diffusivity (*D*), and thermal conductivity (κ) (Fig. S25a–c). The *D* of MCP nanosheets decreases with ascending temperature, while the *κ* increases with temperature. The *ZT* value of MCP was found to be 0.0035 at a low sintering temperature of approximately 375 K, demonstrating that the MCP nanosheets exhibited thermoelectric performance (Fig. S25d).

Excess endogenous GSH can consume ROS and inhibit antitumor effects. The peak of 5,5′-dithiobis-(2-nitrobenzoic acid) (DTNB) decreased distinctly, demonstrating successful depletion of GSH by MCPG (Figs. 4n, o and S26). XPS analysis suggested that the ratio of Cu^2+^/Cu^+^ and Mn^4+^/Mn^2+^ in the original MCPG was 1/2.87 and 1/1.92, which transformed into 1/3.48 and 1/2.43 after reaction with GSH (Fig. S27). Furthermore, the release rates of Cu and Mn from MCPG were detected under various pH conditions in the simulated body fluid, validating that H_2_O_2_ and acidity are sufficient conditions for the biodegradation of MCPG nanosheets (Figs. S28 and S29).

### In Vitro Antitumor Capability

**Fig. 5:**
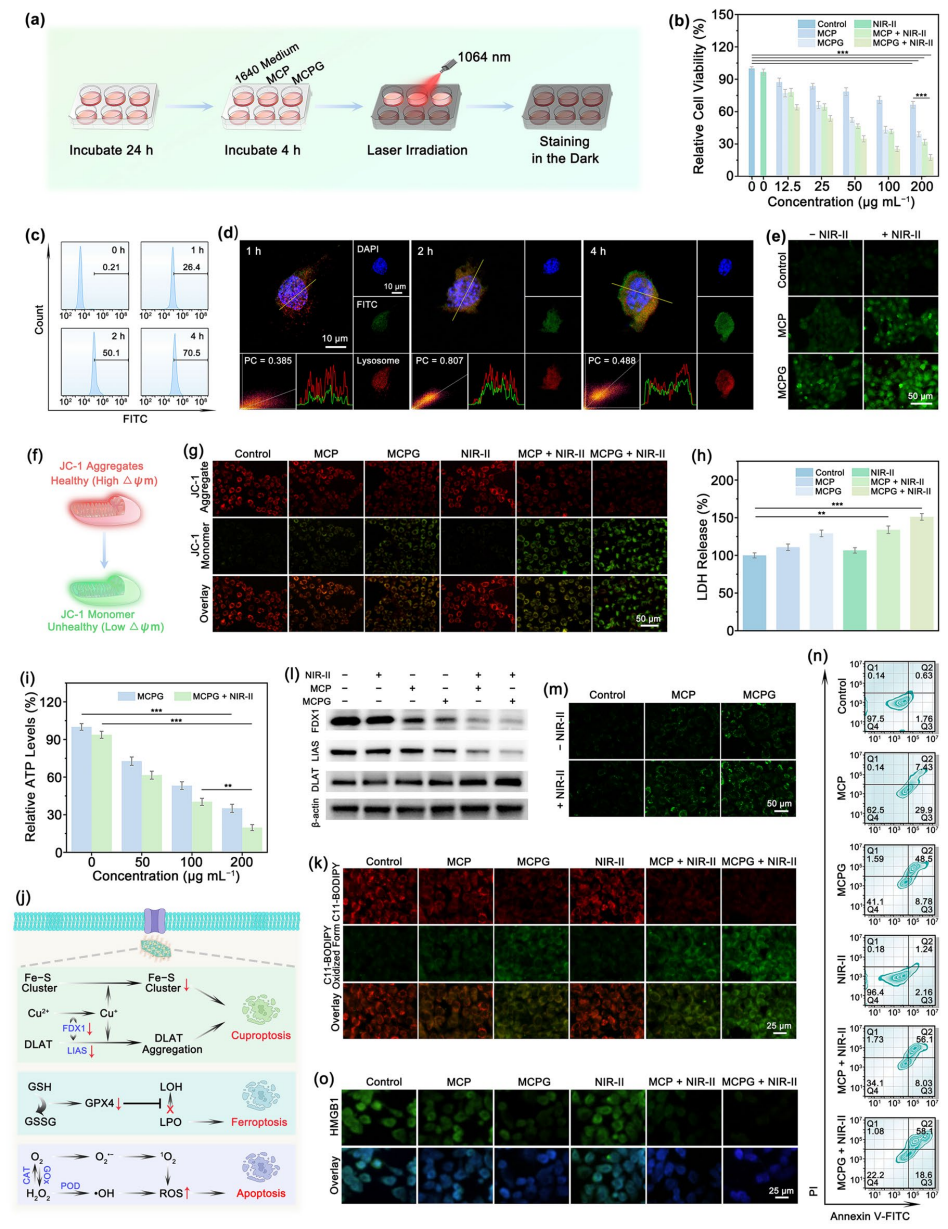
In vitro antitumor efficacy. **a** Schematic representation of cellular therapy. **b** Cell viability of 4T1 cells after different treatments. **c** Flow cytometry assay of FITC-modified MCPG at various treatment periods. **d** CLSM images for the colocalization of FITC-modified MCPG with lysosome. **e** Intracellular ROS assessment imaged via fluorescence images. **f** Chromogenic mechanism of JC-1. **g** JC-1 assays of 4T1 cells. **h** Quantitative analysis of LDH release after various treatments. **i** Relative percentage of intracellular ATP after being treated with different formulations. **j** Schematic illustration of MCPG triggered cuproptosis/ferroptosis/apoptosis. **k** C11-BODIPY^581/591^ staining assays of 4T1 cells. **l** Western blot assay of FDX1, LIAS, and DLAT in various conditions. **m** DLAT images of cells under varying incubation. **n** The apoptosis ratio of cells measured by flow cytometry. **o** HMGB1 expression in different groups. Data were presented as mean ± S.D. (*n* = 3). Statistical significance is evaluated by a two-tailed Student’s t test, **p < 0.01, ***p < 0.001

Based on the prominent performance of MCPG nanosheets, we appraised the therapeutic efficacy (Fig. 5a). To begin with, the biocompatibility and cytotoxicity of MCPG on L929 fibroblast cells, 4T1 breast cancer cells, HepG2 hepatic carcinoma cells, and A549 lung carcinoma cells were estimated via the MTT assay. For L929 cells, the cells were incubated with various concentrations of MCPG (0–200 μg mL^–1^) or exposed to 1064 nm laser with different power densities (0–1.5 W cm^–2^), demonstrating that MCPG nanosheets exhibited excellent biocompatibility and no significant toxic side effects (Figs. S30 and S31). For 4T1 cells, HepG2 cells, and A549 cells, the relative cell viability of MCP was markedly lower than control and NIR-II groups and further decreased upon 1064 nm laser irradiation. MCPG + NIR-II group had the lowest relative cell viability and displayed a remarkable cell damage capacity due to the synergistic effect (Figs. 5b and S32, S33). The above results certify the broad therapeutic applicability of MCPG nanosheets. To further investigate the specific antitumor mechanism of MCPG, a subsequent study was conducted employing 4T1 cells as the model system. Upon incubation of 4T1 cells with FITC-labeled MCPG for various durations, the green fluorescence intensity increased gradually with the incubation time prolonged, implying that MCPG nanosheets were endocytosed by 4T1 cells (Fig. S34); the similar results were certificated by flow cytometry (Fig. 5c). Phagocytosis is the predominant mechanism of cellular uptake, and its associated metabolism primarily occurs through lysosomal pathways. The subcellular colocalization of MCPG was analyzed via ImageJ software and confocal laser scanning microscopy (CLSM) employing a lysosomal probe (Fig. 5d). The cellular uptake of FITC-labeled MCPG was monitored following a 1 h incubation period, and the highest colocalization rate between MCPG and LysoTracker was observed after 2 h of cultivation. The Pearson’s coefficient (PC) value ascended from 0.385 at 1 h to 0.807, which illustrated a steady internalization of MCPG through 4T1 cells. Following a treatment duration of 4 h, the green fluorescence of FITC-labeled MCPG dissociated from the red fraction of LysoTracker. The PC value decreased to 0.488, revealing that the MCPG effectively escaped from the endosomes. The above processes are essential to realize the antitumor therapy of MCPG nanosheets.

Intracellular ROS generation was measured via DCFH-DA (Fig. S35). Control and NIR-II groups displayed negligible green fluorescence. Owing to the existence of Cu^+^ and Cu^2+^, POD-like activity of MCP and MCPG nanosheets facilitated the ROS production. Since GOx can catalyze the conversion of glucose to produce H_2_O_2_, the MCPG group demonstrated a high fluorescence signal compared to MCP group. The green fluorescence of the MCPG + NIR-II group was the most pronounced, confirming that the MCPG + NIR-II group possessed superior ROS generation capability due to PTEC, photothermal-enhanced enzyme catalytic, and starvation therapy (Fig. 5e). The fluorescence intensity was semi-quantitatively analyzed by ImageJ software. The results of the MCPG + NIR-II group were 8.0 times higher than those of the control group and 1.6 times higher than those of the MCPG group (Fig. S36). Additionally, to investigate the impact of GOx loading, we discovered that the cell viability of MCPG group was further reduced in the presence of glucose medium compared to that in the absence of glucose (Fig. S37a). As oxygen is required for glucose catabolism via GOx, the rate of MCPG-induced cell damage was attenuated under hypoxic conditions (Fig. S37b). After 4 h of incubation under normoxic conditions, the intracellular glucose level decreased by 28.2% compared to that in the control group, which was diminished by 18.6% under hypoxia compared to that in the control group (Fig. S38). Hence, MCPG can be used as an effective starvation therapy after GOx loading. Furthermore, compared to the control group, the red fluorescence intensity of [Ru(dpp)_3_]Cl_2_ in the MCP group decreased because of CAT-like activity. Conversely, the fluorescence of the MCPG group was slightly enhanced, illustrating the effective consumption of O_2_ by GOx (Fig. S39).

Because mitochondria are critical for cell apoptosis, we measured the mitochondrial membrane potential (ΔΨm) via JC-1 staining kit (Fig. 5f). 4T1 cells incubated with MCPG and NIR-II laser irradiation showed the most pronounced green fluorescence, verifying that the mitochondrial membrane potential was largely depolarized (Fig. 5g). The ratio of green monomers to red aggregates was semi-quantitatively analyzed using ImageJ software. The results indicated the value in the MCPG + NIR-II group was 1.9 times higher than that of MCPG group, certifying the substantial mitochondrial damage (Fig. S40). To assess the integrity of the cell membrane, a LDH test was performed (Fig. 5h). The MCPG + NIR-II group demonstrated significant leakage, which was 1.5 times higher than that of the control group. Glucose is widely acknowledged as a pivotal nutrient for tumor proliferation and can be used to inhibit glycolysis, thereby attenuating intracellular ATP levels. Intracellular ATP levels decreased with increasing sample concentrations, and the inhibition efficiency was notable upon laser irradiation, validating the induction of starvation treatment by glucose consumption (Fig. 5i).

Encouraged by the anticipated antitumor efficacy of MCPG, we further explored the manifestations of cuproptosis, ferroptosis and apoptosis. A schematic diagram depicts the synergistic treatment of cuproptosis/ferroptosis/apoptosis through the perturbation of intracellular redox metabolic dysregulation (Fig. 5j). To initiate, the quantification of intracellular GSH consumption by various concentrations of MCPG was performed via a GSH assay kit. The GSH content exhibited a significant concentration-dependent decrease upon laser irradiation, which was probably related to the enhancement of oxidative stress (Fig. S41). This deficiency can lead to glutathione peroxidase 4 (GPX4) deactivation, thereby promoting ferroptosis [58–61]. Meanwhile, the reduction in GPX4 activity impairs the transformation of toxic lipid hydroperoxides (LPO) to non-toxic hydroxyl compounds (LOH). The C11-BODIPY^581/591^ probe was inserted into the lipid membrane and oxidized by LPO (Fig. 5k). The MCPG + NIR-II group manifested prominent green fluorescence, suggesting the efficient accumulation of LPO. Consistent results were received via the Liperfluo probe (Fig. S42). The regulation of cell death through cuproptosis is a distinctive mechanism characterized by an excess of copper ions and lipidated DLAT aggregation [62, 63]. In general, excessive Cu^2+^ is enzymatically reduced to toxic Cu^+^ via FDX1, a mitochondrial enzyme known for its exceptional reducing capacity and regulating DLAT lipoacylation. Cu^+^ then directly attaches to the lipidated terminus of DLAT, resulting in the aggregation of lipidated DLAT. The hallmarks of FDX1, LIAS, and DLAT were explored using western blotting (Fig. 5l). FDX1 and LIAS serve as upstream regulators of protein lipidosis by facilitating mitochondrial protein aggregation and inducing Fe–S cluster depletion. The MCPG + NIR-II group exhibited significantly disrupted FDX1 and LIAS stability, leading to DLAT oligomerization. Elevated DLAT levels elicit proteotoxic stress, ultimately triggering the cuproptosis. Oligomerization of DLAT was also investigated using fluorescence imaging (Fig. 5m). Cells in the control and NIR-II groups exhibited minimal DLAT, whereas cells in the MCPG + NIR-II group displayed prominent DLAT. In particular, the relationship between the PTEC and cuproptosis of MCPG is considerable research value. The produced ROS during the PTEC process can target biological macromolecules, including the cell membrane and proteins, thereby inducing cellular damage. ROS can further amplify the redox stress response triggered by copper ions, exacerbate intracellular metabolic disturbances, and enhance the cuproptosis. Additionally, the localized high temperature produced by PTEC can alter the permeability and fluidity of tumor cell membranes, promote the uptake and intracellular distribution of copper ions, and further potentiate the cuproptosis.

The ability of the nanosheets to induce apoptosis was further appraised via flow cytometry (Figs. 5n and S43). The apoptosis rate in the MCPG + NIR-II group was 76.7%, which was remarkably higher than those in the MCP (37.3%), MCPG (57.3%), and MCP + NIR-II groups (64.1%). Similarly, the flow cytometry results were confirmed by Calcein-AM/Propidium Iodide (PI) co-staining analysis (Fig. S44). In addition, to investigate the synergistic effect of GOx in MCPG nanosheets, we evaluated the viability of 4T1 cells treated with GOx alone (Fig. S45a). The results indicated that the cytotoxic effect of GOx alone was limited. Similarly, the substantial green fluorescence observed in the Calcein-AM/PI co-staining analysis corroborates these findings (Fig. S45b). Photothermal-amplified tumor treatment not only promotes cell apoptosis, but also induces an immune response via eliciting ICD [64, 65]. The key hallmark of ICD is HMGB1, a highly conserved nuclear protein [66]. HMGB1 can be liberated from the nucleus upon cellular destruction, followed by inducing the discharge of pro-inflammatory cytokines via mononuclear macrophages to stimulate DCs maturation [67, 68]. Meanwhile, proinflammatory cytokines can also stimulate HMGB1 secretion [69]. Compared to the control and NIR-II groups, cells in the MCP group displayed slight green fluorescence, whereas the fluorescence in the MCPG + NIR-II group was negligible (Fig. 5o). Consequently, these results imply that MCPG could commendably penetrate into multicellular solid tumors upon NIR-II laser stimulation to trigger PTEC, photothermal-enhanced enzyme catalysis, and starvation therapy. Synergistic therapy can effectively trigger cuproptosis/ferroptosis/apoptosis, stimulate the immune system, and induce an immune response.

### In Vitro and In Vivo Imaging Performance

**Fig. 6:**
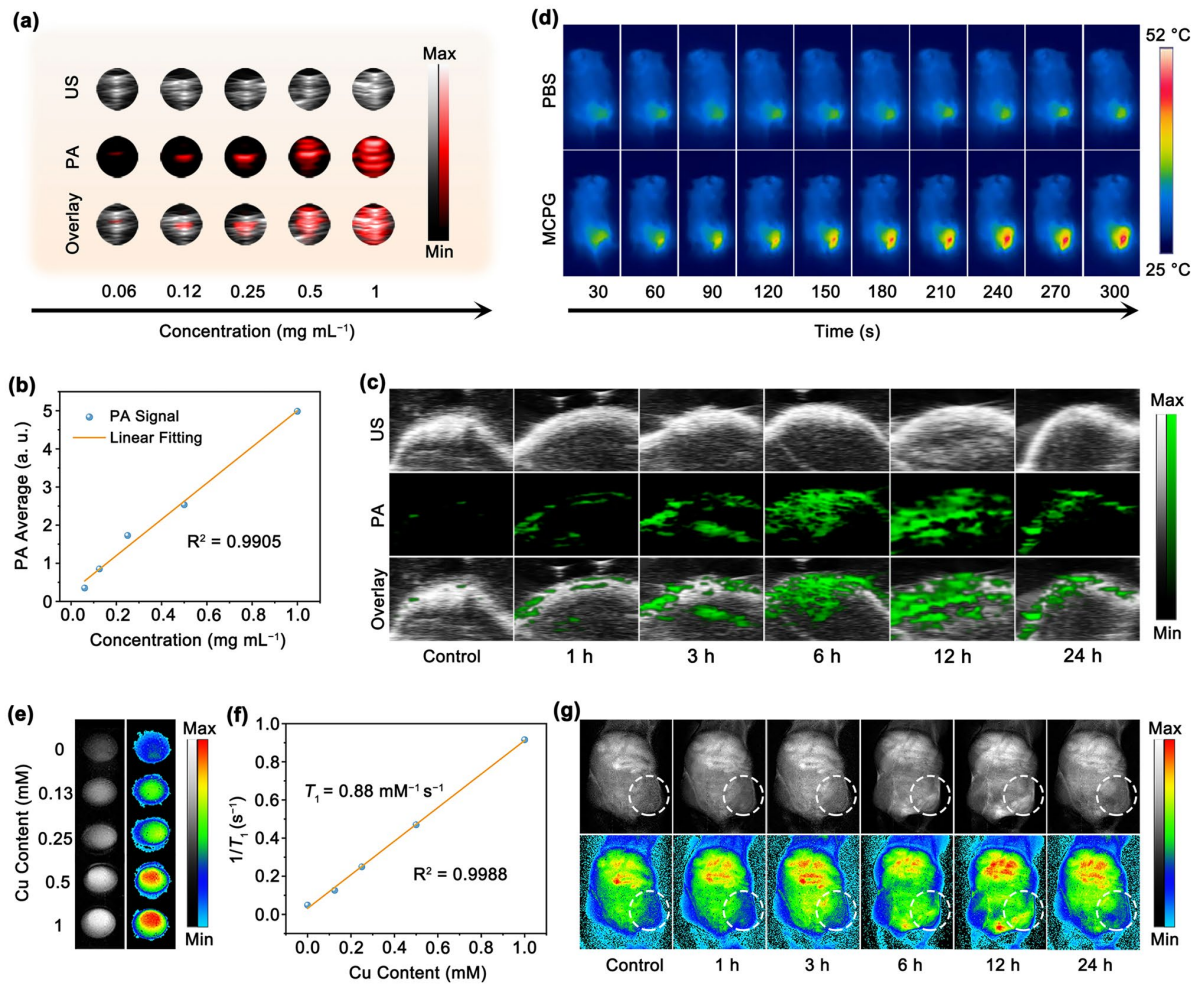
Imaging performance of MCPG. **a, b** In vitro PA images and the linear fitting *versus* the concentrations of MCPG. **c** In vivo PA images of MCPG at various post-injection times. **d** Infrared thermal images at 12-h post-injection of MCPG and PBS. **e** In vitro MR images and **f** *T*_1_ relaxation rates of MCPG with various concentrations. **g** In vivo* T*_1_-weighted MR images of MCPG at varying post-injection time points

Given the robust photothermal characteristics of the MCPG nanosheets, we assessed the potential of MCPG with various concentrations for In vitro PA imaging (Fig. 6a). The remarkable PA signal of MCPG was achieved, and the enhanced signal intensity was observed when the concentration increased (Fig. 6b). Subsequently, after the administration of MCPG, PA images of tumor sites were captured at 0, 1, 3, 6, 12, and 24 h post-injection (Fig. 6c). The signal intensity peaked at 12 h after intravenous injection due to the MCPG accumulation. However, as the injection time prolonged, the signal strength diminished due to the elimination of MCPG from tumor tissue. It can be concluded that laser irradiation at 12-h post-injection is optimal, and MCPG holds promise as a PA imaging agent. After 12 h of MCPG injection, the tumor temperature escalated to 51.7 °C within 10 min exposed to NIR-II laser (Figs. S46 and 6d). In addition, owing to the presence of copper (II) with unpaired electrons, it can serve as a contrast agent for *T*_1_-weighted MR imaging [70]. Thus, we investigated the *T*_1_-weighted MR imaging performance of MCPG nanosheets (Fig. 6e). As the concentration of MCPG ascended, the MR imaging signal intensity was gradually enhanced with a longitudinal relaxivity (*r*_1_) of 0.88 mM^−1^ s^−1^ (Fig. 6f). After the intravenous injection of MCPG nanosheets, the mice exhibited the maximum signal intensity at 12 h (Fig. 6g). Therefore, the application of MCPG demonstrated a remarkable effect in PA and MR imaging-guided tumor synergistic therapy.

### In Vivo Anticancer Therapy Efficacy

**Fig. 7:**
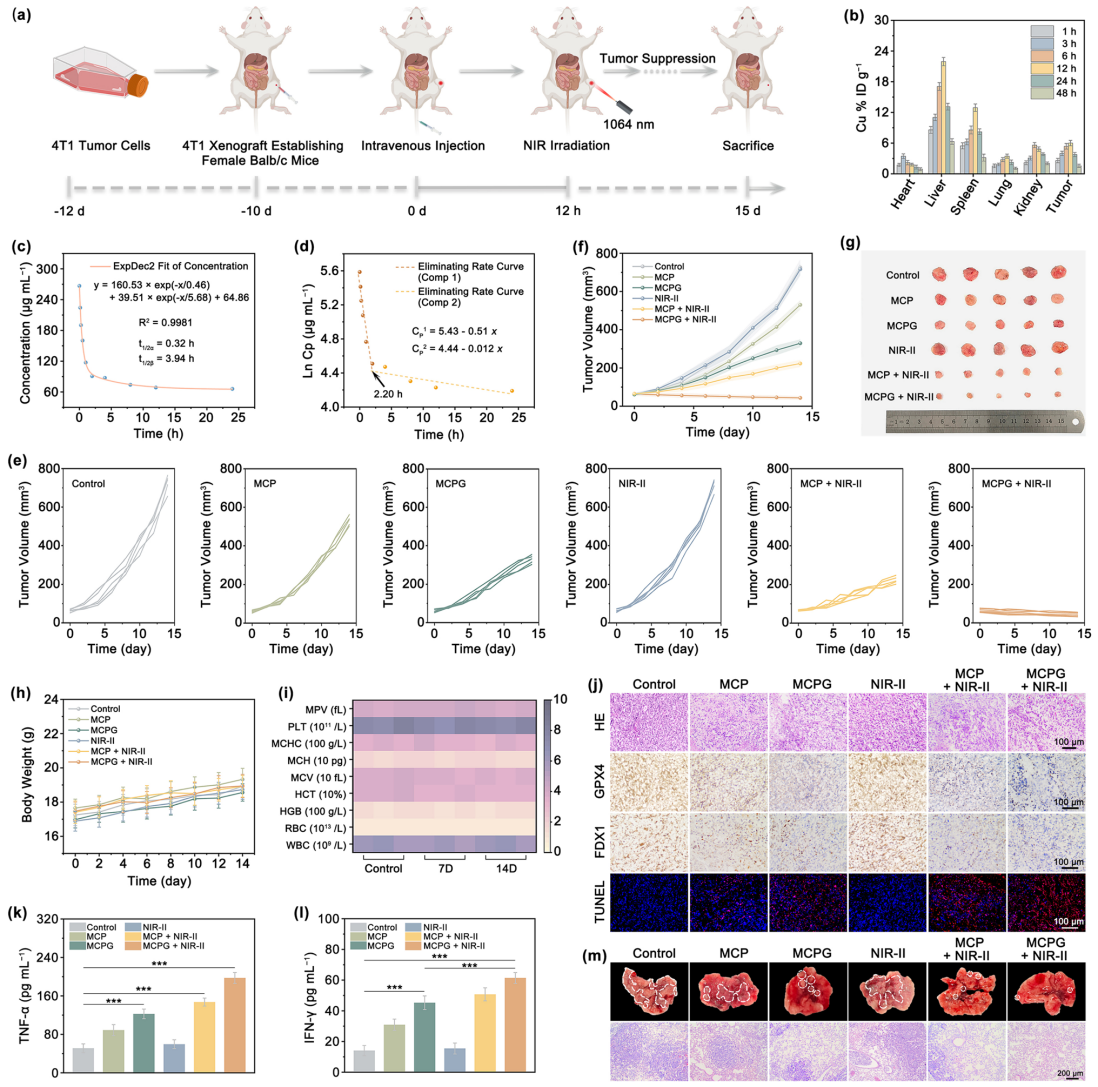
In vivo anticancer effect evaluation. **a** Schematic illustration of antitumor treatment of MCPG. **b** The biodistribution of Cu at various time intervals. **c** Blood circulation curve and **d** eliminating rate curve of MCPG. **e, f** Tumor volume change curves and **g** the photographs. **h** The average body weight. **i** Hematological indexes of mice after intravenous injection of MCPG after 7 and 14 days. **j** H&E, GPX4, FDX1, and TUNEL images of tumor tissues. Cytokines level of **k** TNF-α and **l** IFN- γ in plasma collected by ELISA. Data were presented as mean ± S.D. (*n* = 3). Statistical significance is evaluated by a two-tailed Student’s t test, **p < 0.01, ***p < 0.001. **m** Representative images of lung metastatic nodules and the H&E staining images

To evaluate the antitumor ability of MCPG nanosheets in vivo, we established a xenograft model using female BALB/c mice bearing 4T1 tumors (Fig. 7a). The hemolysis test indicated the excellent blood compatibility of MCPG (Fig. S47). Owing to capture by the reticuloendothelial system, elevated Cu levels were observed in both the liver and spleen. The tumor area exhibited a peak Cu concentration of 6.02% IDg^−1^ at 12-h post-injection, highlighting the accumulation of MCPG at tumor site (Fig. 7b). The blood circulation half-life was *t*_1/2_(α) = 0.32 h and *t*_1/2_(β) = 3.94 h, respectively (Fig. 7c). The elimination rate constant of MCPG increased from − 0.51 to − 0.012 μg mL^−1^ per hour with the shifting time of 2.20 h (Fig. 7d). In vivo metabolism behavior verified that MCPG was excreted from mice through feces and urine, thereby avoiding the long-term toxicity (Fig. S48). The mice were separated into six groups and treated with control, NIR-II, MCP, MCPG, MCP + NIR-II, or MCPG + NIR-II. Notably, the MCPG + NIR-II group exhibited the most potent suppression of tumor growth (Fig. 7e, f). The tumor suppression effect of each treatment group was further confirmed by the assessment of tumor weight on day 14 and the corresponding digital photographs (Figs. 7g and S49). Within the 14-day treatment period, the body weight of all groups exhibited a slight increase, signifying that the impact of MCPG on the overall health status of the mice was negligible (Fig. 7h). Hematological analyses of the mice treated at various time points also illustrated the favorable biocompatibility and biosafety of the MCPG nanosheets (Fig. 7i). Hematoxylin and eosin (H&E) staining of the major organs in each group indicated that MCPG had no notable side effects (Fig. S50). In tumor sections, the MCPG + NIR-II group presented the most pronounced tumor necrosis. Both the significant downregulation of FDX1 and GPX4 expression and the prominent red fluorescence signal in the TdT-mediated dUTP Nick End Labeling (TUNEL) assay confirmed the activation of the cuproptosis/ferroptosis/apoptosis pathways (Fig. 7j).

Induction of an anticancer immune response relies on the crucial effects of ICD [71–73]. When antigens encounter DCs, they are recognized and processed by immature DCs upon entry into the immune organs. Given that spleen is the principal immune organ, it plays a vital in both cellular and humoral immunity. The spleens of these mice were collected to prepare cell suspensions, and the levels of the co-stimulatory molecules CD80 and CD86 were measured using flow cytometry. Compared to the control group (6.14%), the highest DCs maturation in the MCPG + NIR-II group (15.6%) was ascribed to the greatest ICD (Fig. S51). The levels of relevant immunomodulatory cytokines, including TNF-α and IFN-γ, were acquired via an ELISA. The MCPG + NIR-II group exhibited the highest levels of cytokines, verifying the efficacy of the synergistic therapy in enhancing DCs maturation and promoting immune activation (Fig. 7k, l). After 21 days of treatment, lung metastasis was observed to validate the effect of the treatment. The control group displayed the most lung metastasis nodules, whereas the MCPG + NIR-II group presented the fewest nodules. Histological examination of representative lung tissues using H&E staining illustrated that the MCPG + NIR-II treatment group remarkably suppressed tumor metastasis (Fig. 7m). Conclusively, MCPG nanosheets with PTEC, photothermal-enhanced enzyme catalysis, and starvation synergistic therapy not only exhibited significant antitumor efficacy but also promoted an immune response to tumor metastasis.

## Conclusions

In summary, we synthesized biodegradable MCPG nanosheets rich in S_V_ for tumor treatment by disrupting intracellular redox homeostasis. MCPG triggers cuproptosis/ferroptosis/apoptosis and an antitumor immune response via PTEC, photothermal-enhanced enzyme catalysis, and starvation therapy. Encouragingly, MCPG with rich S_V_ displays an exceptional photothermal conversion efficiency (43.77%), leading to the local temperature rise. The resulting temperature gradient inside the material propels charge carrier diffusion from the hot side to the cold side, subsequently creating a potential difference and facilitating ROS production. By using POD-like and CAT-like activities, the reaction can generate ·OH and O_2_. The O_2_ generation can ameliorate tumor hypoxia, replenish depleted H_2_O_2_ by glycometabolism as well as amplify ROS generation, forming cascade catalytic reactions and establishing a positive feedback loop mechanism. Density functional theory calculations indicated that doping-induced vacancy defects remarkably facilitate the enzyme catalytic activity. Oxidative stress-promoted cuproptosis/ferroptosis/apoptosis is activated by heat, ROS, and Cu aggregation, disrupting mitochondrial metabolism and enhancing therapeutic effects. In addition, this system can stimulate ICD, thereby inducing an immune response and inhibiting tumor metastasis. This study introduces an unique research strategy for harnessing energy-converting nanomaterials in tumor therapy by triggering the cell death pathway. This research not only offers a strategy for imaging-guided tri-inducer of cuproptosis/ferroptosis/apoptosis, but also expands the scope and depth of copper-based thermoelectric materials in biomedical applications, thereby offering a promising development direction for the future advancement of cancer immunotherapy.

## Supplementary Information

Below is the link to the electronic supplementary material.

Supplementary file1 (DOCX 29263 KB)
