# Supplementary material for: Designing a Sulfur Vacancy Redox Disruptor for Photothermoelectric and Cascade-Catalytic-Driven Cuproptosis–Ferroptosis–Apoptosis Therapy
Source: Nanomicro Lett. 2025 Jul 4;17:321. doi: 10.1007/s40820-025-01828-8 (PMC12227396; doi:10.1007/s40820-025-01828-8)
Supplement: Supplementary file 1 — Supplementary file1 (DOCX 29263 KB) [file 40820_2025_1828_MOESM1_ESM.docx]

Supporting Information for

**Designing a Sulfur Vacancy Redox Disruptor for Photothermoelectric and Cascade-Catalytic-Driven Cuproptosis-Ferroptosis-Apoptosis Therapy**

Mengshu Xu^1, 2^, Jingwei Liu^2^, Lili Feng^1,^ *, Jiahe Hu^3^, Wei Guo^2,^ *, Huiming Lin^2^, Bin Liu^1^, Yanlin Zhu^1^, Shuyao Li^1^, Elyor Berdimurodov^4^, Avez Sharipov^5^, and Piaoping Yang^1,^ *

^1^Key Laboratory of Superlight Materials and Surface Technology, Ministry of Education, College of Materials Science and Chemical Engineering, Harbin Engineering University, Harbin 150001, P. R. China

^2^Key Laboratory of Photochemical Biomaterials and Energy Storage Materials, Heilongjiang Province and College of Chemistry and Chemical Engineering, Harbin Normal University, Harbin 150025, P. R. China

^3^Cancer Center, Department of Neurosurgery, Zhejiang Provincial People's Hospital, Affiliated People's Hospital, Zhejiang 310014, P. R. China

^4^Department of Physical Chemistry, National University of Uzbekistan, Tashkent 100034, Uzbekistan

^5^Faculty of Pharmacy, Department of Inorganic, Physical and Colloidal Chemistry, Tashkent Pharmaceutical Institute, Tashkent 100015, Uzbekistan

* Corresponding authors. E-mail: [fenglili@hrbeu.edu.cn](mailto:fenglili@hrbeu.edu.cn) (Lili Feng); [guowei@hrbnu.edu.cn](mailto:guowei@hrbnu.edu.cn) (Wei Guo); [yangpiaoping@hrbeu.edu.cn](mailto:yangpiaoping@hrbeu.edu.cn) (Piaoping Yang)

**S1 Experimental Sections**

**S1.1 Materials**

Copper(II) chloride (CuCl_2_·2H_2_O), manganese(II) chloride tetrahydrate (MnCl_2_·4H_2_O), glucose oxidase (GOx) and mPEG–COOH were purchased from Macklin (Shanghai, China). Ethylene glycol (EG) was obtained from Sinopharm (Beijing, China). 3,3′,5,5′-Tetramethylbenzidine (TMB), 5,5-dimethyl-1pyrroline N-oxide (DMPO), and anhydrous sodium sulfide (Na_2_S) were purchased from Aladdin Co. Ltd (Fukuoka, Japan). Fluorescein isothiocyanate (FITC), 2′,7′-dichlorofluorescein diacetate (DCFH-DA), Calcein-AM and propidium iodide (PI) were purchased from Shanghai Aladdin Biochemical Technology Co., Ltd (Shanghai, China). Phosphate buffered saline (PBS), trypsin-EDTA solution, and the JC-1 staining kit were obtained from Beyotime Inst. Biotech. (Haimen, China). Annexin V-FITC/PI apoptosis detection kit was purchased from Tianjin Sungene Biotech Co., Ltd. (Tianjin, China). All chemicals were used as received, without further treatment.

**S1.2 Experimental Apparatus**

The transmission electron microscopy (TEM) was performed using the FEI Tecnai G2 F20 microscope (Thermo Fisher Scientific, USA). The chemical valence was determined using X-ray photoelectron spectroscopy (XPS, ESCALAB 250XI, Thermo Fisher Scientific, USA). UV–vis spectra were recorded on a Shimadzu UV2550 spectrophotometer (China). Temperature changes in the tumor regions were recorded using an infrared camera (FLIR E6). The ESR spectra were obtained using a Bruker EMX1598 spectrometer (USA). MTT assay was performed using a microplate reader (Infinite M200, Tecan, Männedorf, Switzerland). The QT-GX-1550 laser (Kongtum) was used to produce 1064 nm light during the *in vitro* and *in vivo* treatment process.

**S2 Supplementary Table and Figures**

**Table S1** The comparison of maximum reaction velocity (*V*_max_) and Michaelis-Menten constant (*K*_m_) of different nanozymes

| **Nanozyme** | **Substrate** | ***V*_max_**  **[× 10^−8^ M s^−1^]** | ***K*_m_**  **[mM]** |
| --- | --- | --- | --- |
| rFeO_x_-HMSN [S1] | H_2_O_2_ | 1.43 | 29.57 |
| BiFeO_3_ [S2] | H_2_O_2_ | 1.24 | 12.45 |
| Fe/Cu-BBDC MOF [S3] | H_2_O_2_ | 1.43 | 0.07 |
| Cu_2_O/Au-Pt@MOF@F127 [S4] | H_2_O_2_ | 4.05 | 22.40 |
| Bi_2_CuO_4_ [S5] | H_2_O_2_ | 2.56 | 47.2 |
| Bi_2_CuO_4_-V_Cu_ [S5] | H_2_O_2_ | 2.82 | 40.36 |
| Pd [S6] | H_2_O_2_ | 3.01 | 132.31 |
| PdCu_0.8_ [S6] | H_2_O_2_ | 3.42 | 81.12 |
| PdCu_1.2_ [S6] | H_2_O_2_ | 3.63 | 76.12 |
| CeO_2_ [S7] | H_2_O_2_ | 2.45 | 40.34 |
| PEG/Ce-Bi@DMSN [S8] | H_2_O_2_ | 2.63 | 32.11 |
| PEG-ZIF-8:Mn/Au [S9] | H_2_O_2_ | 2.63 | 5.55 |
| UCNPs-MOF[PCN-224(Fe)] [S10] | H_2_O_2_ | 2.31 | 35.41 |
| CuS-PEG/GOx (This work) | H_2_O_2_ | 2.38 | 25.56 |
| Cu_2_MnS_3-x_-PEG/GOx (MCPG)  (This work) | H_2_O_2_ | 4.83 | 21.69 |

**
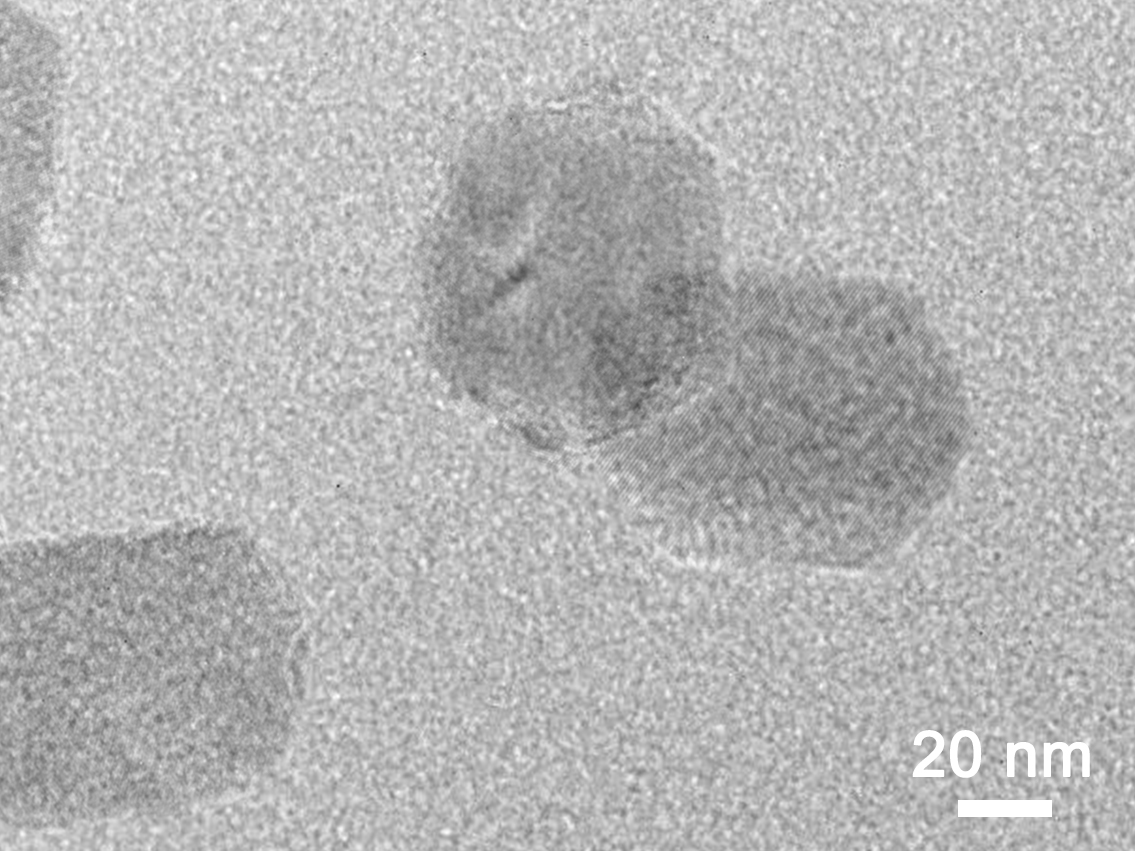
**

**Fig. S1** TEM image of MCPG


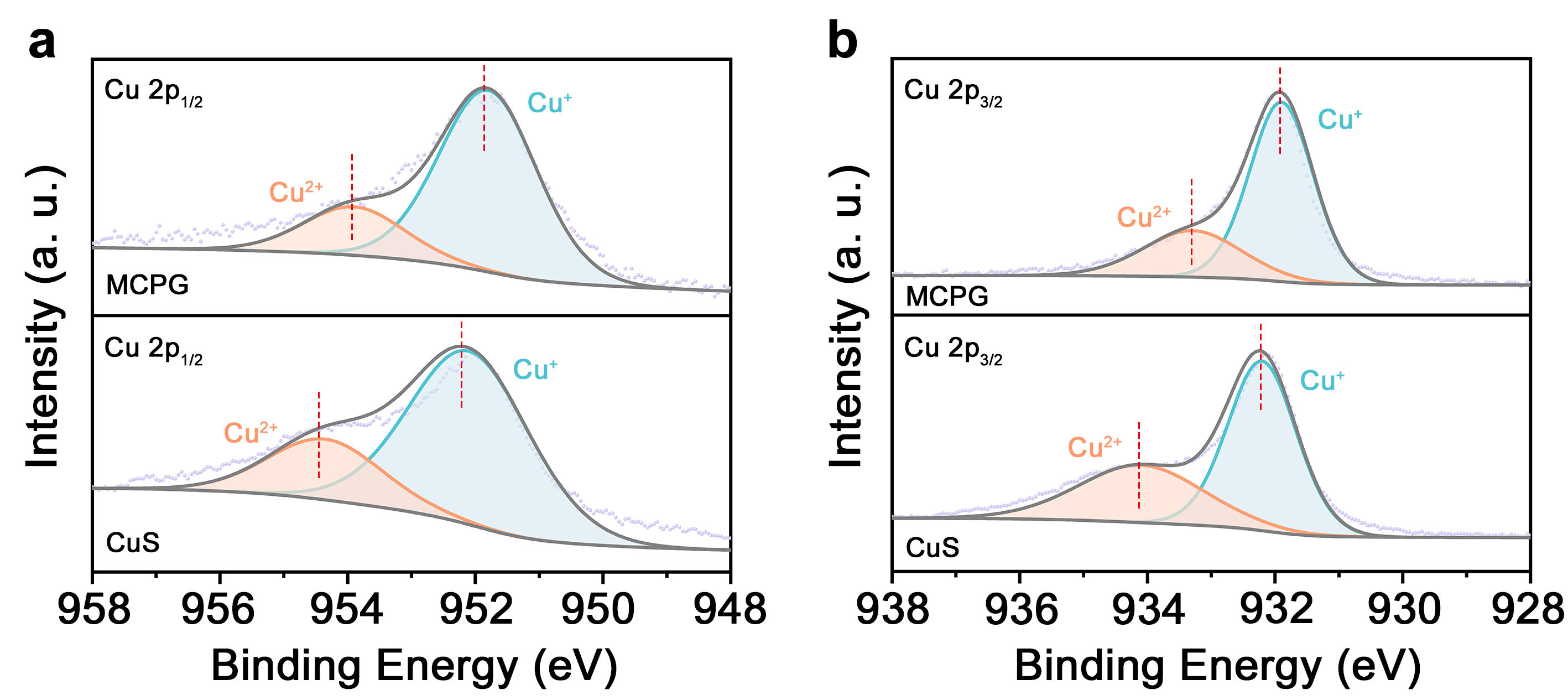


**Fig. S2** The partially amplified detail of Cu 2p XPS high-resolution spectra for MCPG


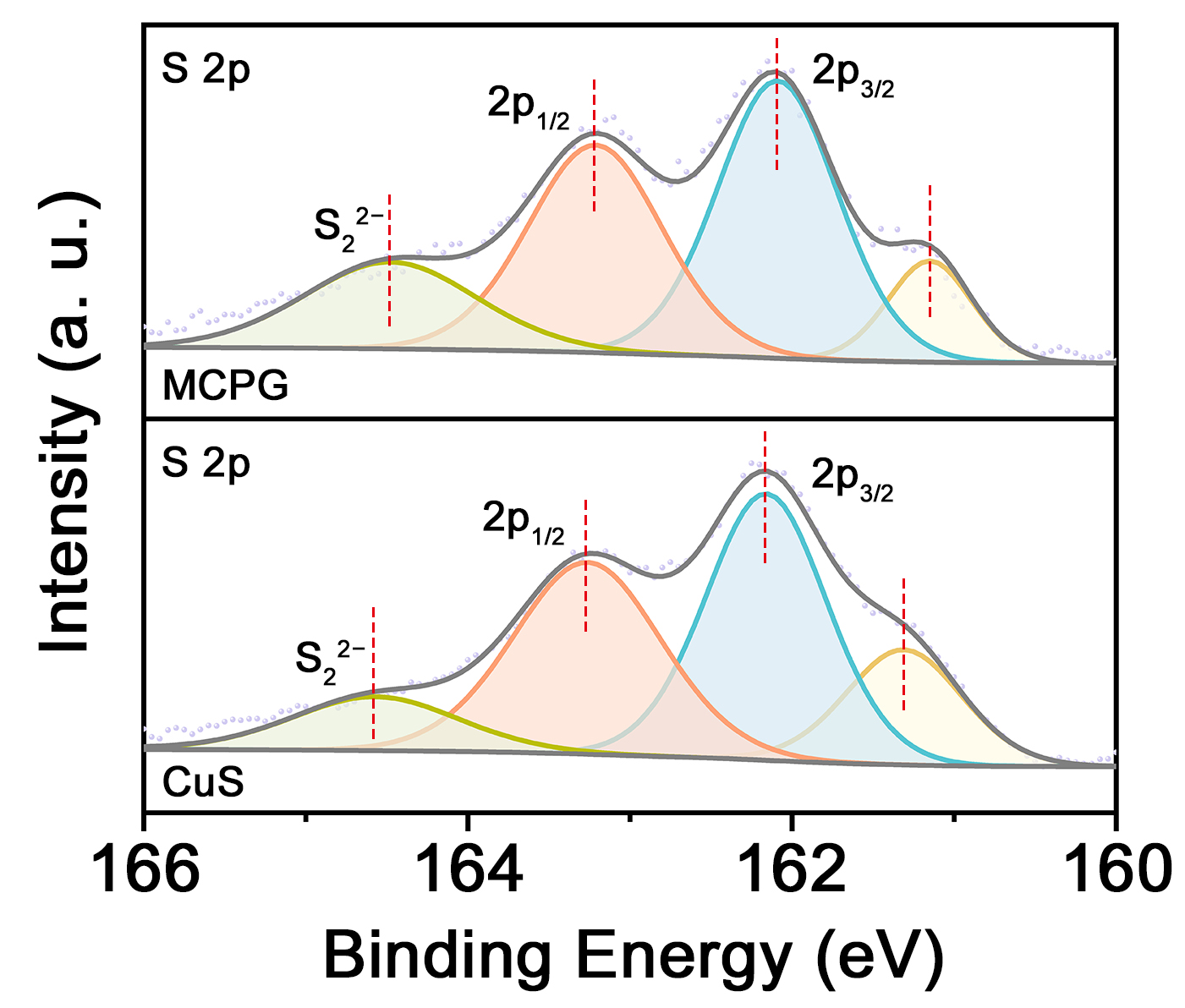


**Fig. S3** The partially amplified detail of S 2p XPS high-resolution spectra for MCPG


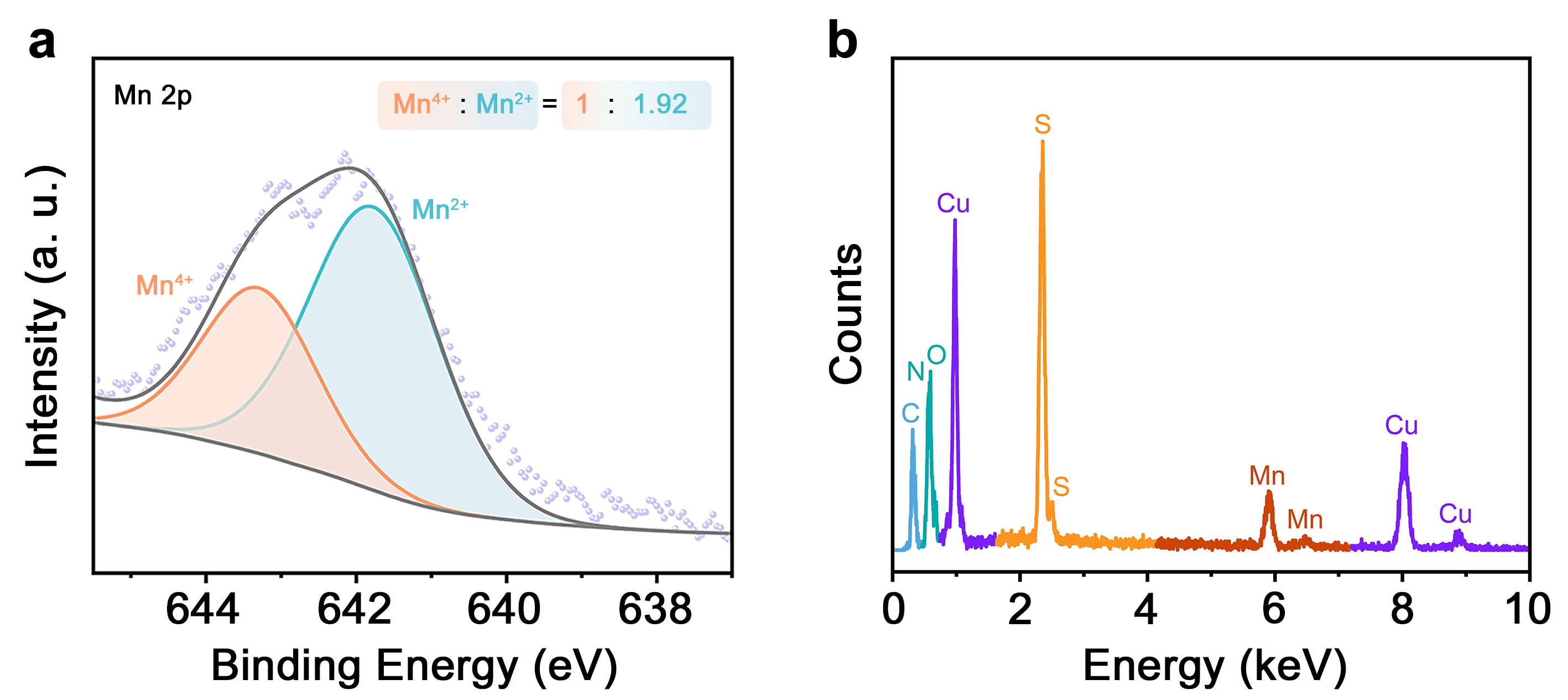


**Fig. S4** **a** XPS high-resolution spectrum of Mn 2p and **b** EDS spectrum for MCPG


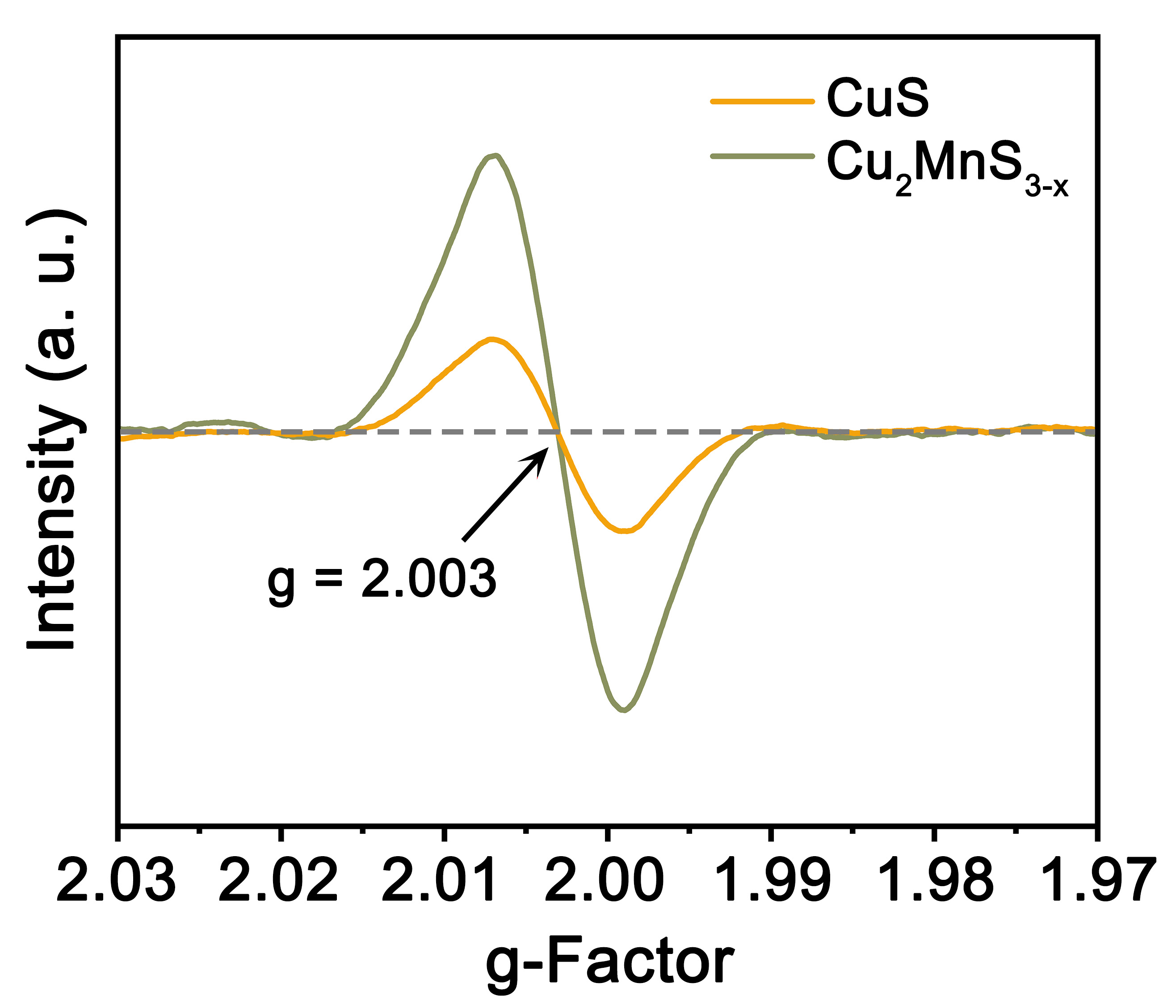


**Fig. S5** EPR spectra of CuS and Cu_2_MnS_3-x_


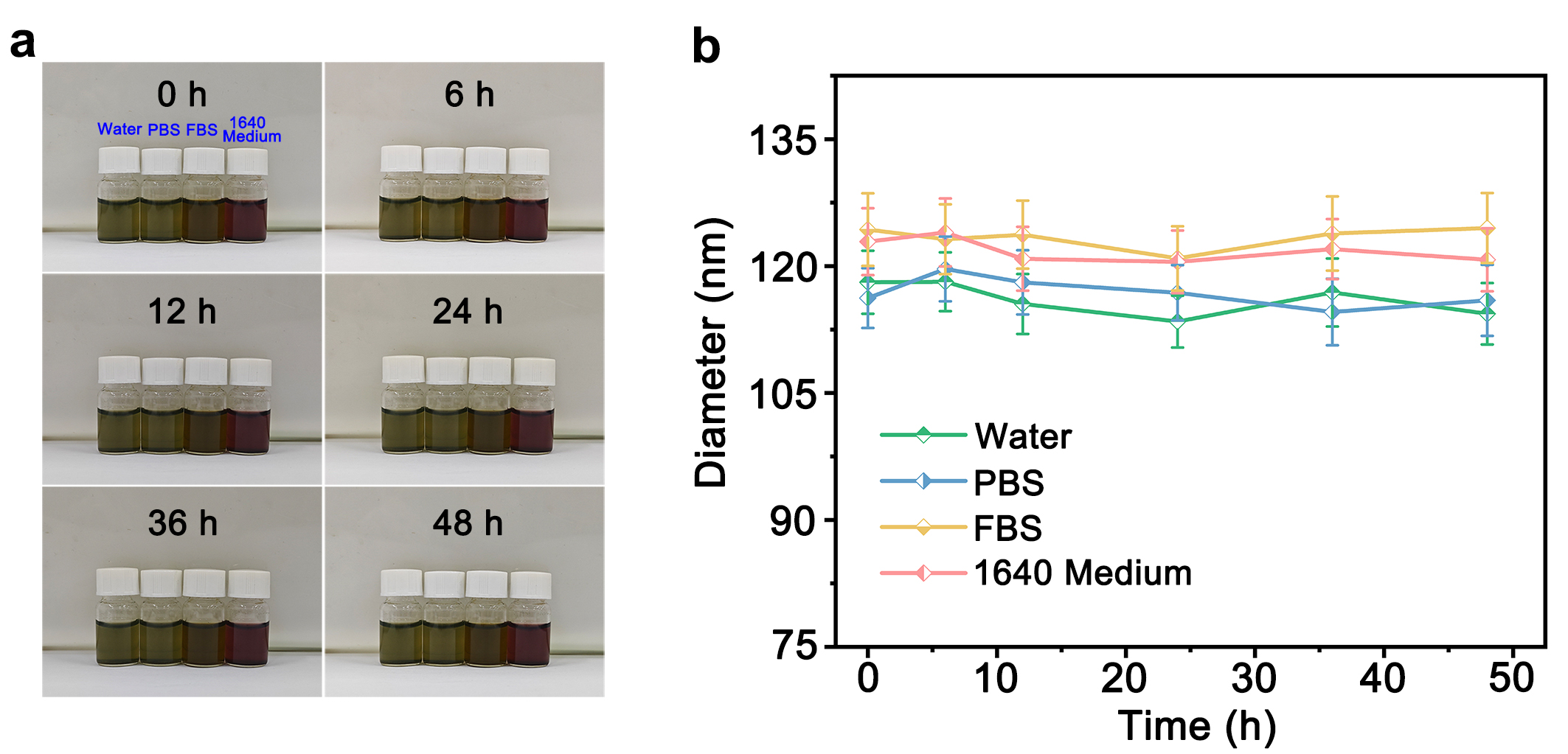


**Fig. S6 a** Photographs and **b** hydrodynamic dimension changes of MCPG dispersed in water, PBS, FBS, and 1640 culture medium at various treatment periods

**
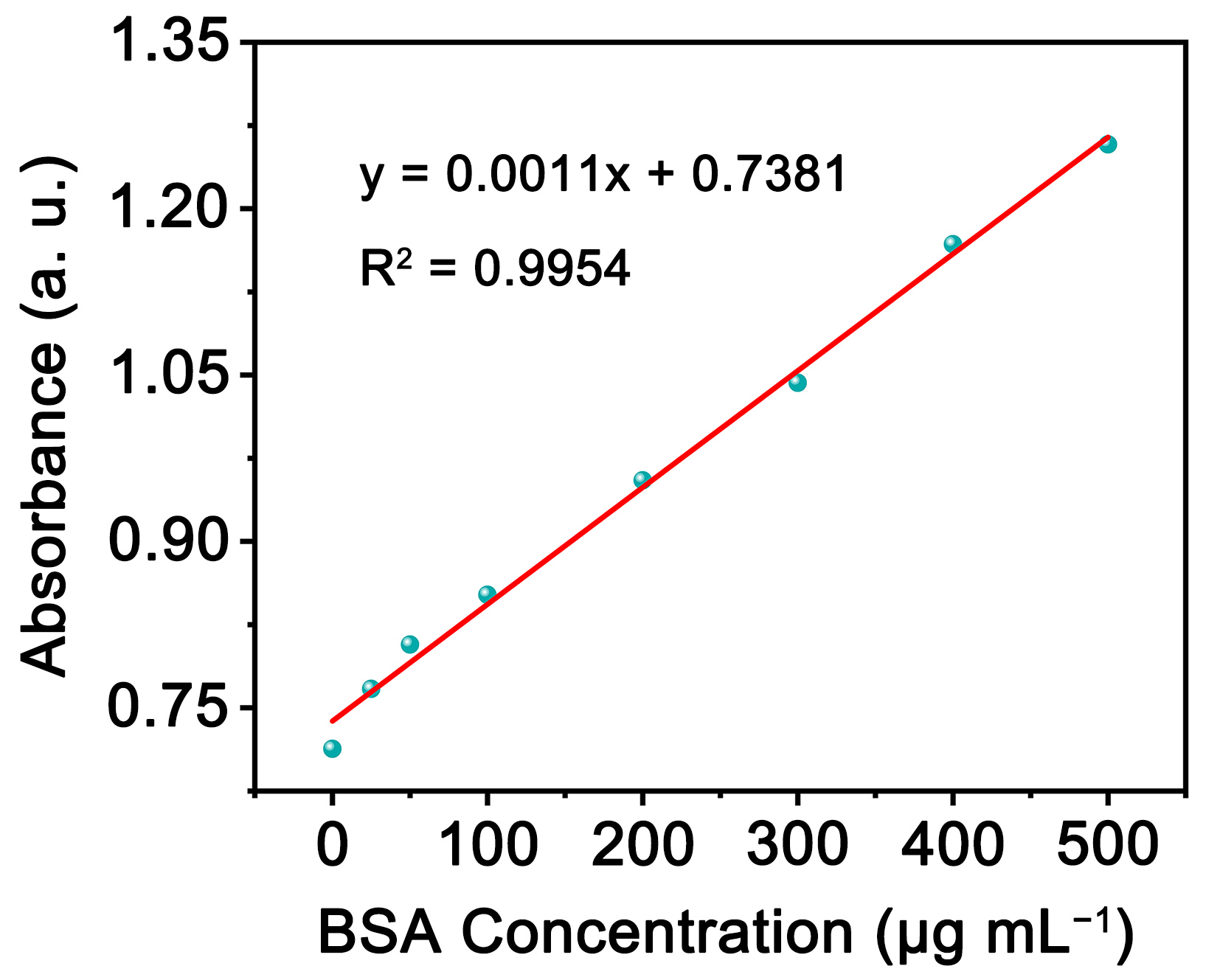
**

**Fig. S7** The standard curve of GOx at different concentrations

$\text{wt}\text{\%=}\frac{\text{m}_{\text{0}}\text{-}\text{m}_{\text{r}}}{\text{m}_{\text{s}}\text{+}\text{m}_{\text{0}}\text{-}\text{m}_{\text{r}}}$ (S1)

where *m_0_* refers to the initial mass of GOx, *m_s_* is attributed to the mass of the MCP sample, and *m_r_* represents the residual mass of GOx in the supernatant.

**
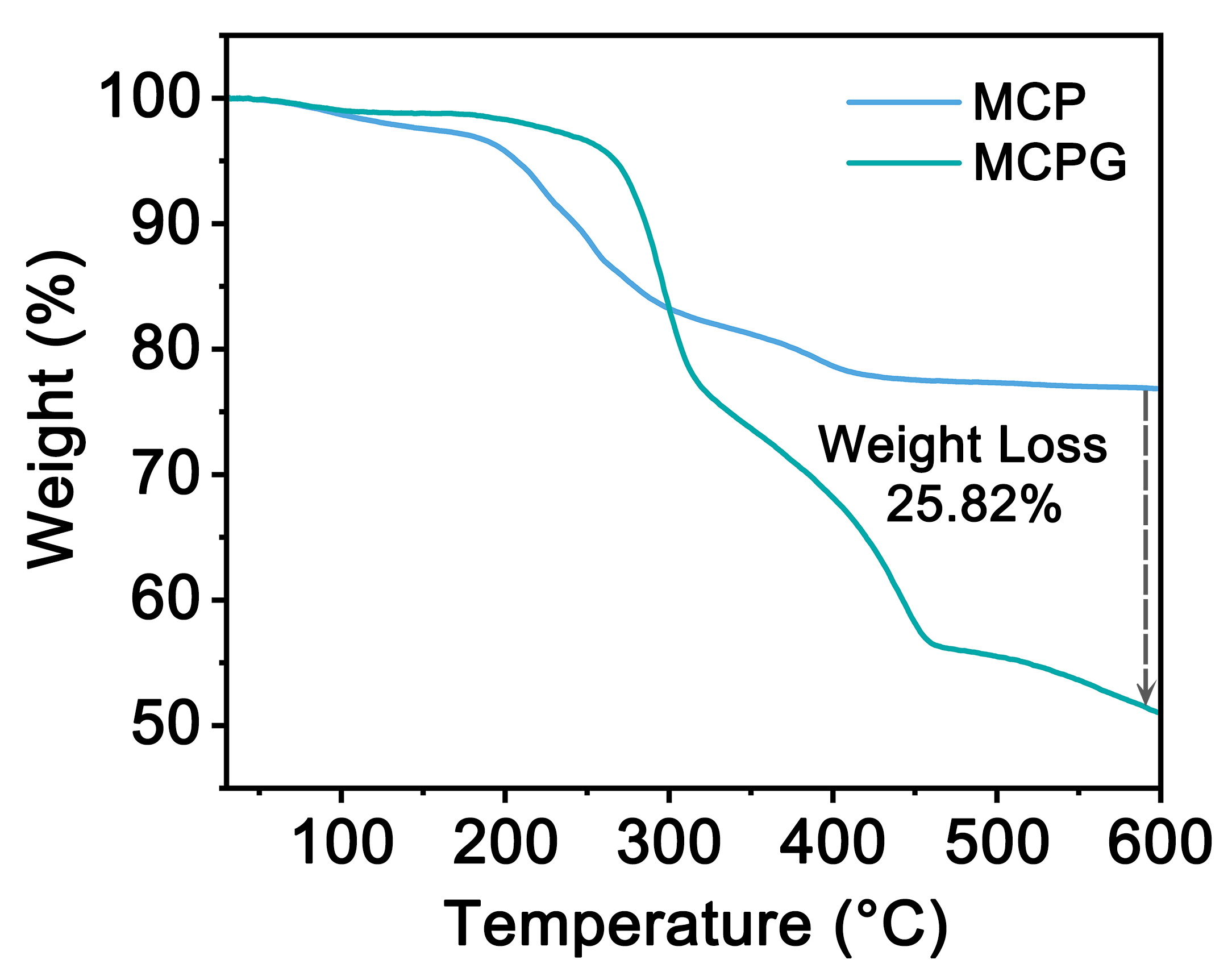
**

**Fig. S8** TGA curves of MCP and MCPG


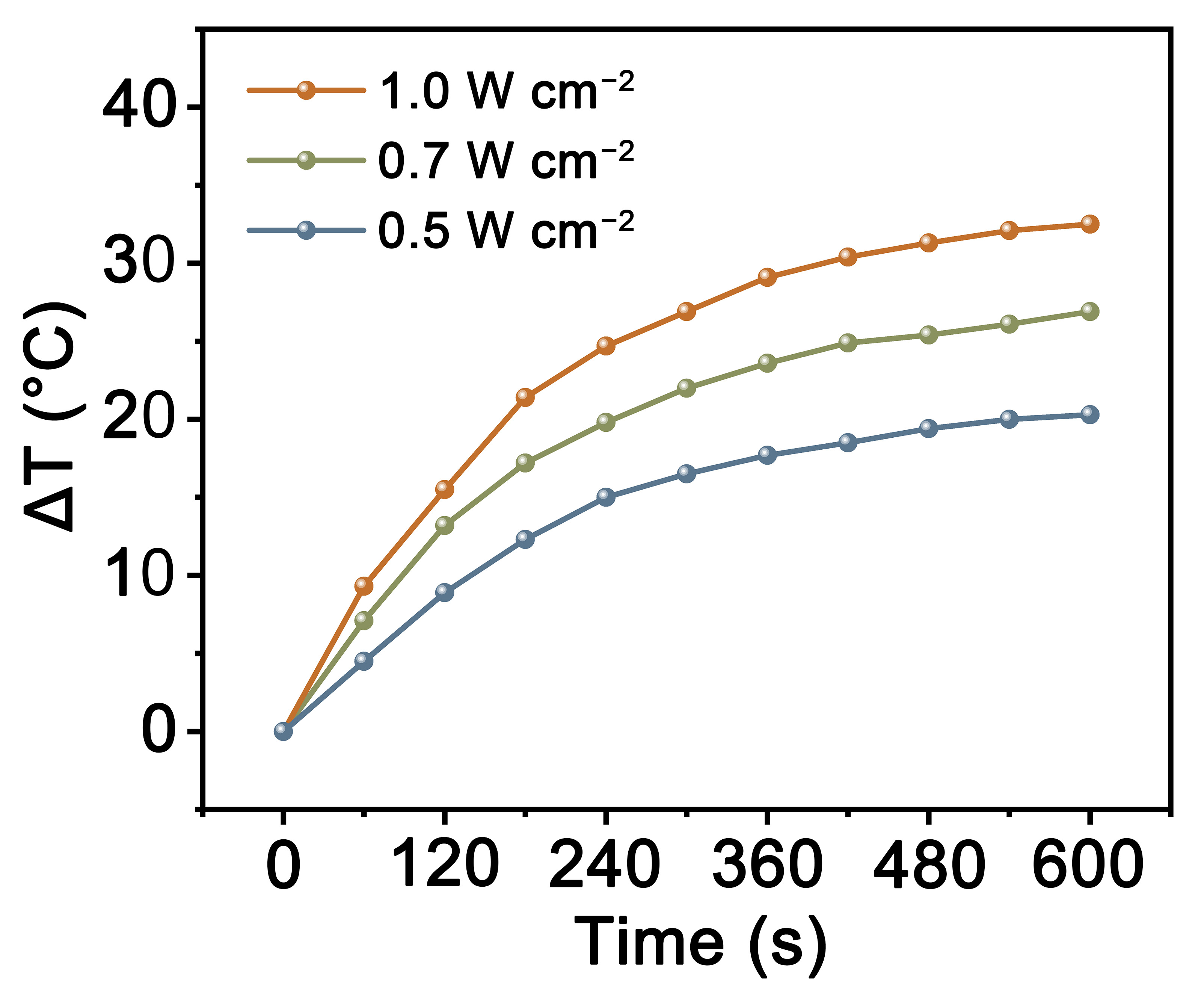


**Fig. S9** Temperature change curves of dispersed MCPG (200 μg mL^−1^) under 1064 nm laser irradiation at various power densities

**
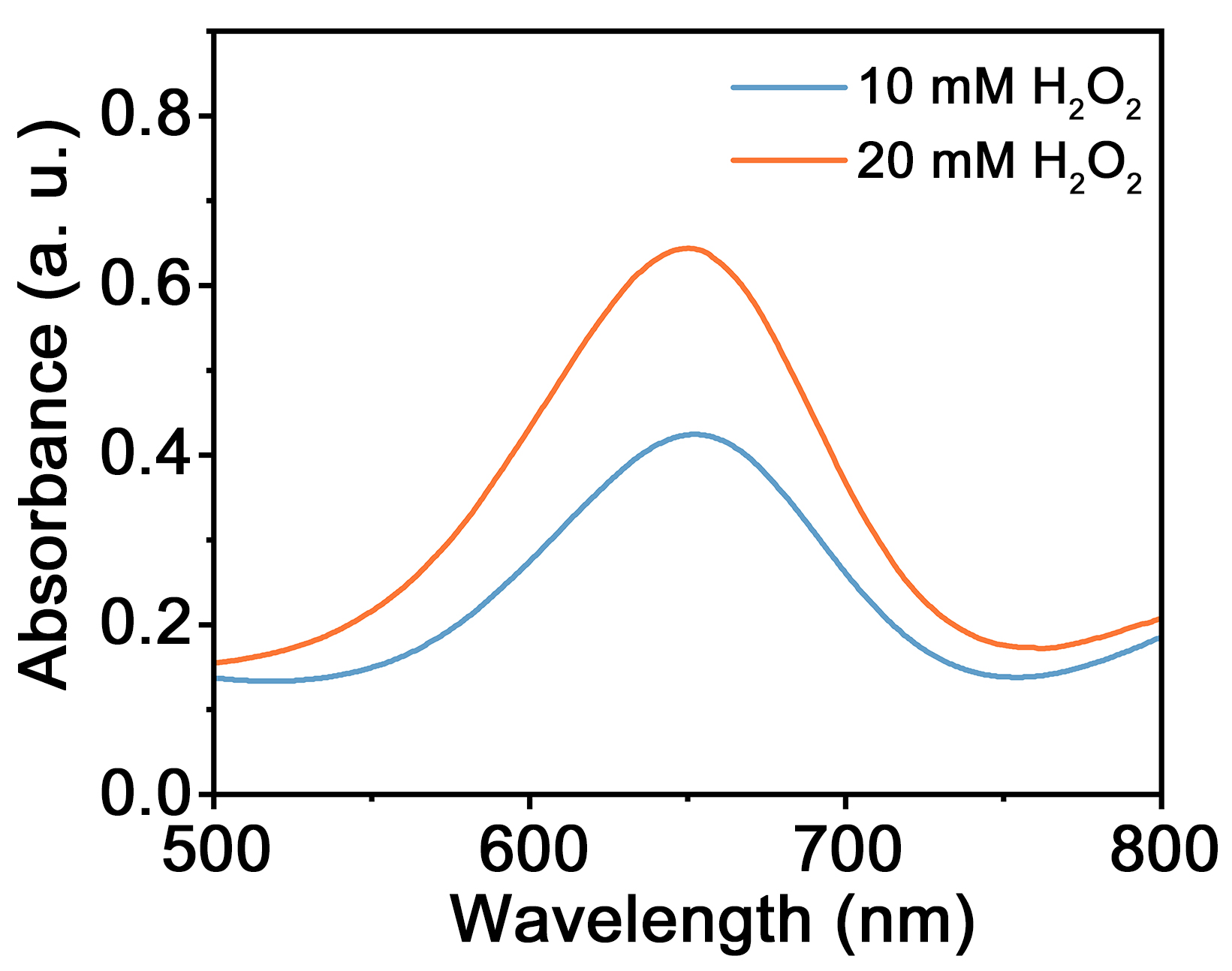
**

**Fig. S10** UV–vis spectra of the catalytic oxTMB by adding various concentrations of H_2_O_2_

**
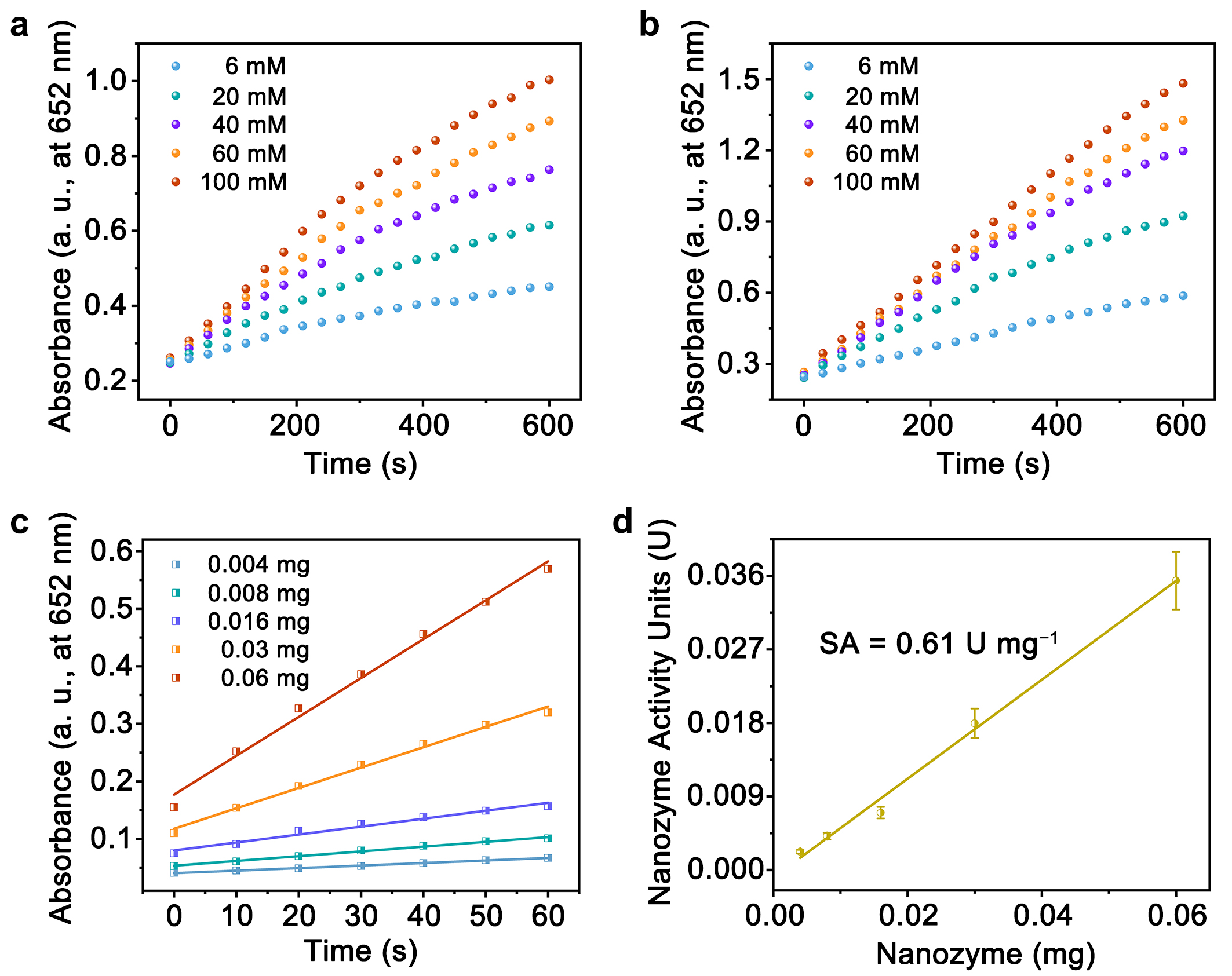
**

**Fig. S11** The absorbance change of oxTMB catalyzed by MCPG with various concentrations of H_2_O_2_ at **a** 25 °C and **b** 50 °C, respectively. **c** The initial linear curves of oxTMB with MCPG addition at different concentrations *versus* reaction time. **d** Specific nanoenzyme activity of MCPG


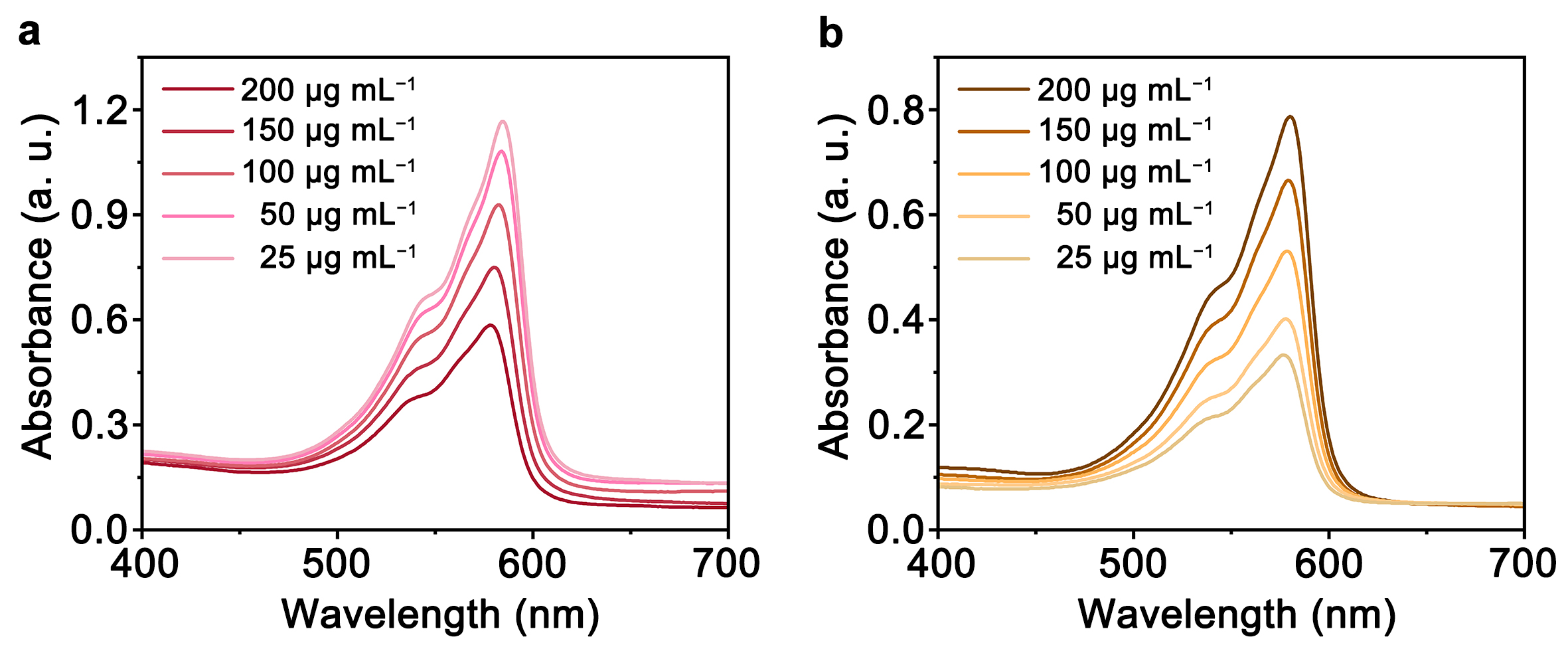


**Fig. S12** The concentration-dependent H_2_O_2_ consumption or generation of MCPG nanozymes in the presence of **a** H_2_O_2_ and **b** glucose


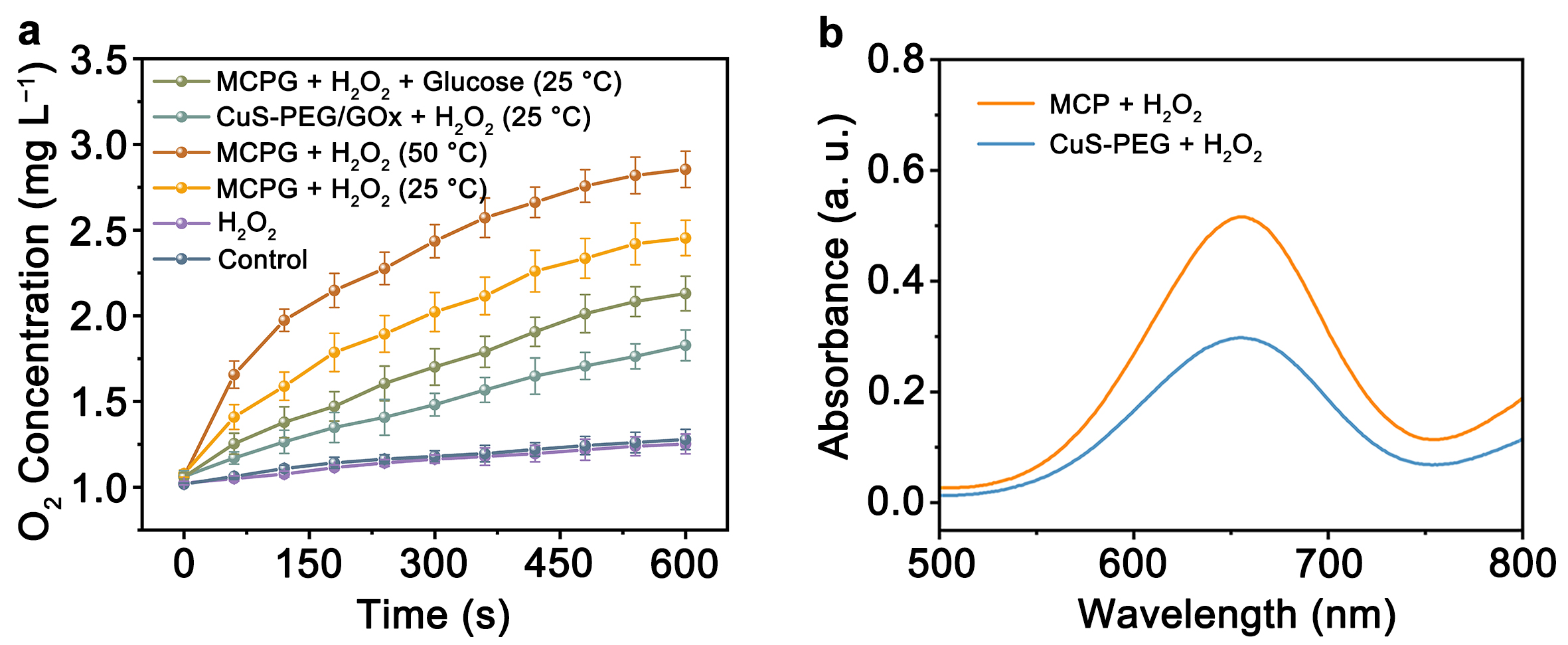


**Fig. S13 a** The detection of O_2_ concentration after various treatments. **b** UV–vis absorption spectra of oxTMB treated by CuS-PEG and MCP with H_2_O_2_


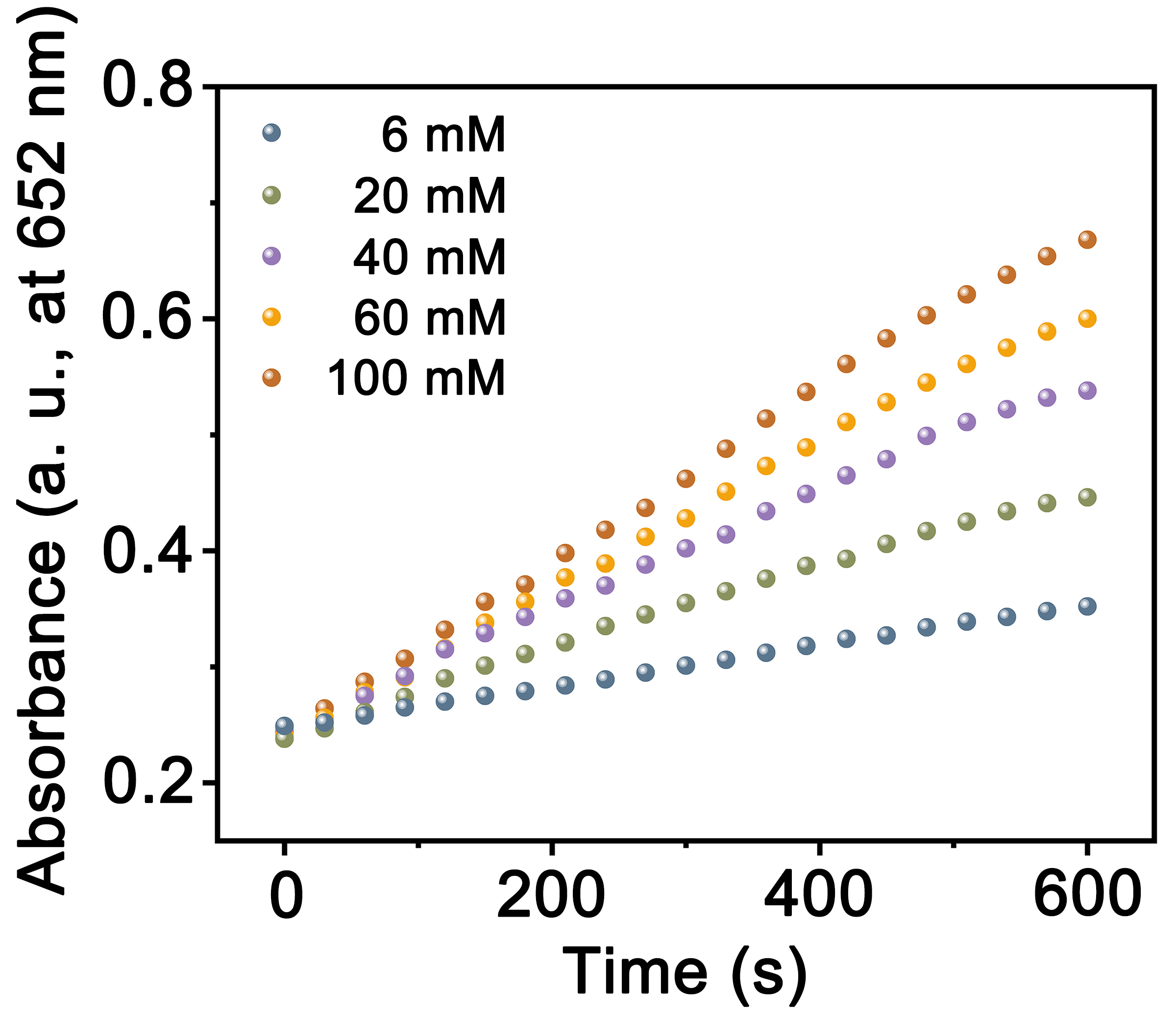


**Fig. S14** The absorbance change of oxTMB catalyzed by CuS-PEG/GOx with various concentrations of H_2_O_2_


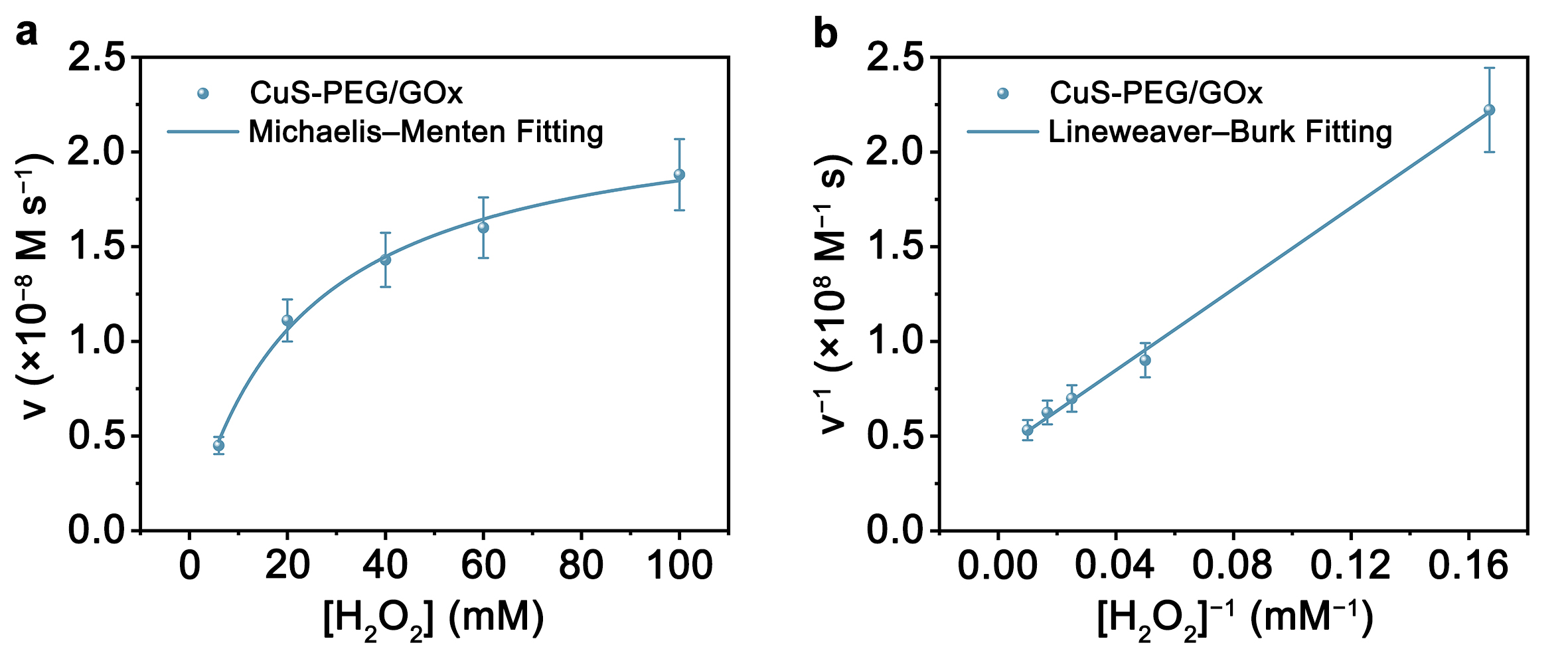


**Fig. S15 a** Michaelis–Menten curves and **b** Lineweaver–Burk plotting of CuS-PEG/GOx


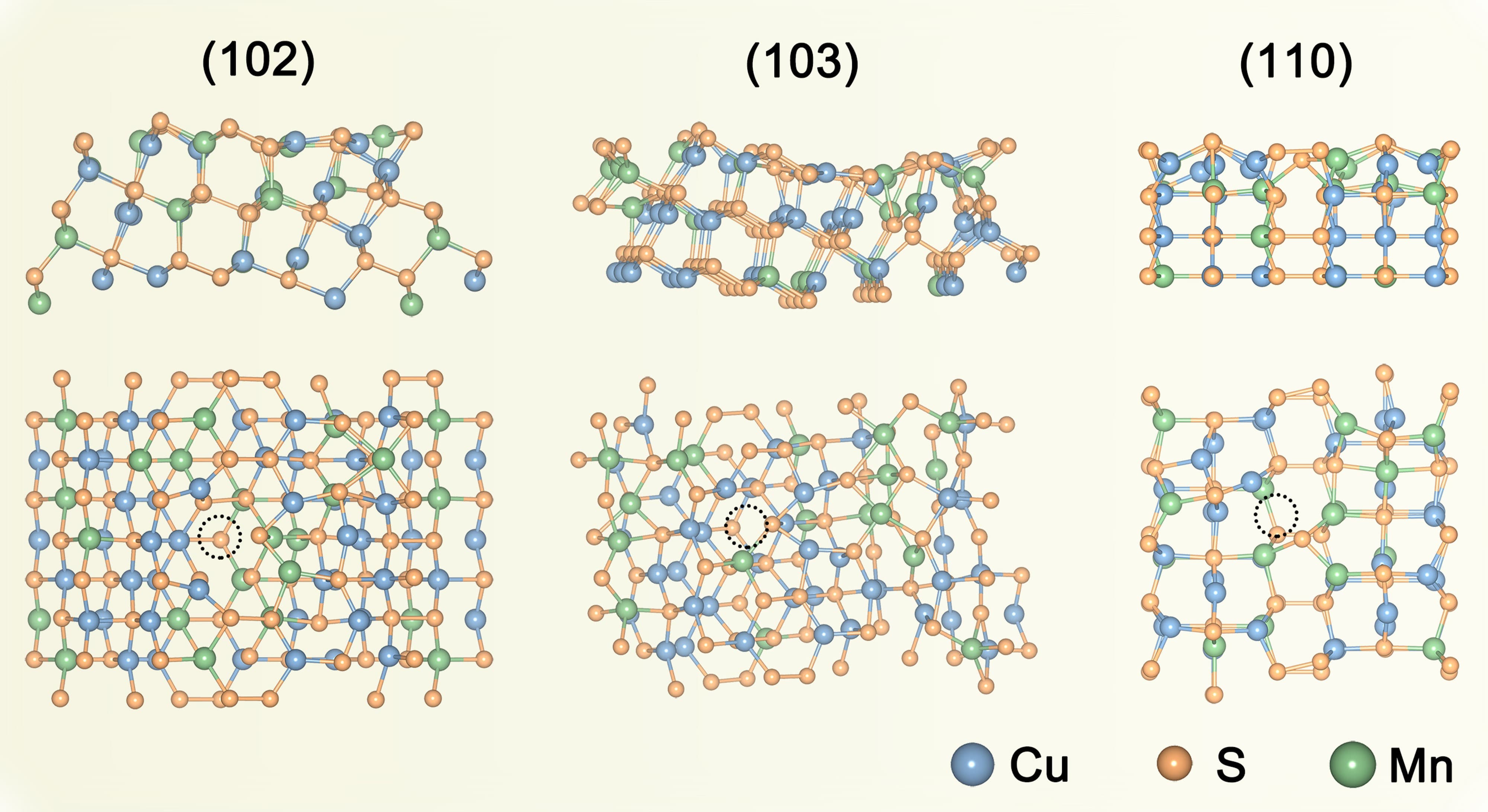


**Fig. S16** Optimized structural models of Cu_2_MnS_3-x_ with different crystal facets


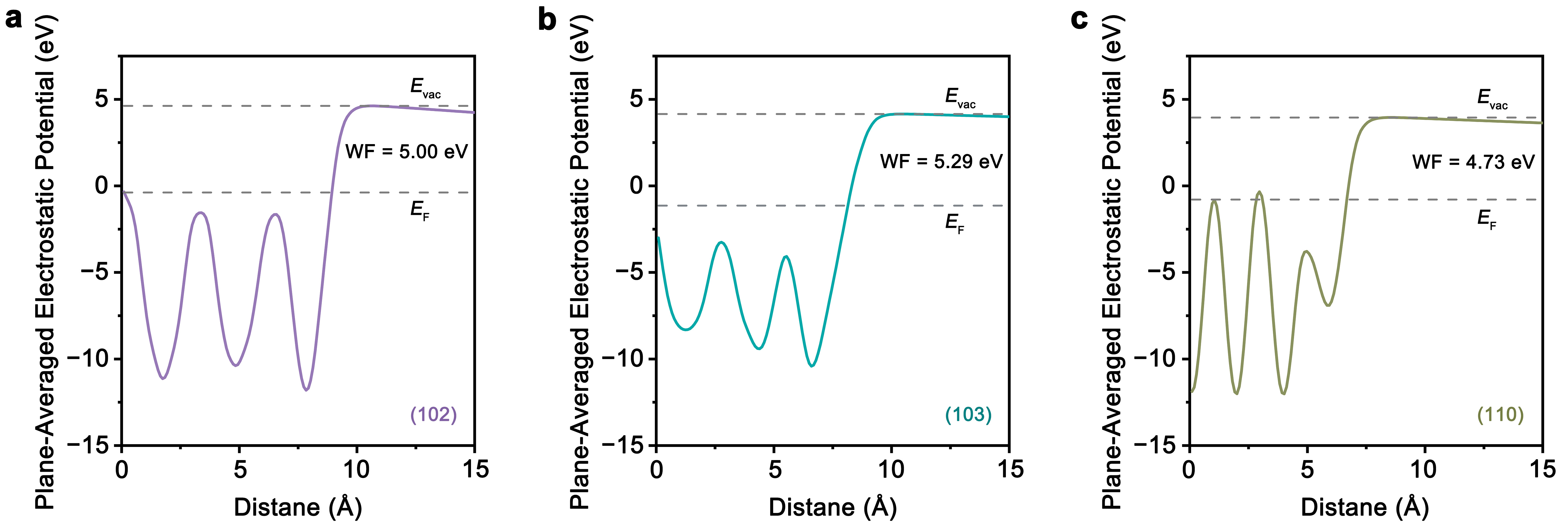


**Fig. S17** Calculated plane-averaged electrostatic potential of Cu_2_MnS_3-x_ with different crystal facets


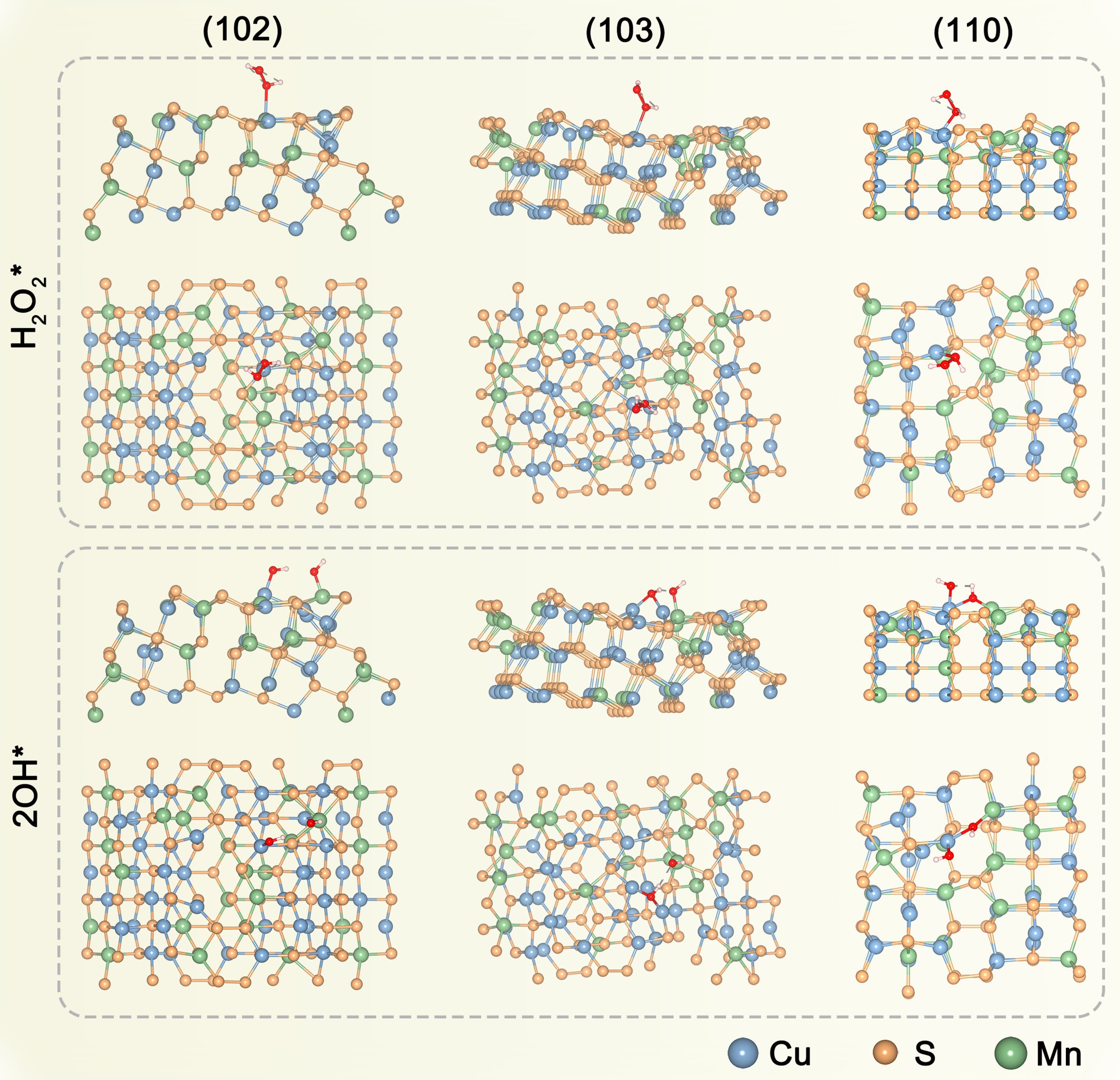


**Fig. S18** The surface structure under the initial state (IS, H_2_O_2_) and final state (FS, 2OH*) of Cu_2_MnS_3-x_ with different crystal facets during the POD-like catalysis process


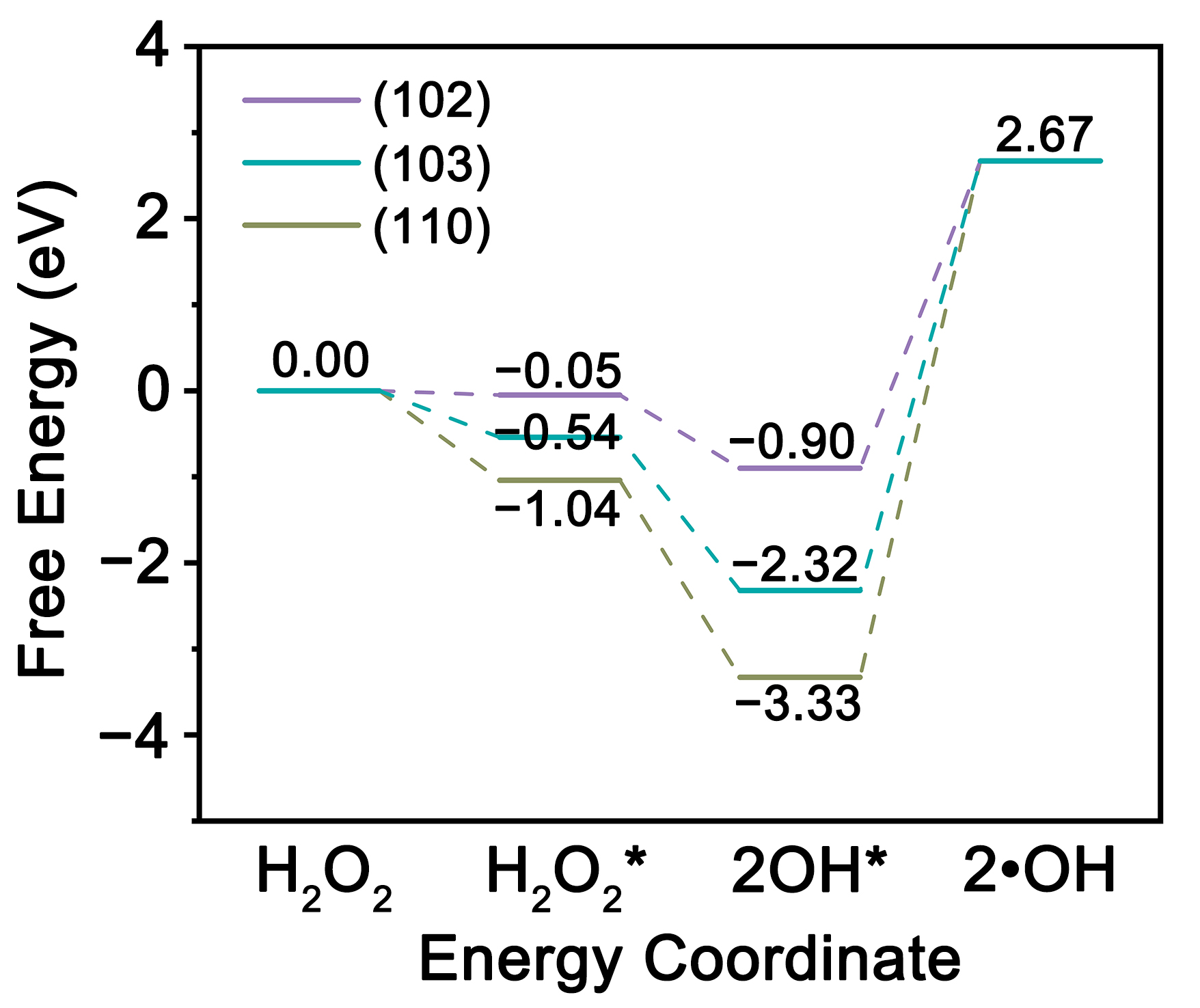


**Fig. S19** Energy charts of the POD-like catalysis pathways of Cu_2_MnS_3-x_ with different crystal facets


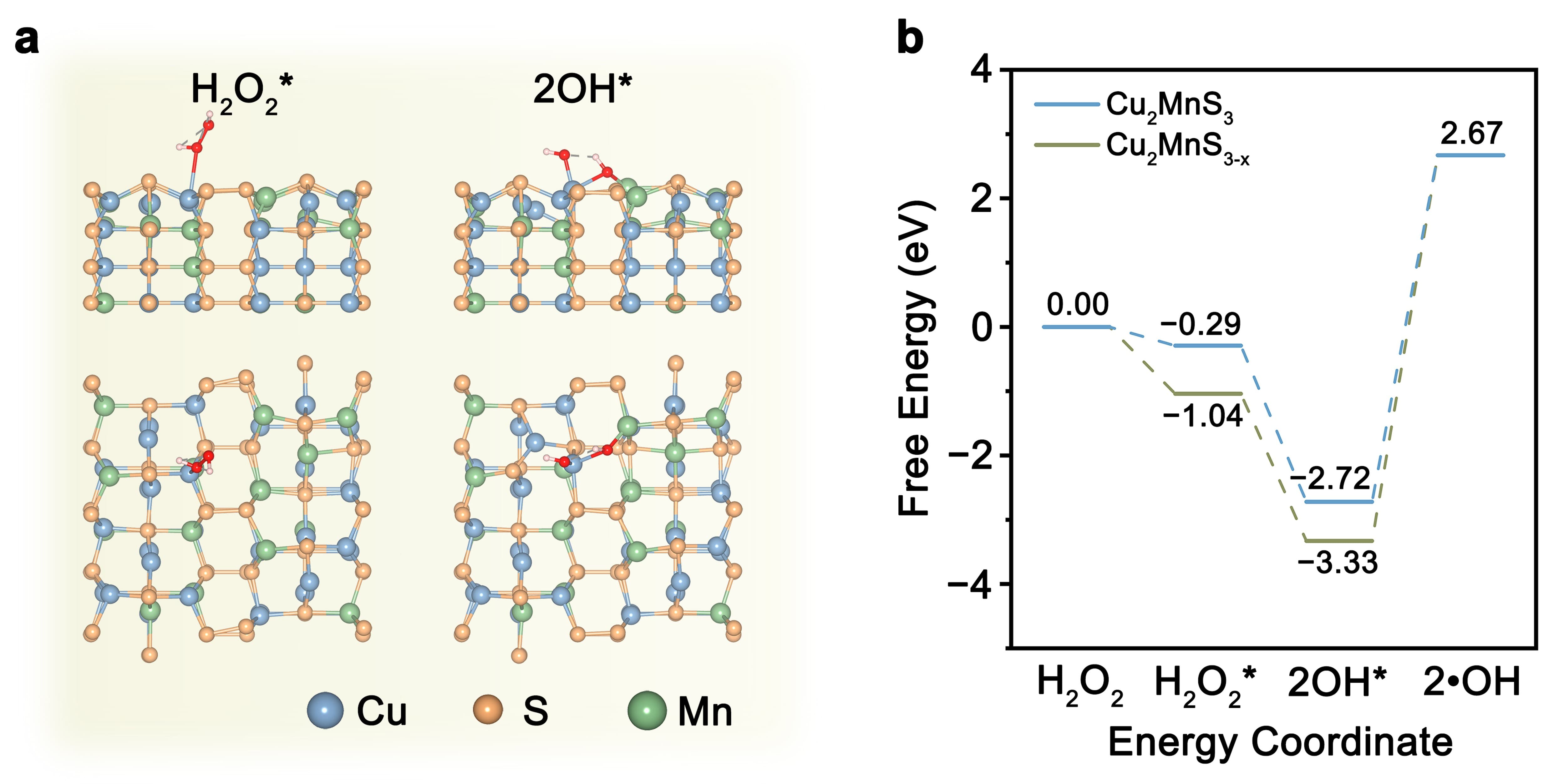


**Fig. S20 a** The surface structure under the initial state (IS, H_2_O_2_) and final state (FS, 2OH*) of Cu_2_MnS_3_ with (110) crystal facet during the POD-like catalysis process. **b** Energy charts of the POD-like catalysis pathways of Cu_2_MnS_3_ with (110) crystal facet


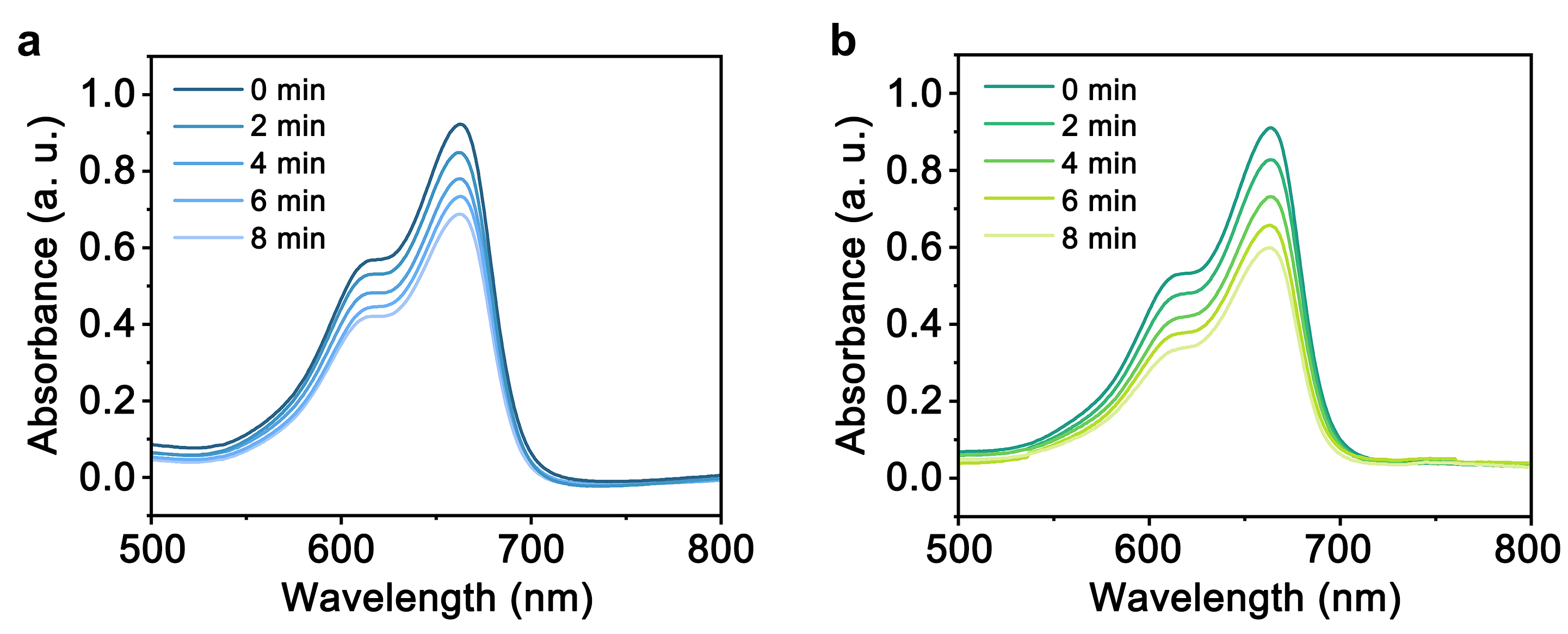


**Fig. S21** The repercussion of glucose **a** omission or **b** addition on the degradation of MB


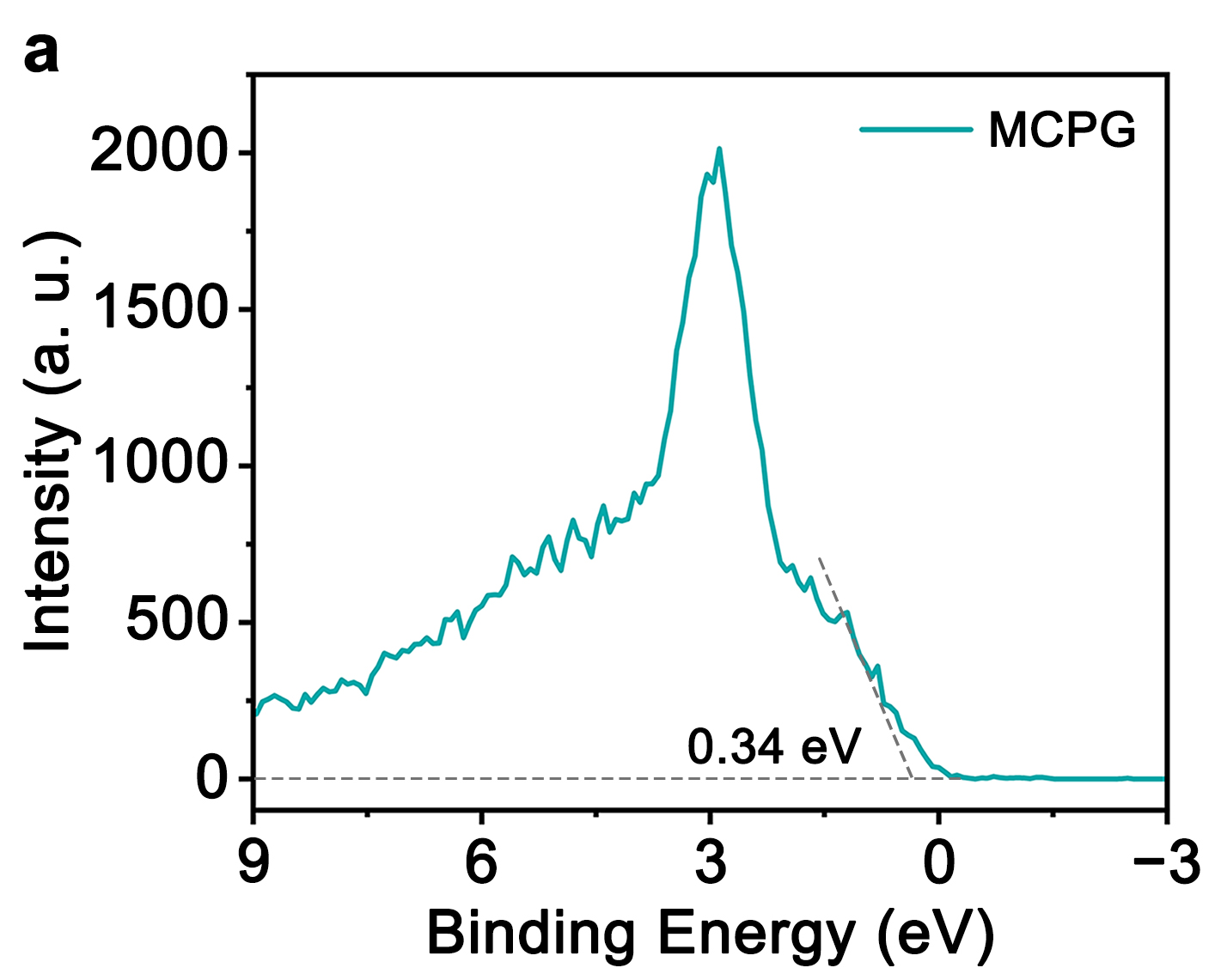


**Fig. S22** XPS valence band spectrum of MCPG


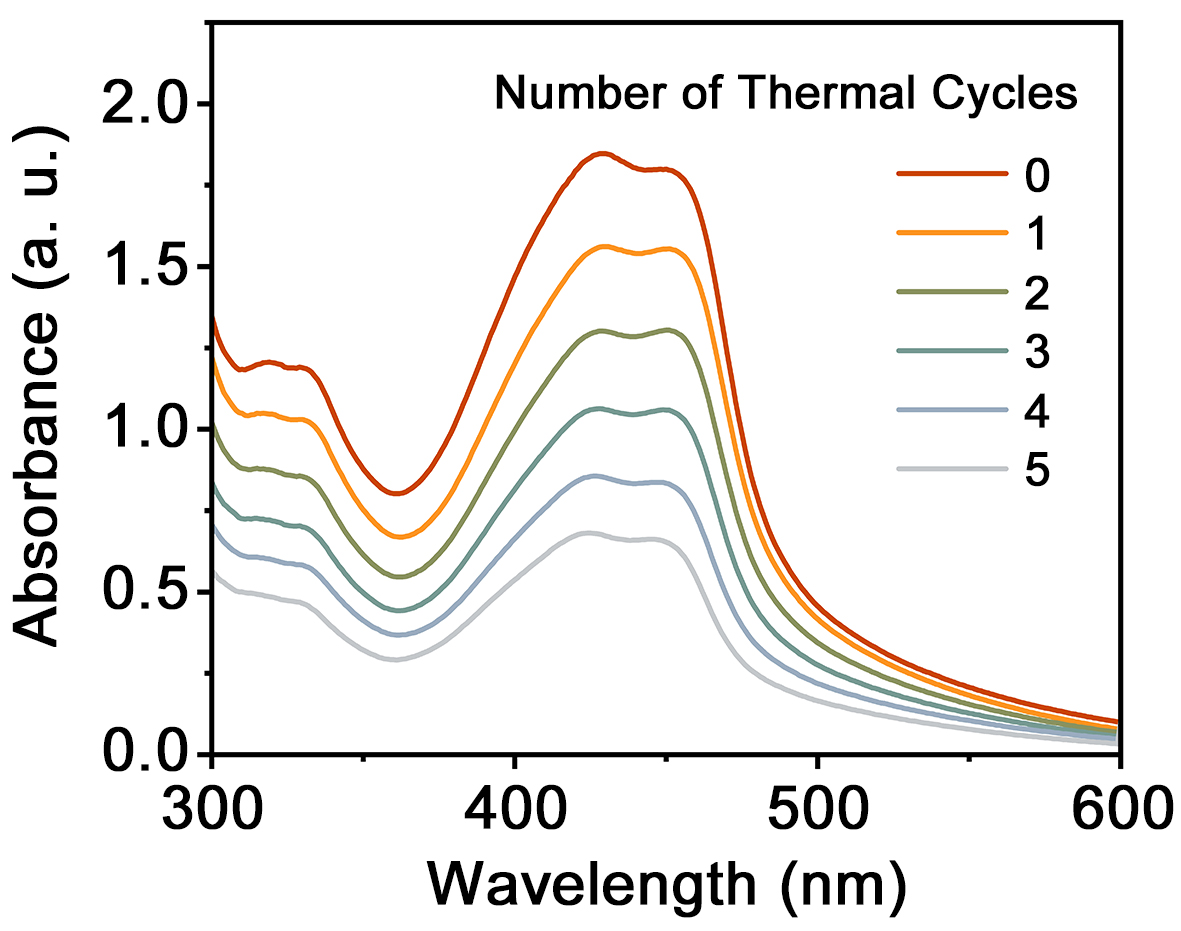


**Fig. S23** UV−vis absorption spectra of DPBF in MCPG solution *versus* temperature differences by hot/cold water bath cycles


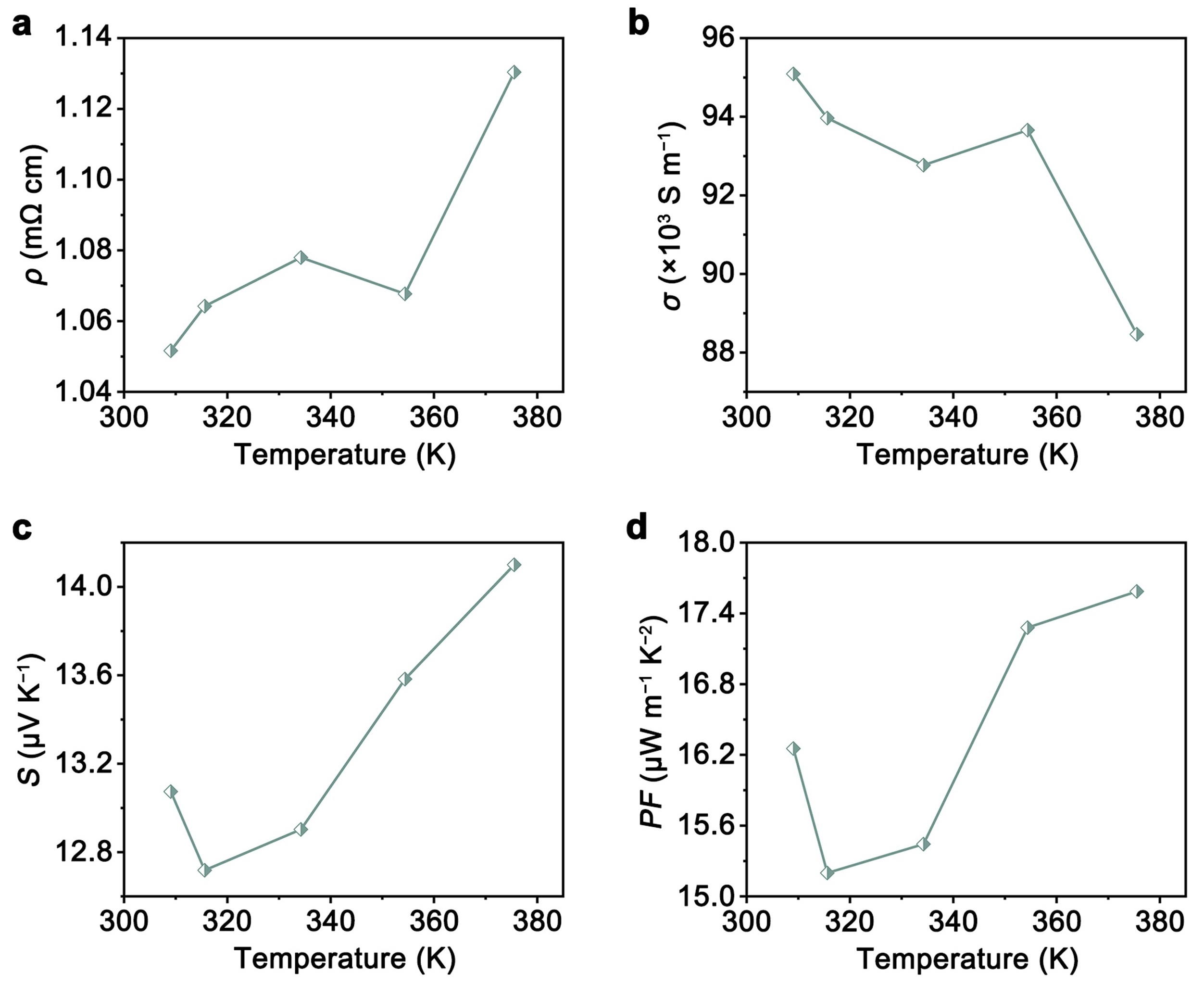


**Fig. S24** Electrical performance of MCP. **a** Electrical resistivity *ρ*, **b** electrical conductivity *σ*, **c** Seebeck coefficient *S*, and **d** power factor *PF*


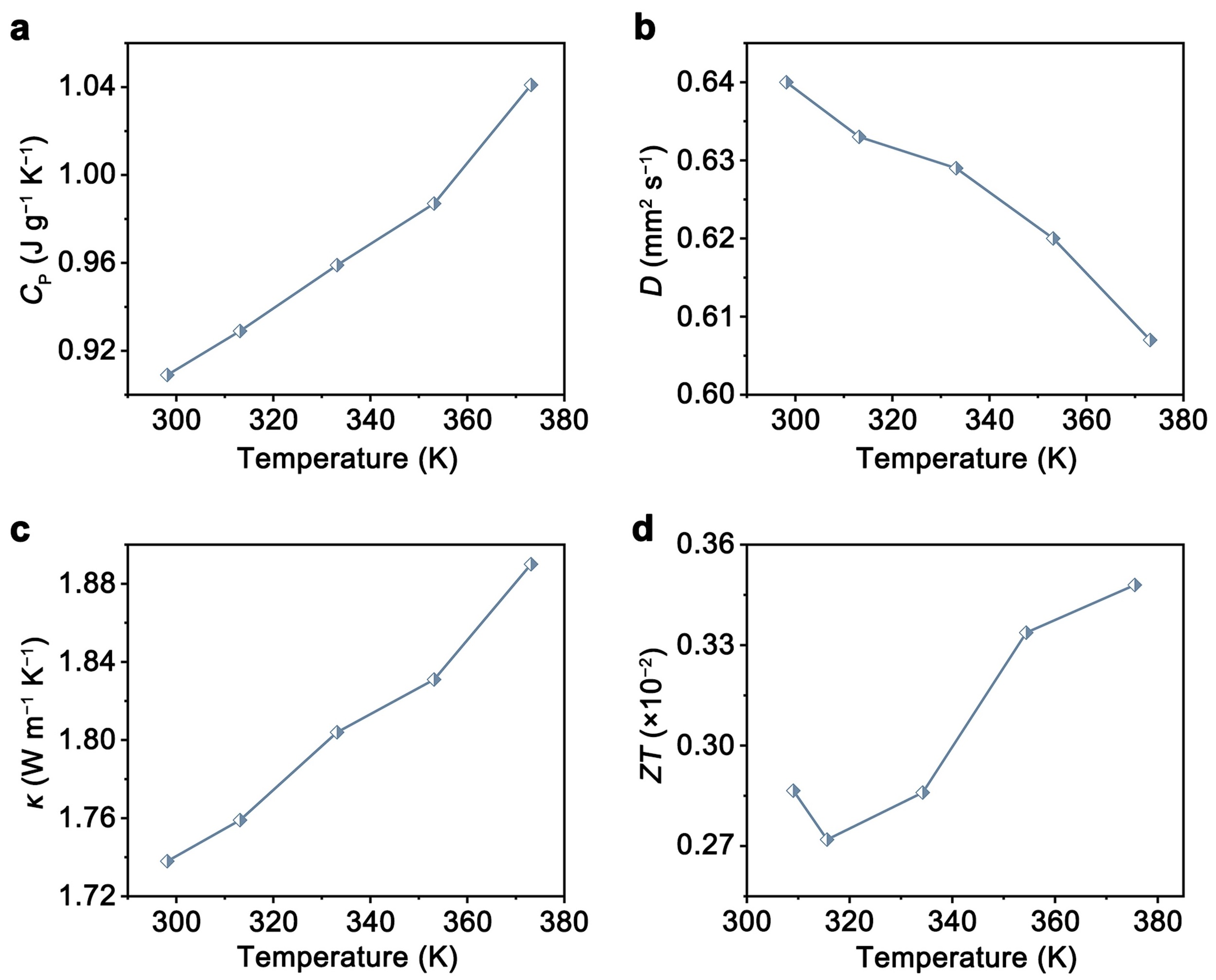


**Fig. S25** Thermoelectric performance of MCP. **a** Specific heat capacity *C_P_*, **b** thermal diffusivity *D*, **c** thermal conductivity *κ*, and **d** dimensionless figure of merit *ZT* protection material during thermal runaway


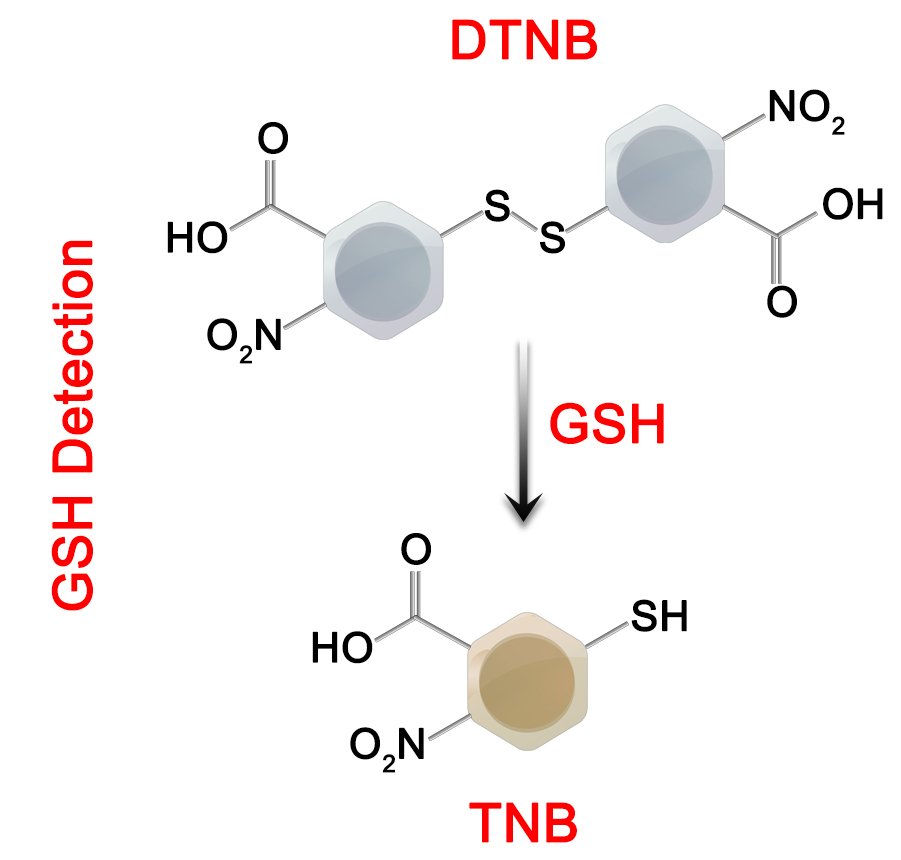


**Fig. S26** Diagram of colorless DTNB reacting with GSH to obtain yellow products


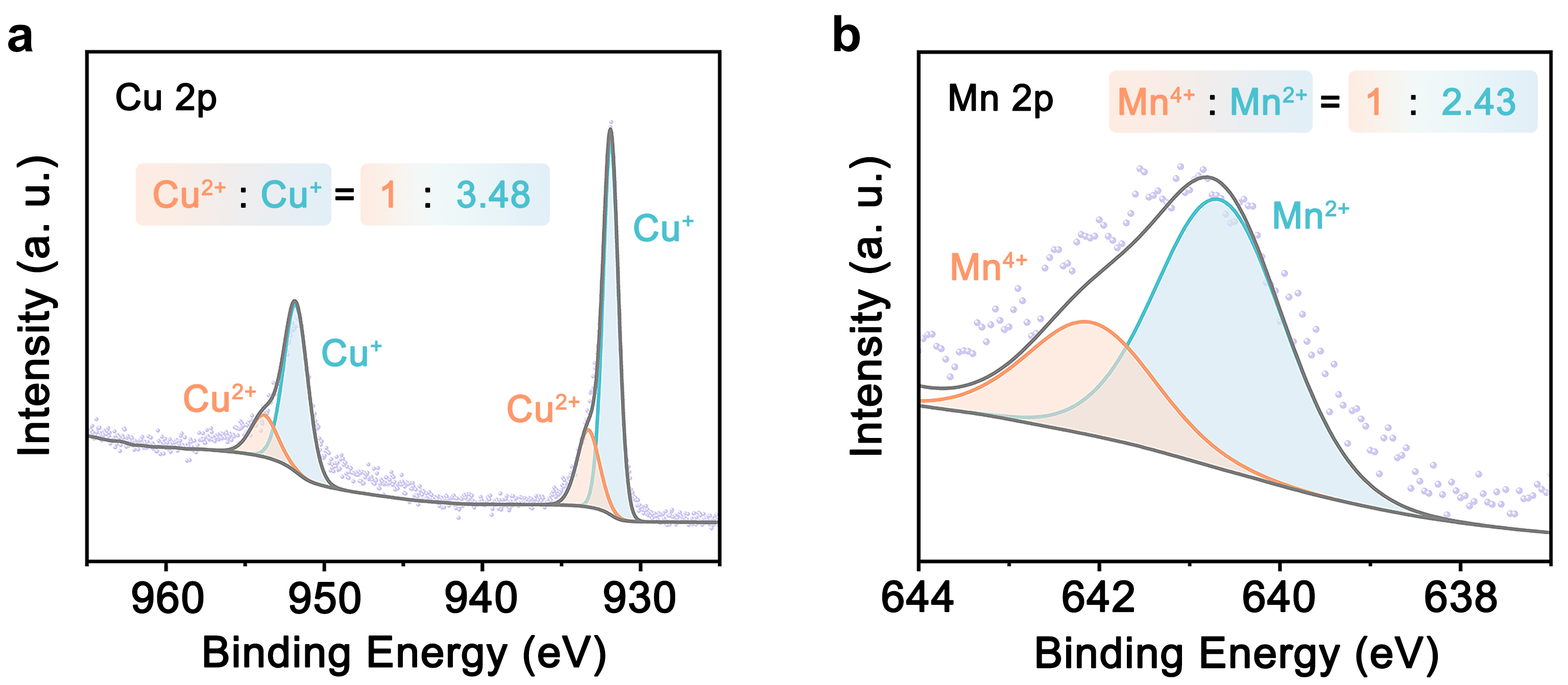


**Fig. S27** High-resolution XPS spectra of **a** Cu 2p and **b** Mn 2p for MCPG with GSH treatment


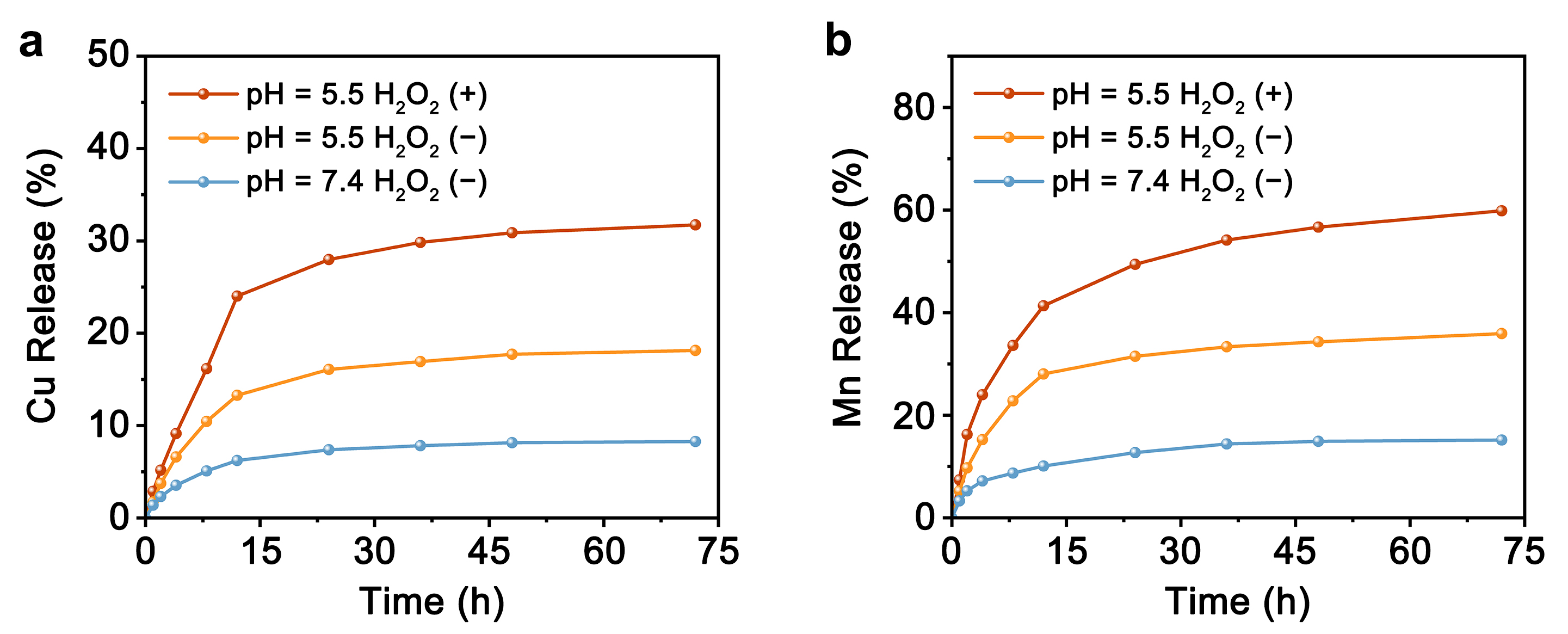


**Fig. S28** The accumulated concentrations of released **a** Cu and **b** Mn under neutral (pH = 7.4) and acidic (pH = 5.5) conditions with or without H_2_O_2_ (0.1 mM) addition

**
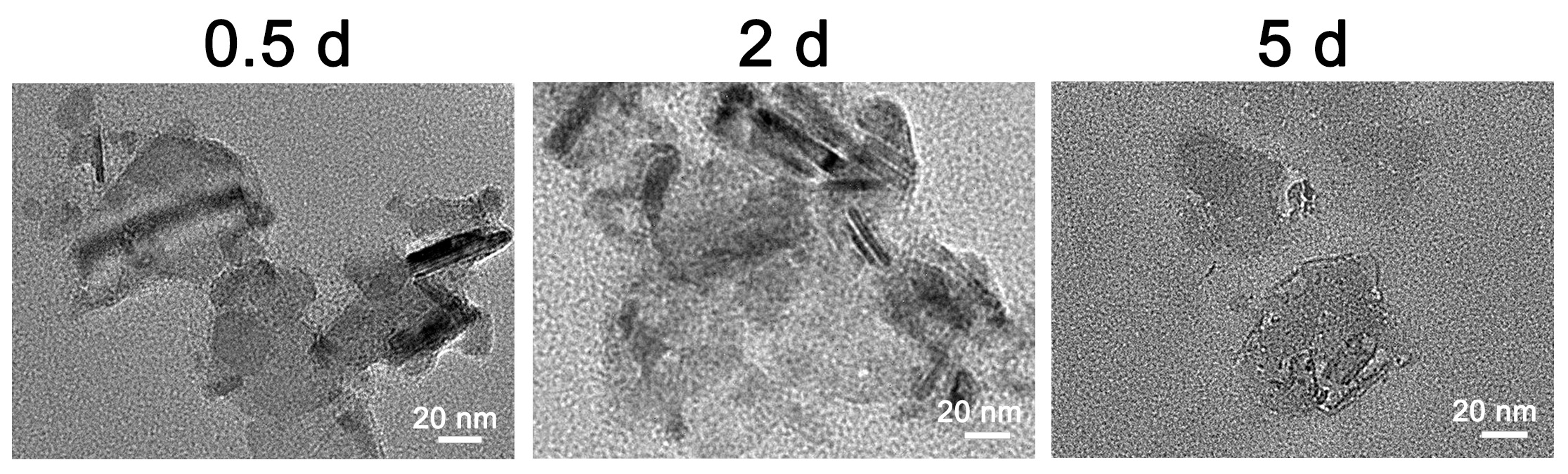
**

**Fig. S29** TEM images of MCPG in acidic condition (pH = 5.5) for various treatment periods

**
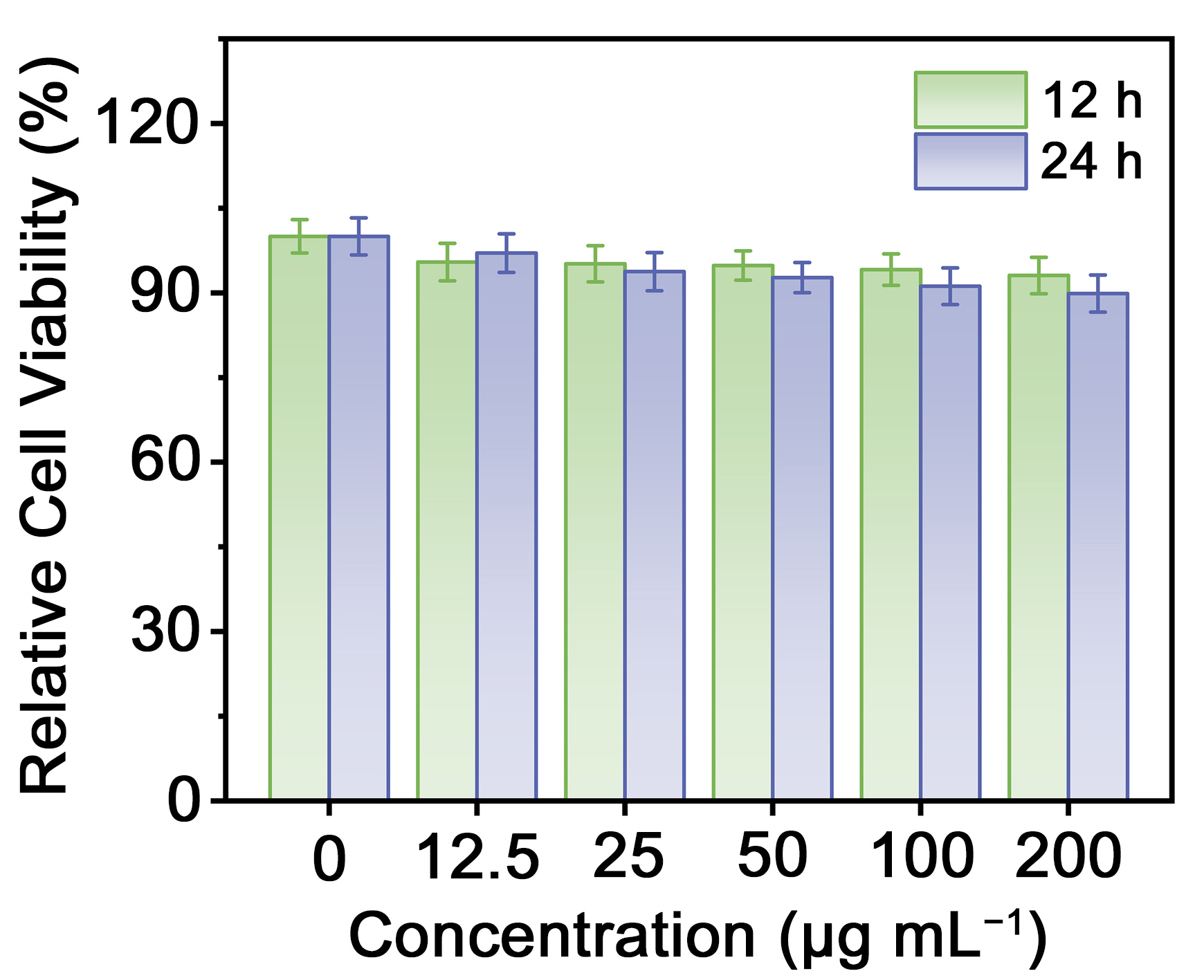
**

**Fig. S30** Cell viability of L929 cells cultivated with various concentrations of MCPG


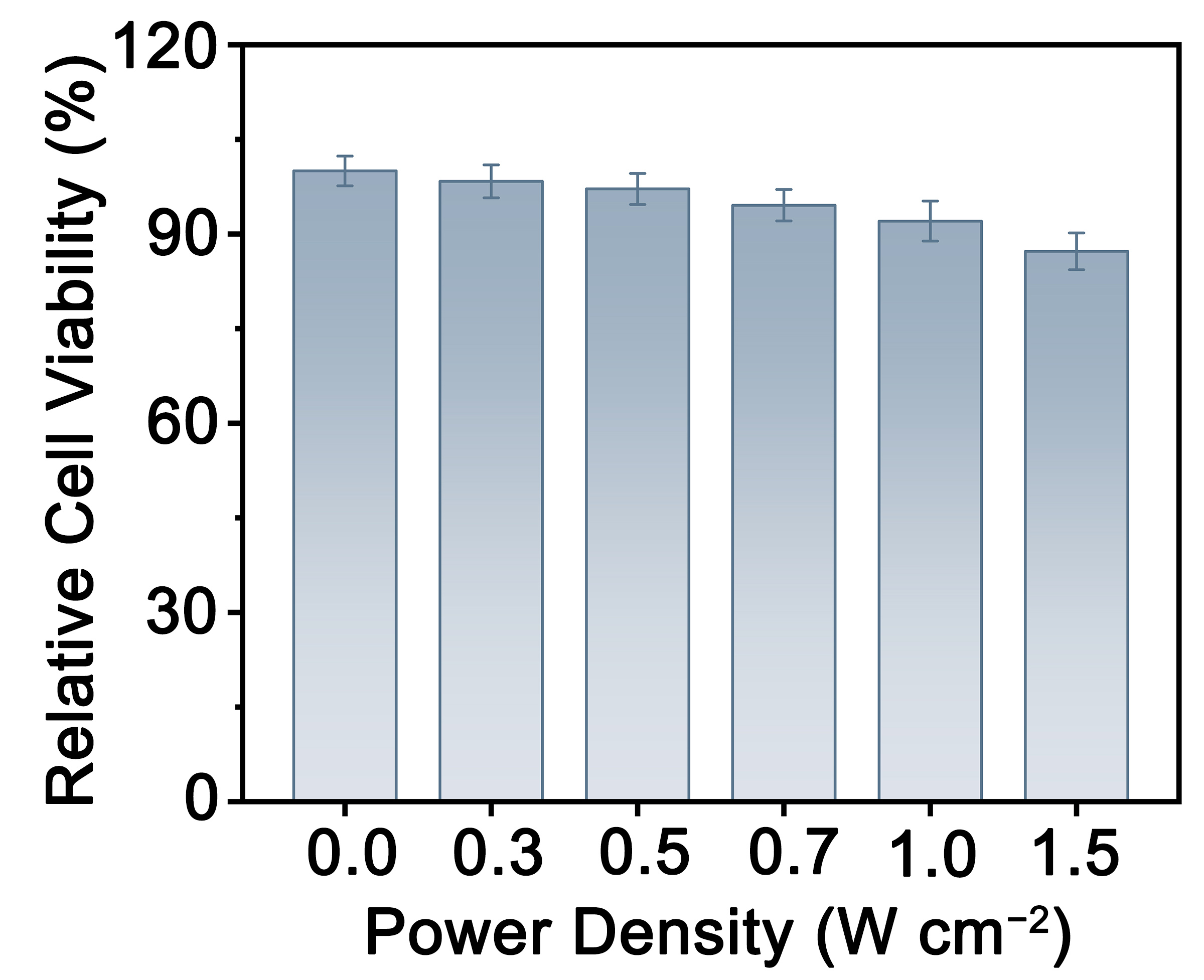


**Fig. S31** Cell viability of L929 cells after irradiated with various laser power densities


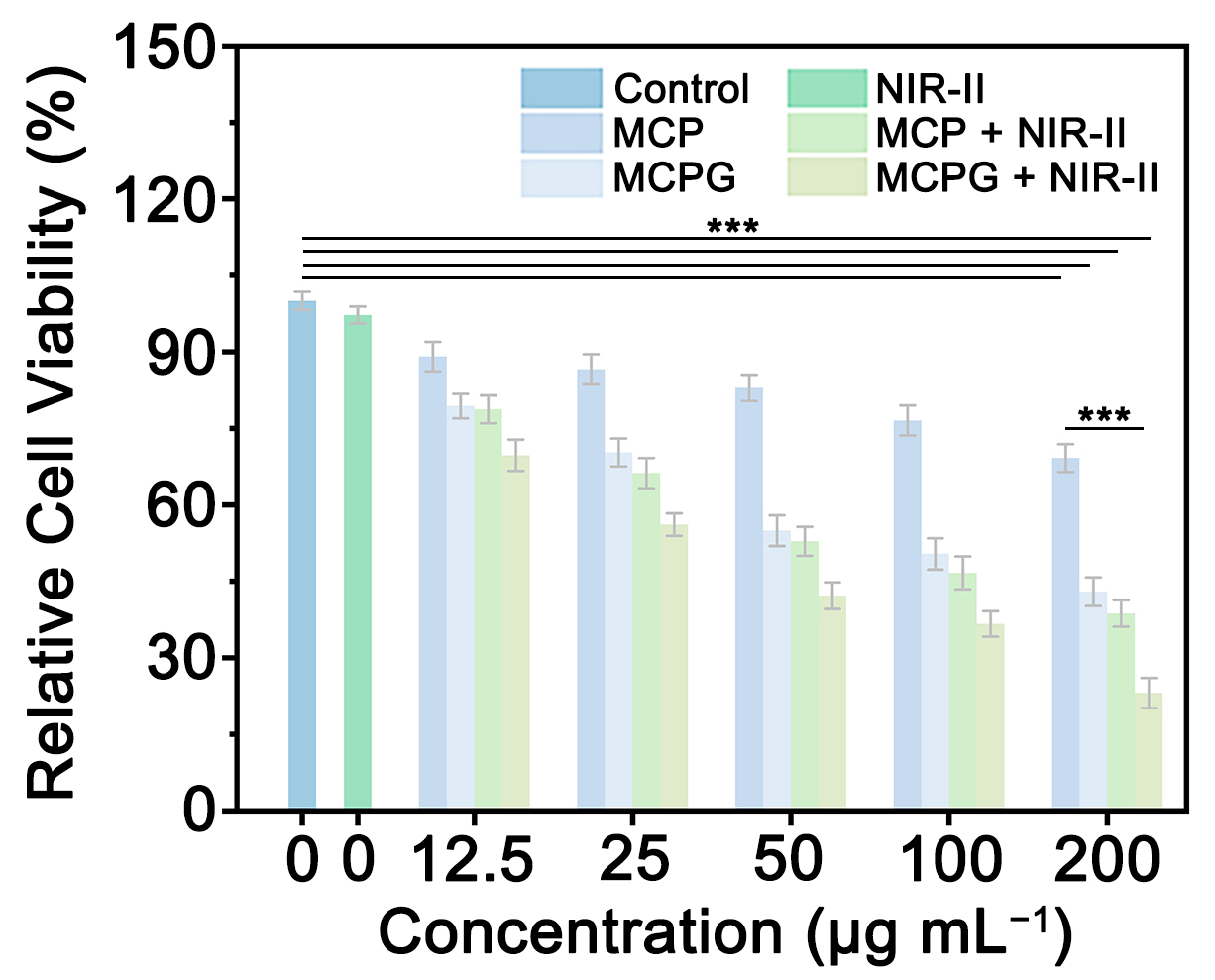


**Fig. S32** Cell viability of HepG2 hepatic carcinoma cells after different treatments


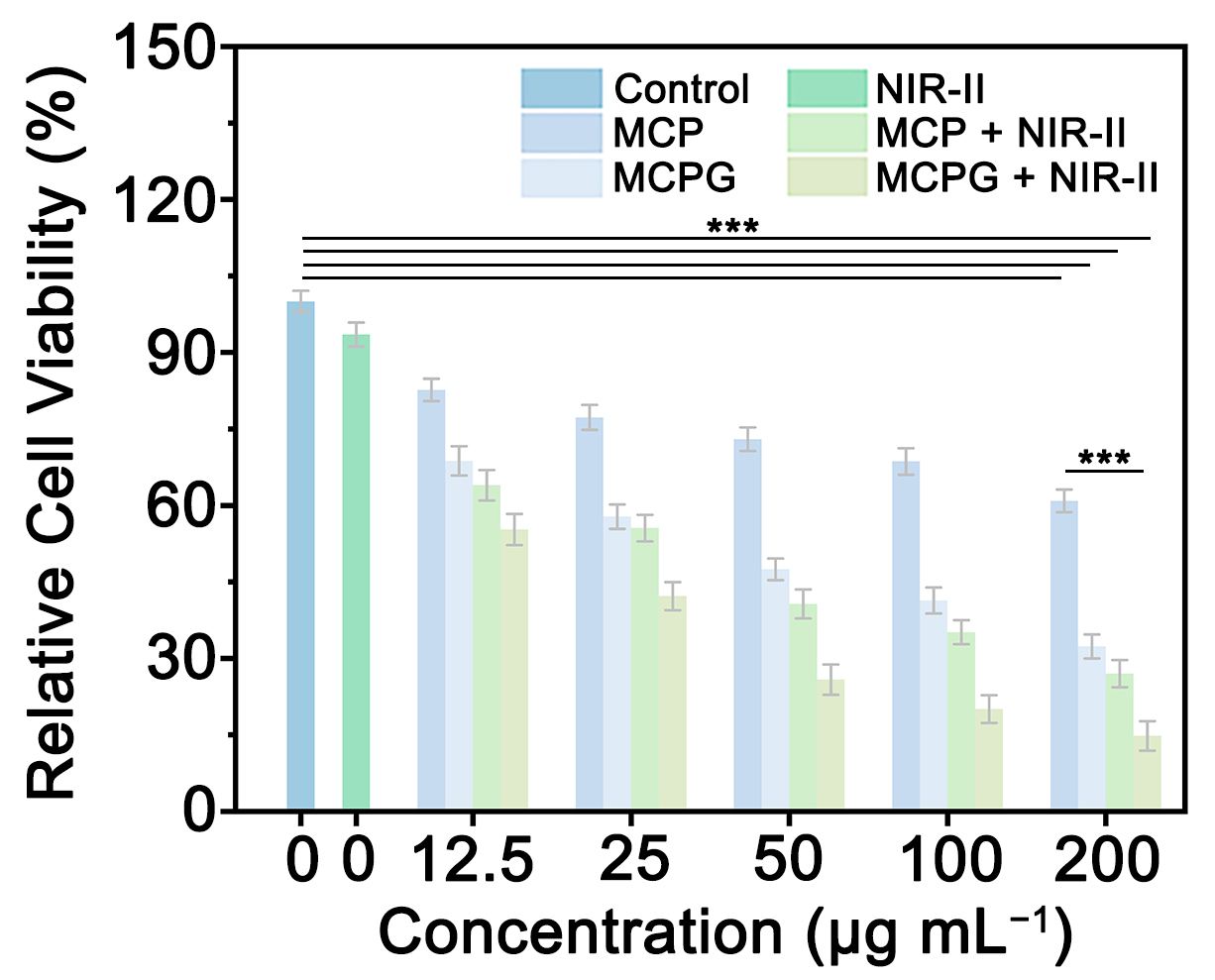


**Fig. S33** Cell viability of A549 lung carcinoma cells after different treatments


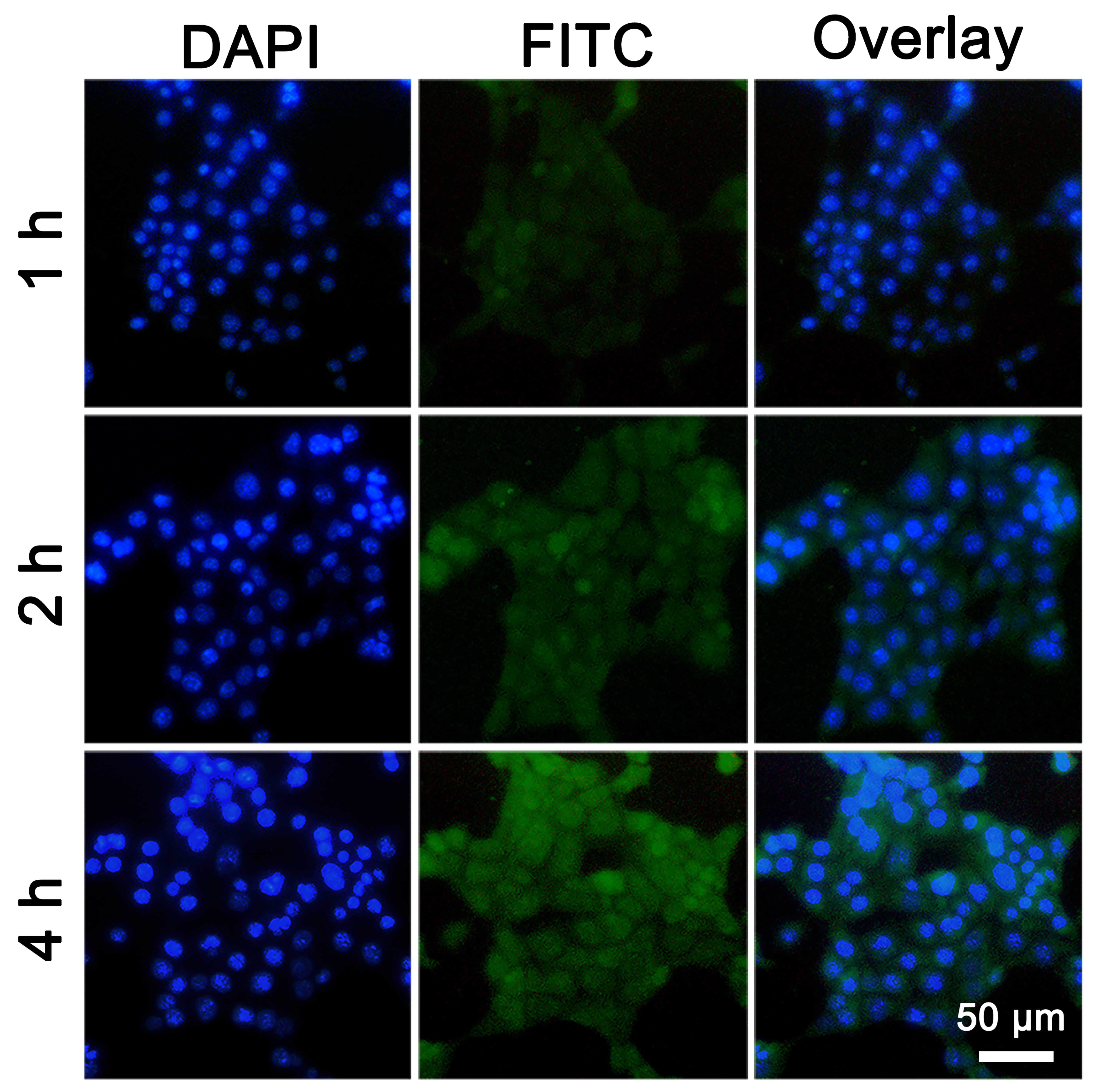


**Fig. S34** Cellular uptake of FITC-modified MCPG by 4T1 cells at different time points


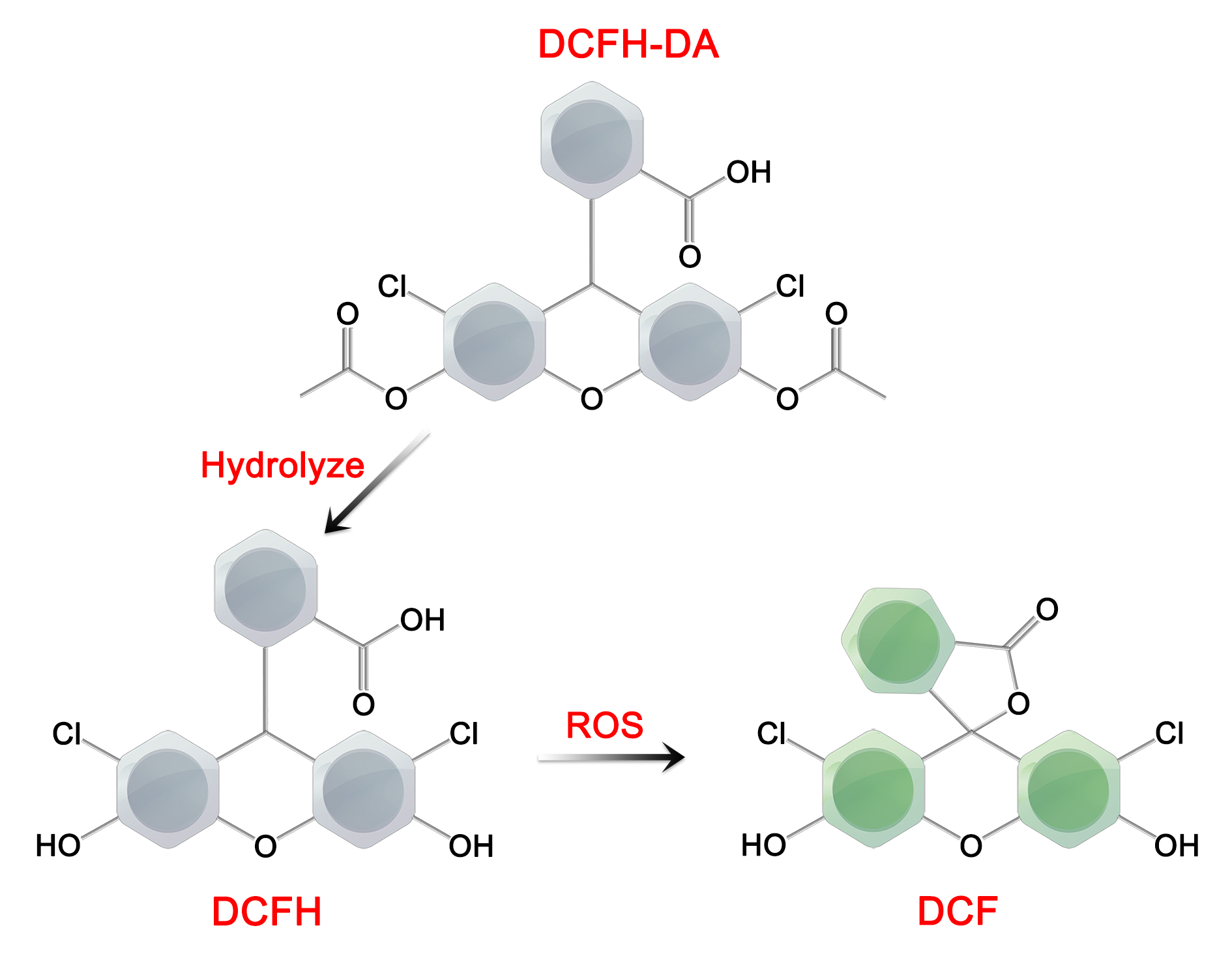


**Fig. S35** Schematic illustration of the chromogenic principle of DCFH-DA


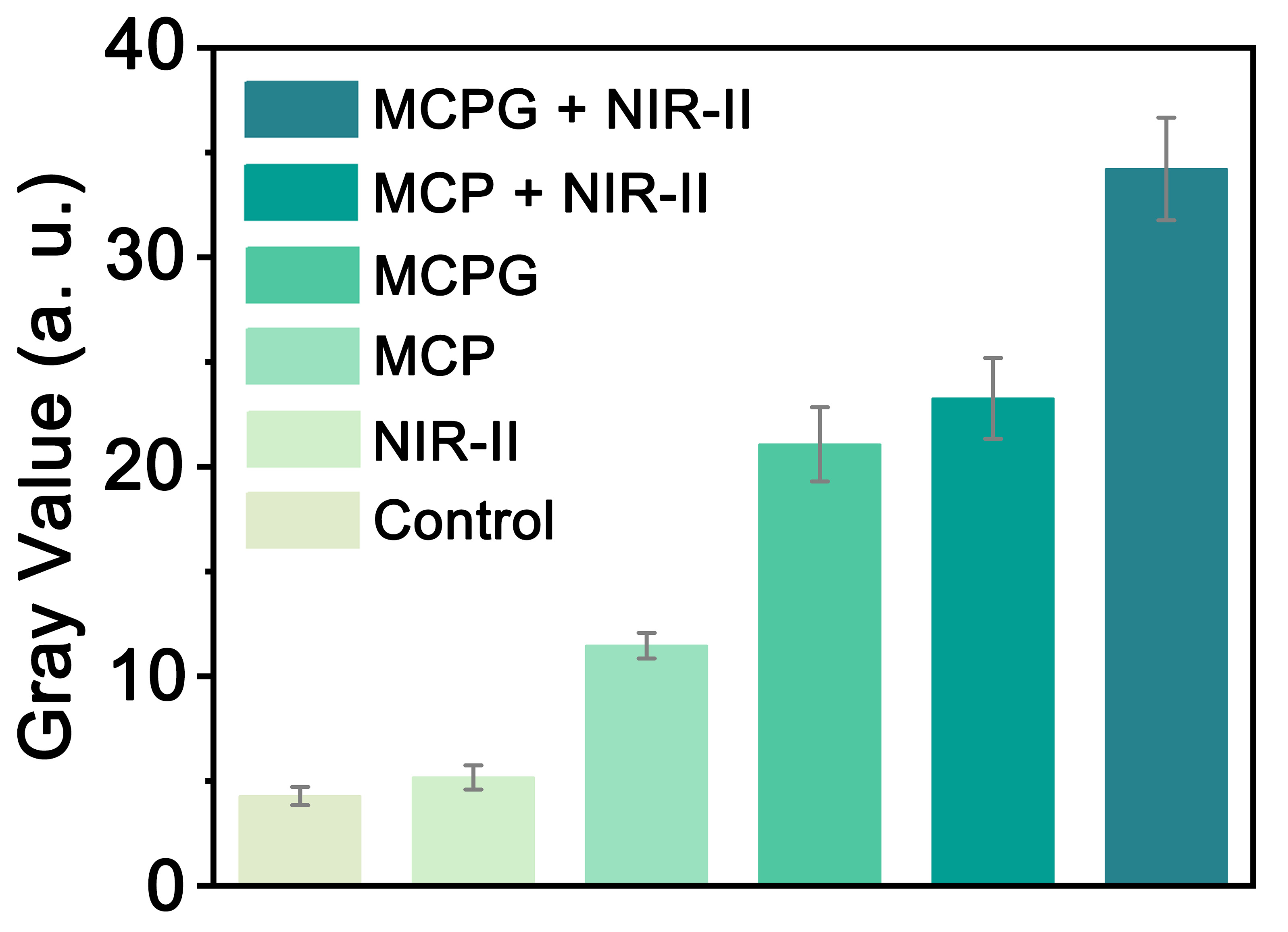


**Fig. S36** Semi-quantitative analysis of DCFH-DA staining of 4T1 cells with various treatments


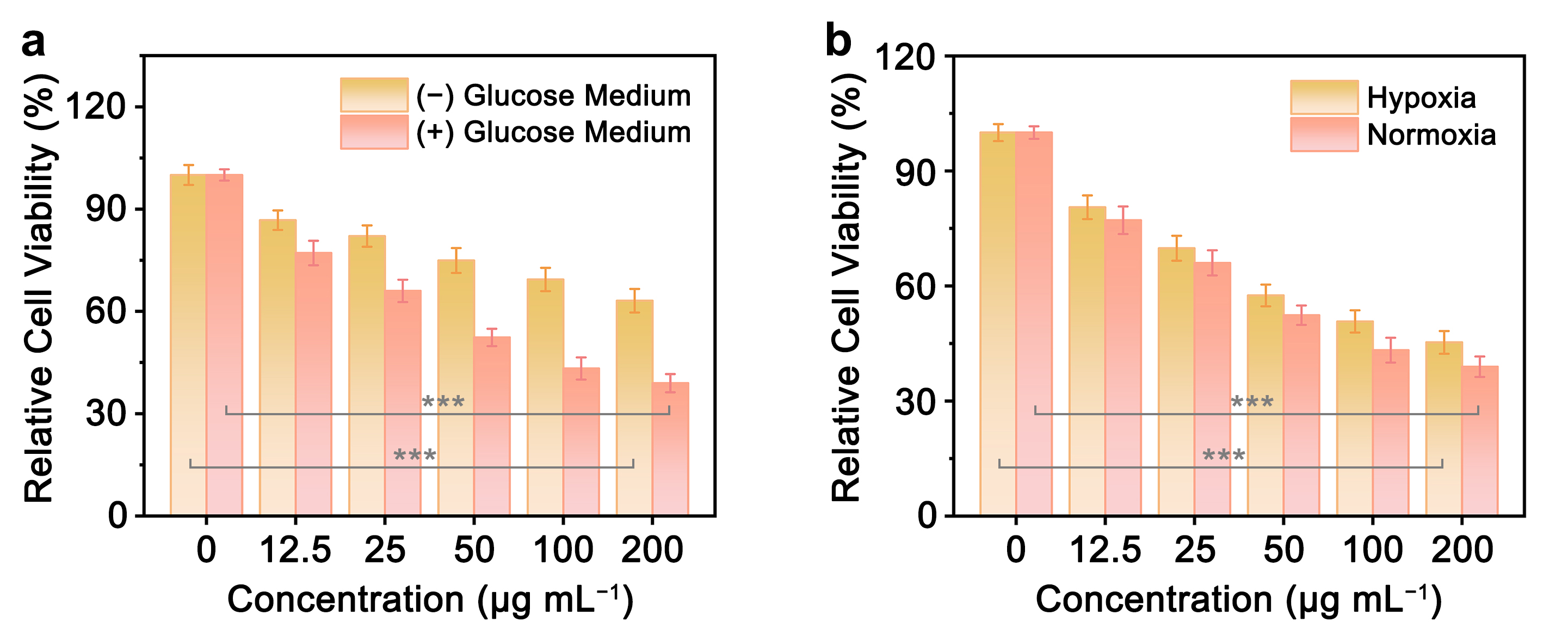


**Fig. S37 a** Cell viability of 4T1 cells incubated with MCPG in medium with or without glucose addition. **b** Cell viability of 4T1 cells incubated with MCPG in hypoxia and normoxia conditions


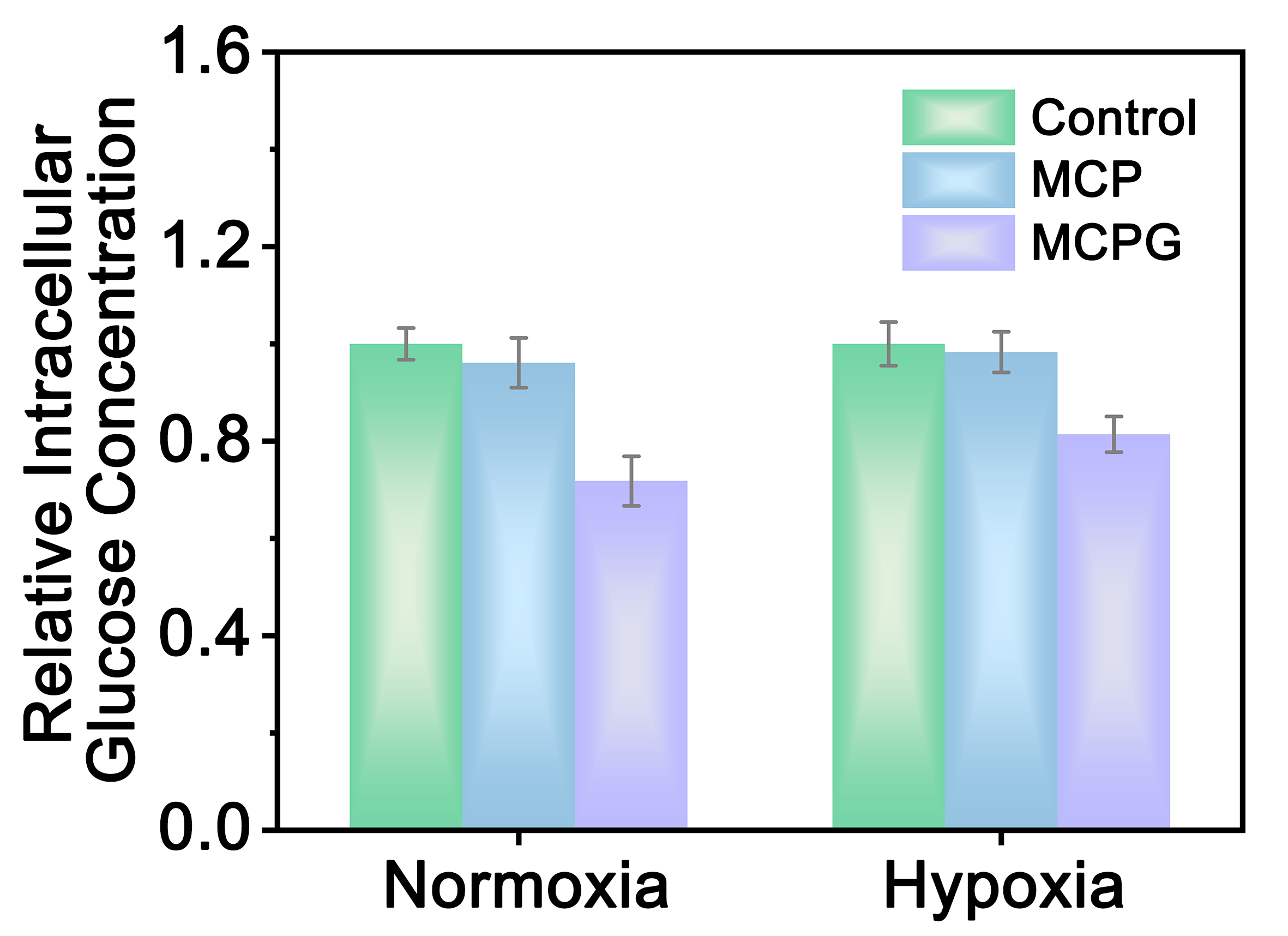


**Fig. S38** The relatively intracellular glucose concentration of 4T1 cells cultivated with different formulations in hypoxia and normoxia conditions


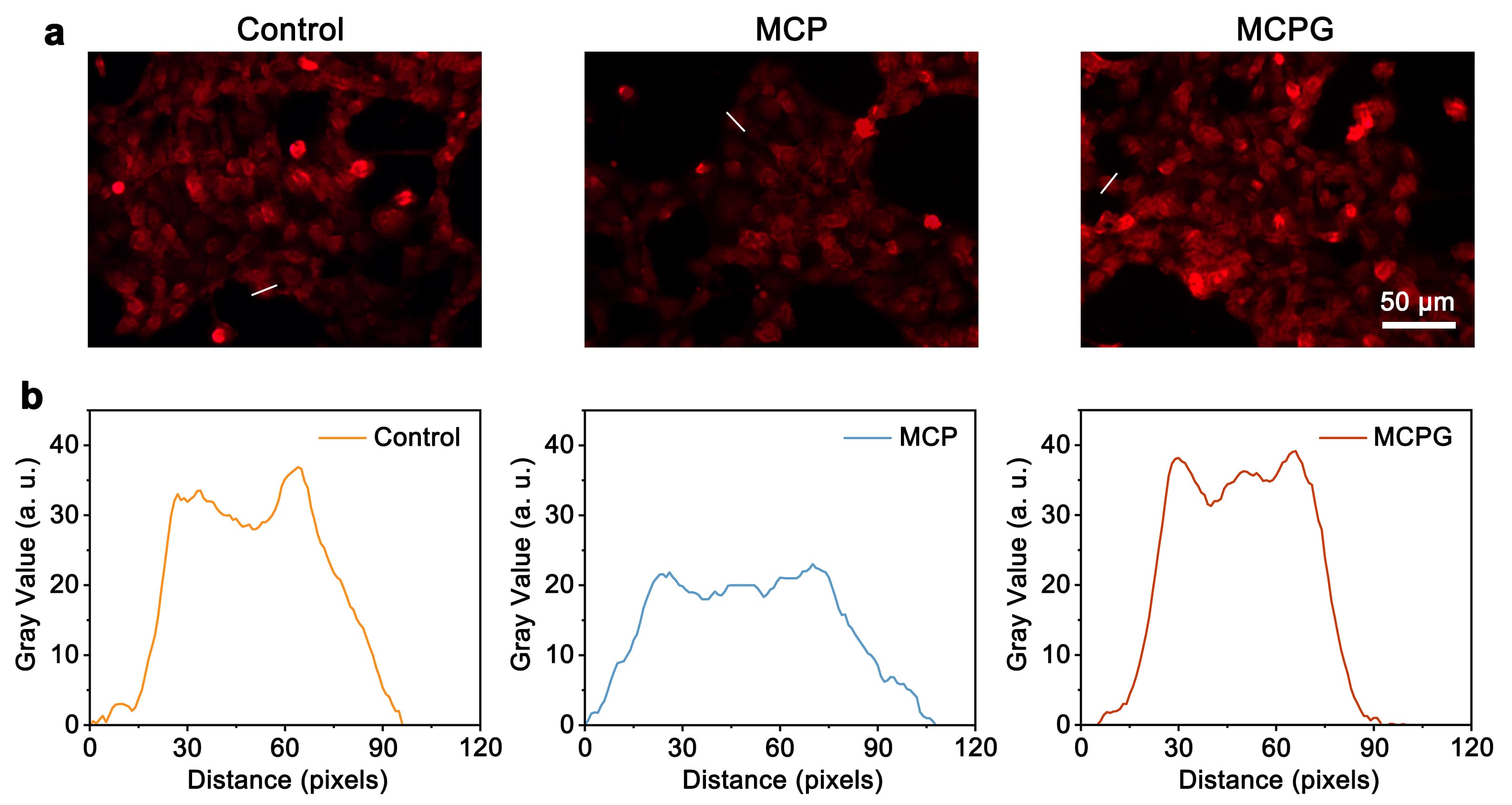


**Fig. S39 a** Fluorescence images of intracellular O_2_ generation after different treatments using [Ru(dpp)_3_]Cl_2_ as a probe and **b** the line-scan profiles


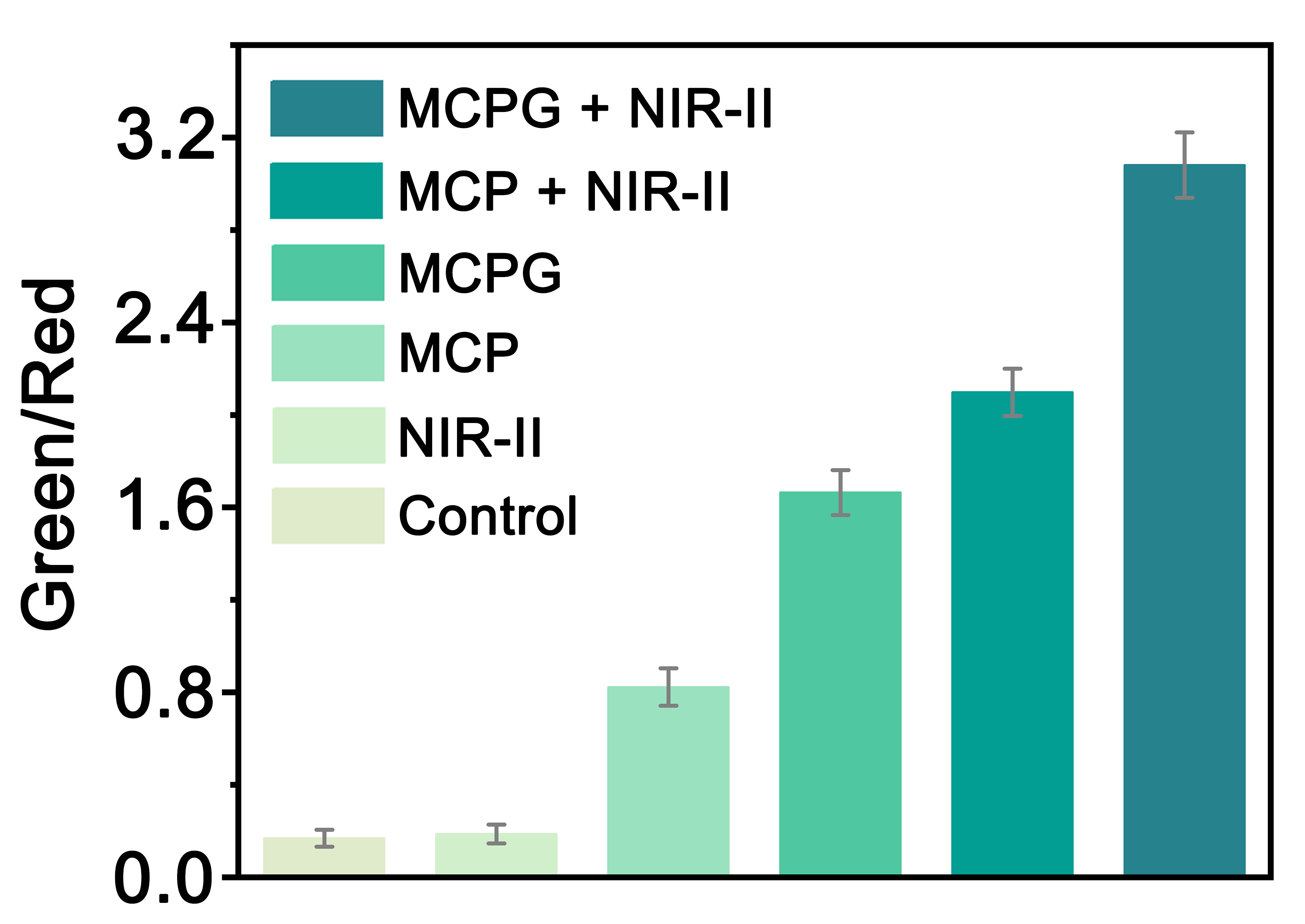


**Fig. S40** Semi-quantitative analysis of JC-1 staining of 4T1 cells with different treatments


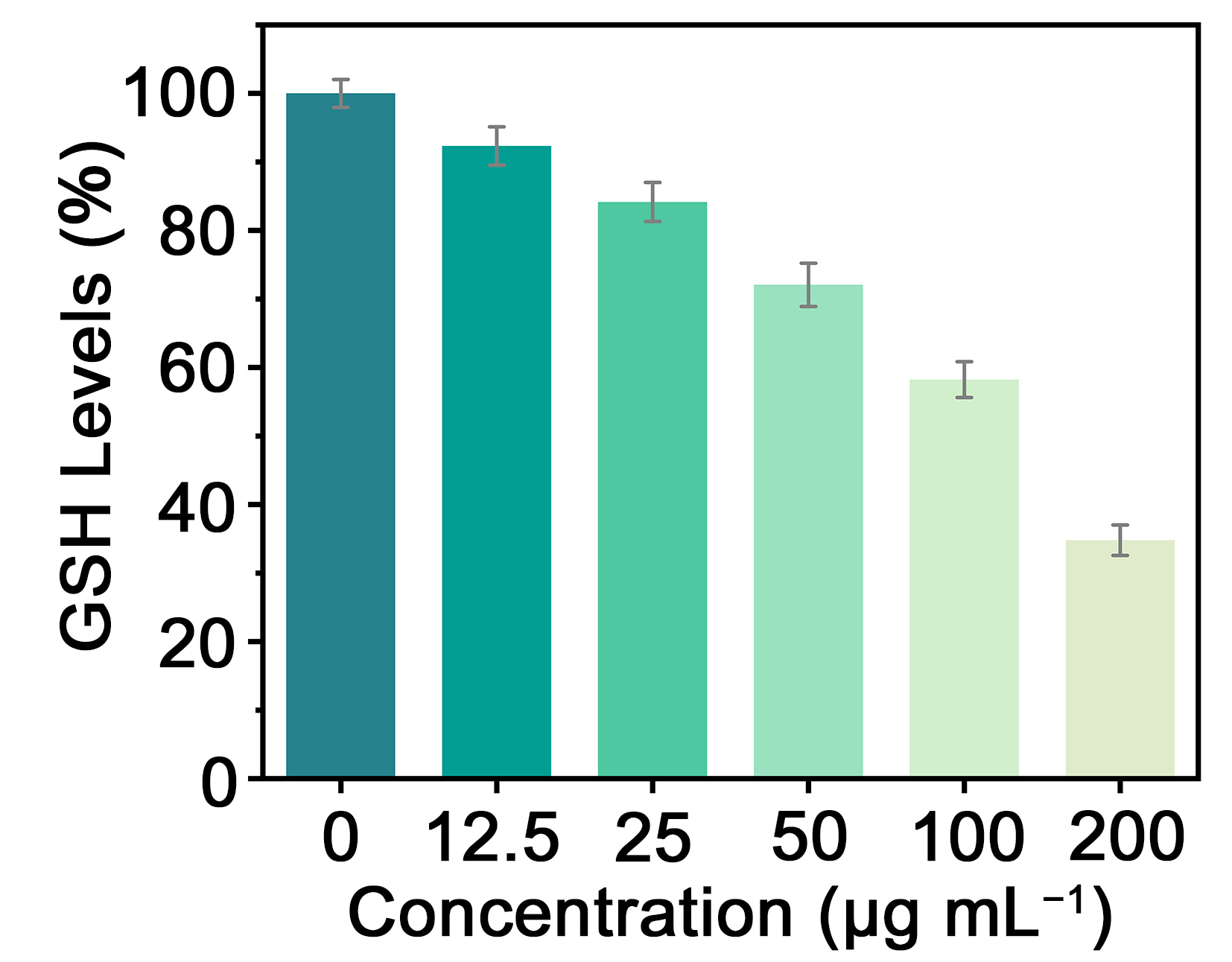


**Fig. S41** GSH levels in 4T1 cells incubated with various concentrations of MCPG


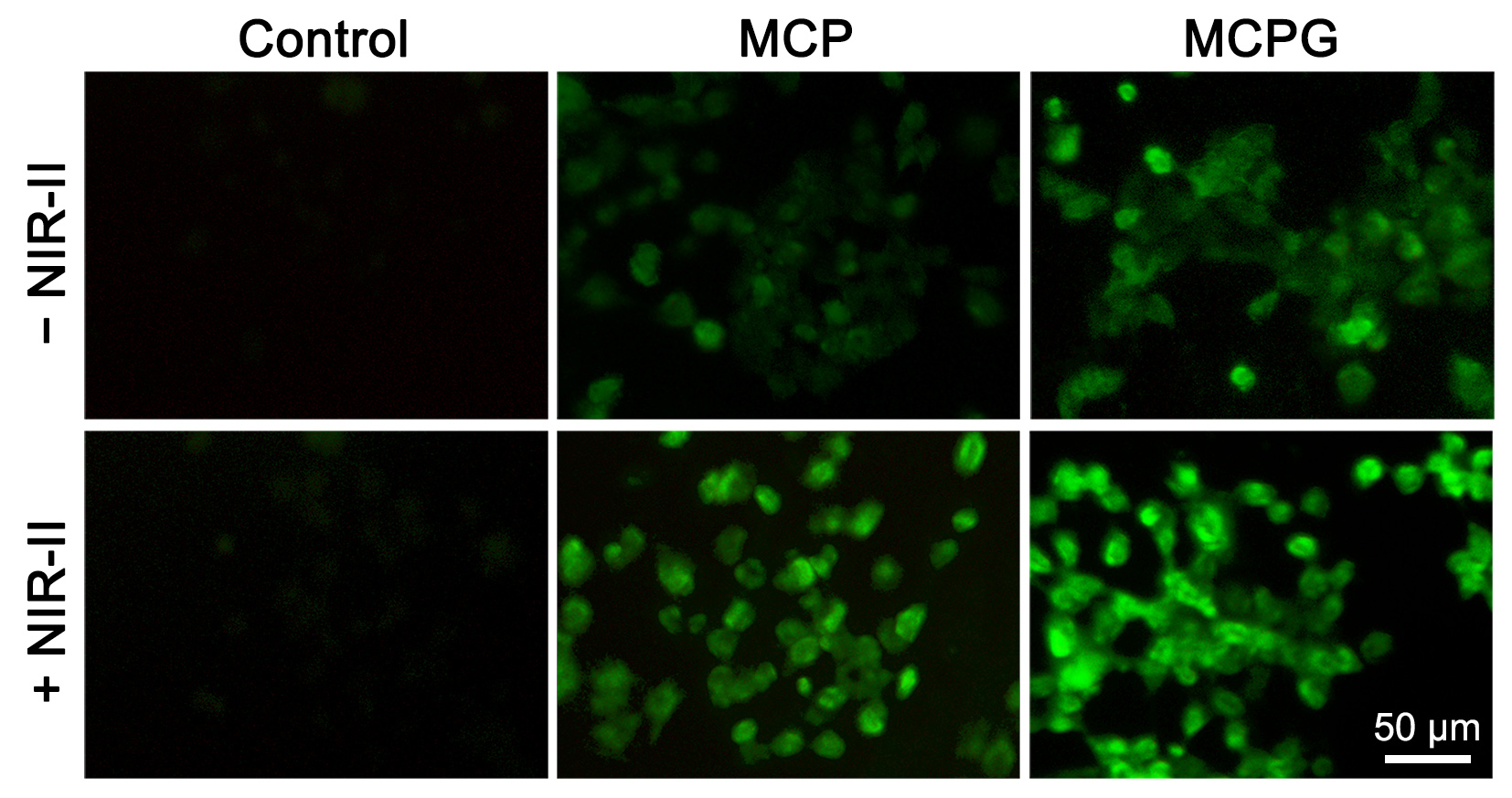


**Fig. S42** Liperfluo staining images of 4T1 cells after various treatments


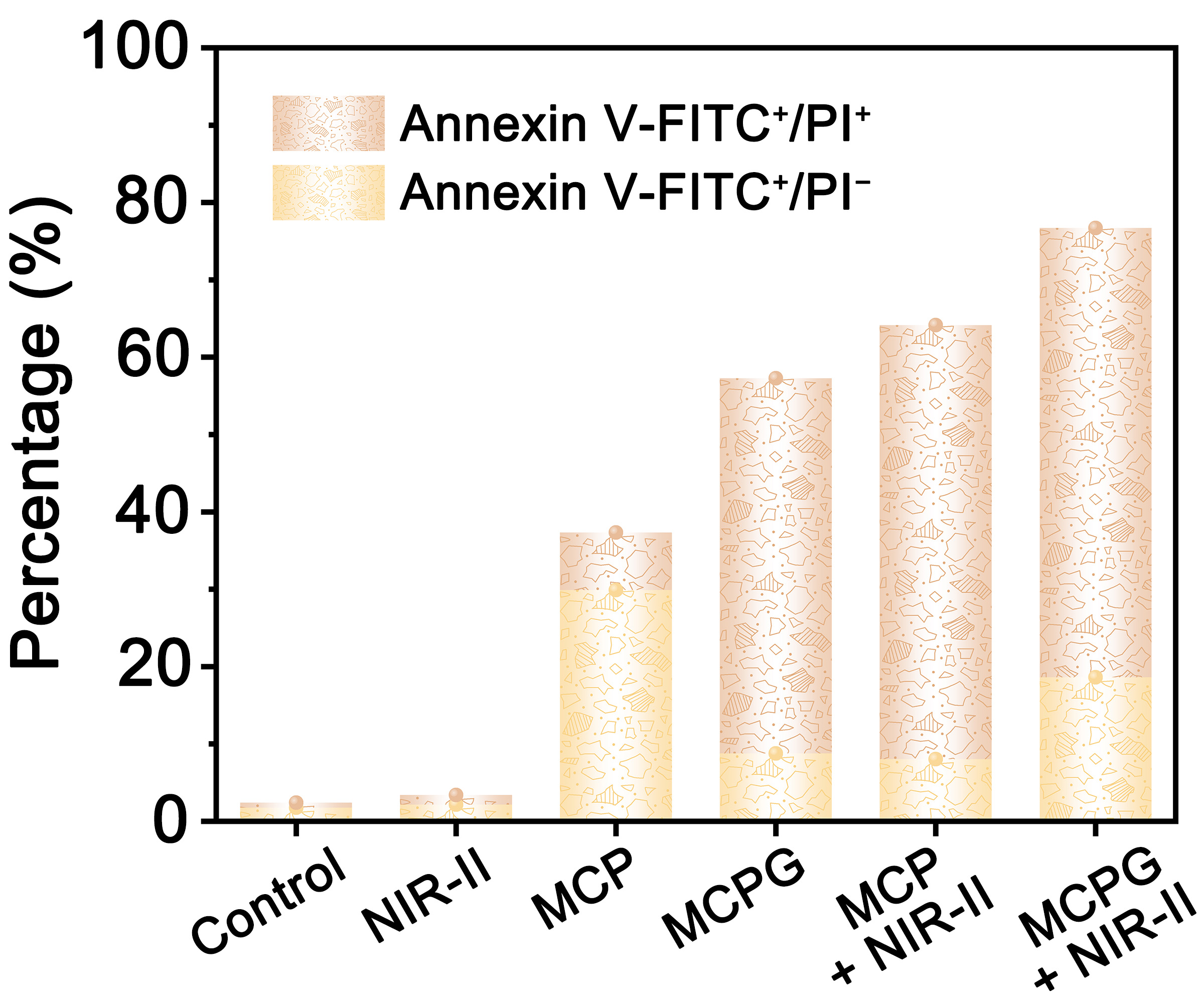


**Fig. S43** The quantitative analysis of 4T1 cells treated with different formulations by flow cytometry


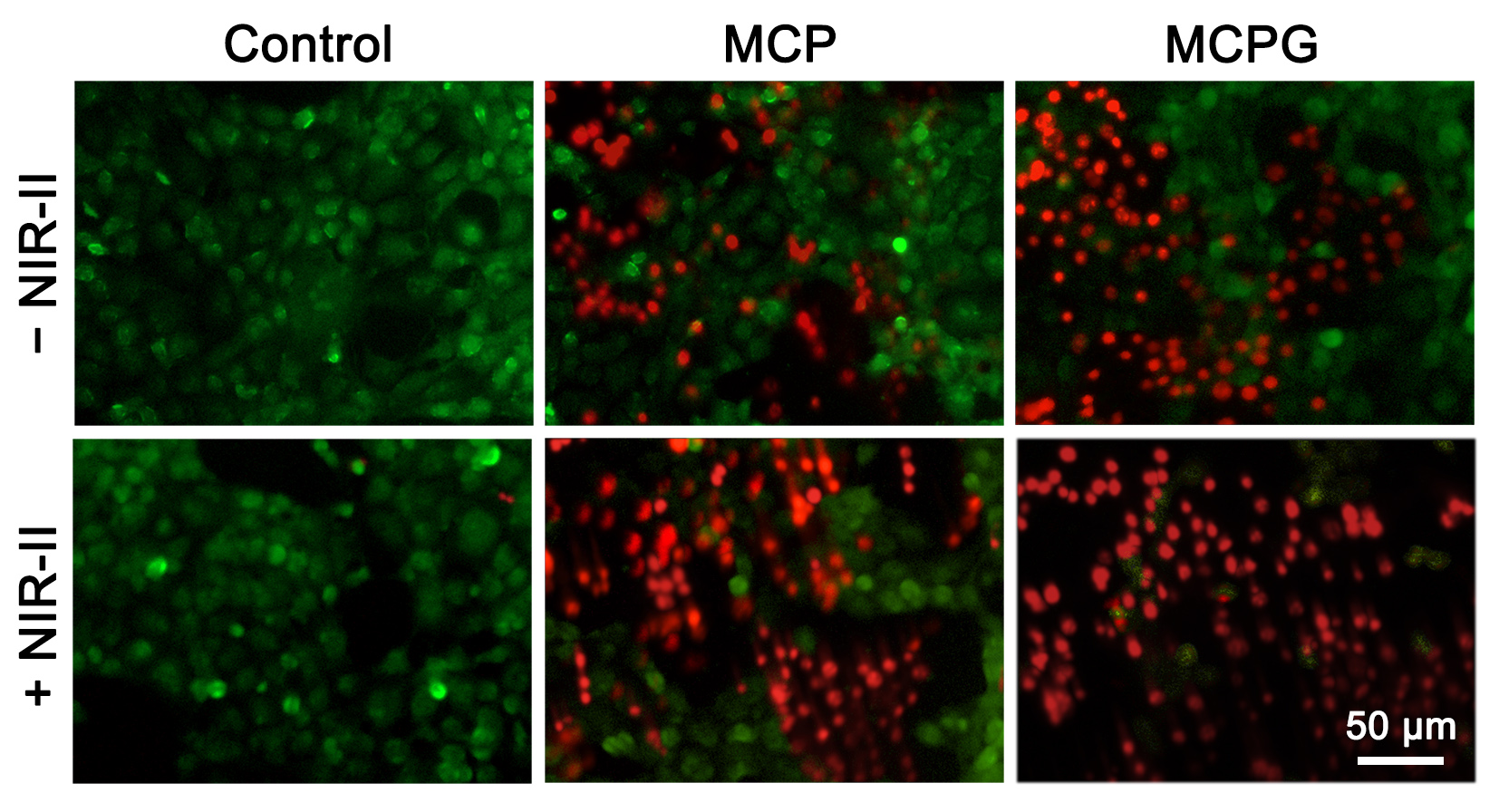


**Fig. S44** Calcein AM/PI co-staining images of 4T1 cells treated with different conditions


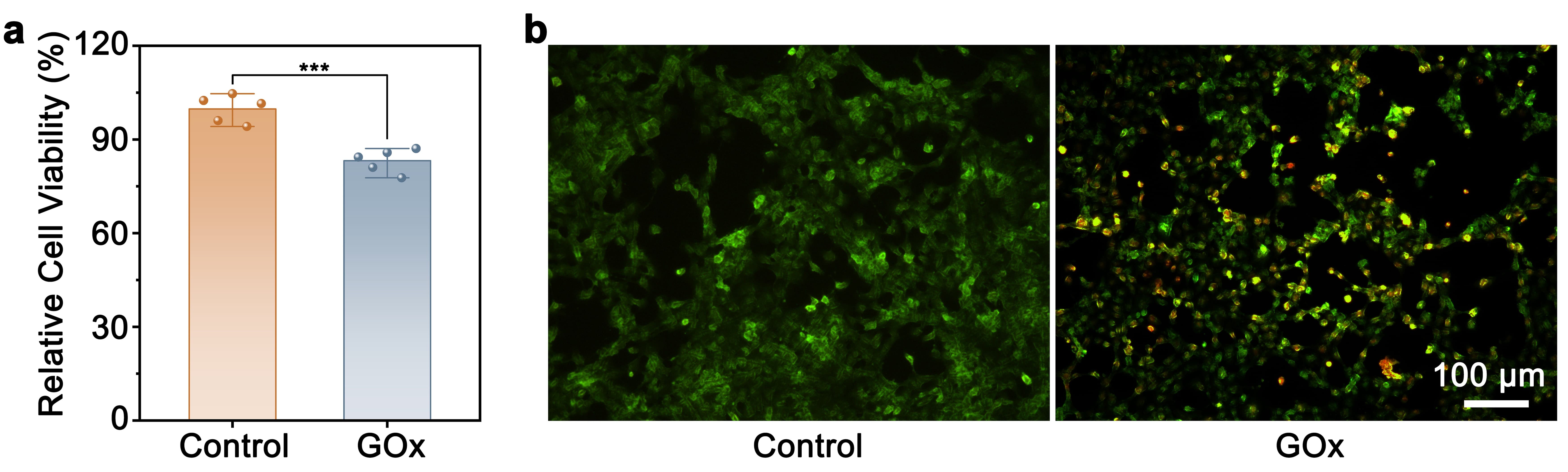


**Fig. S45 a** Cell viability and **b** Calcein AM/PI co-staining images of the control and GOx treatment groups


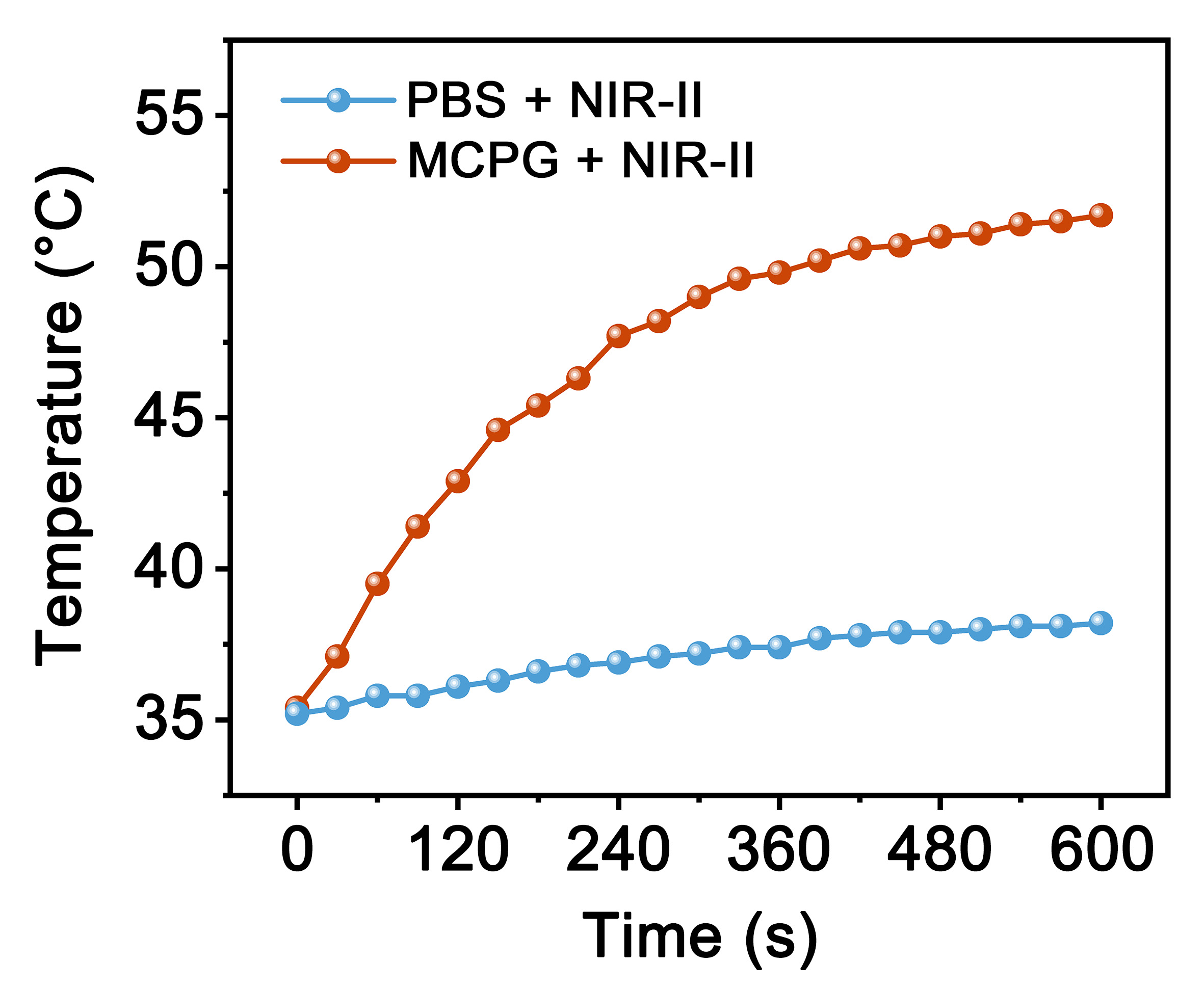


**Fig. S46** Temperature change curves of tumor site after the mice injected with PBS and MCPG under 1064 nm laser irradiation for different times


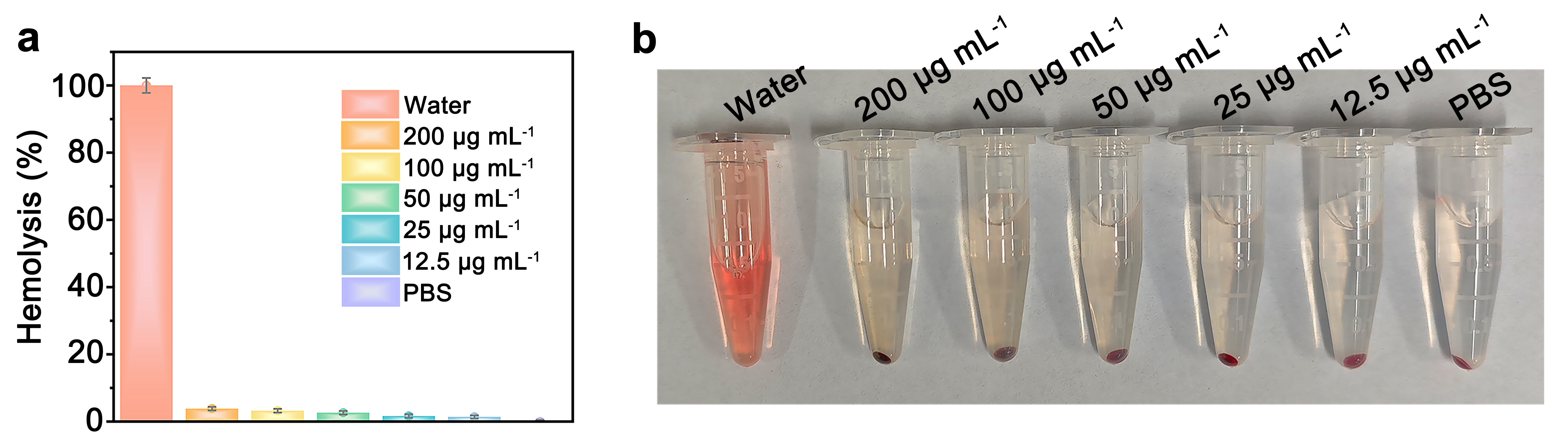


**Fig. S47 a** Hemolysis analysis and **b** the digital photograph after incubation of blood with water (positive control), PBS (negative control), and MCPG at varying concentrations


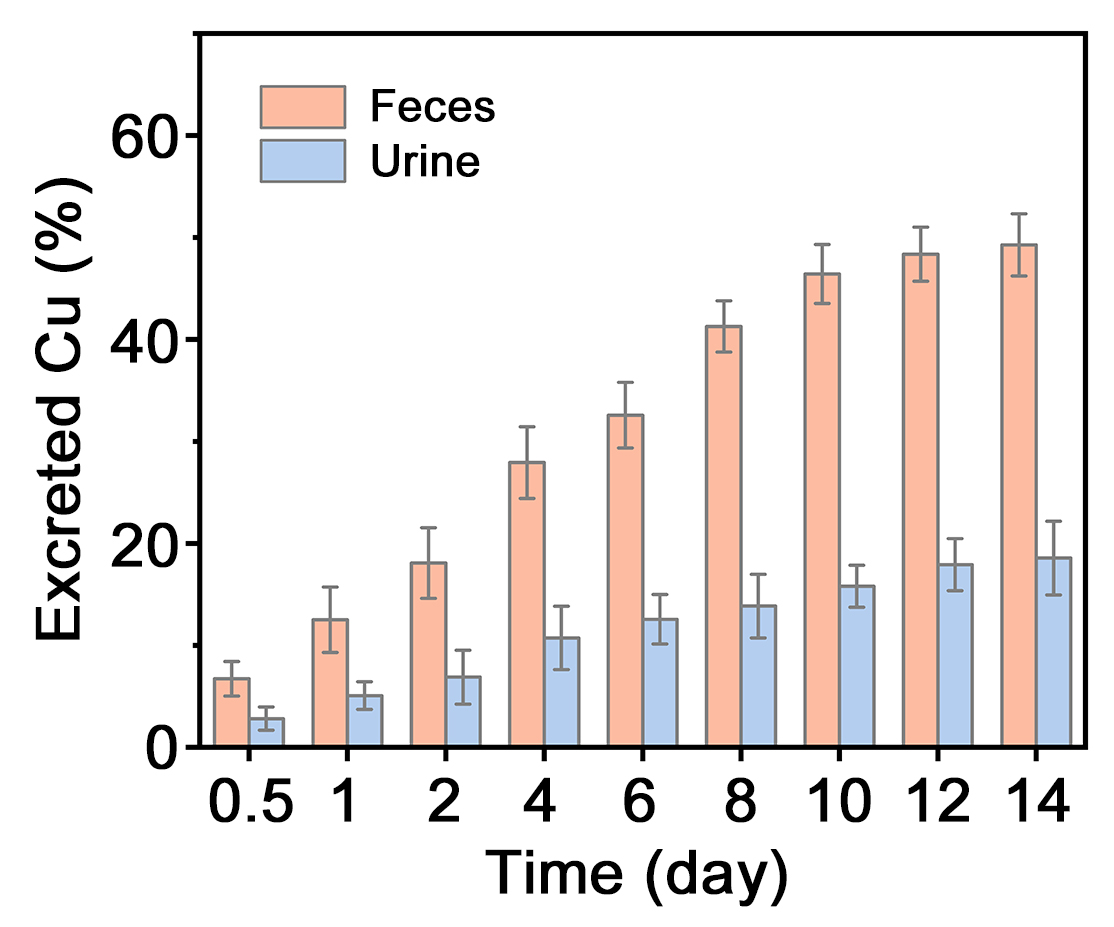


**Fig. S48** The excreted Cu ion in feces and urine at different treatment time points


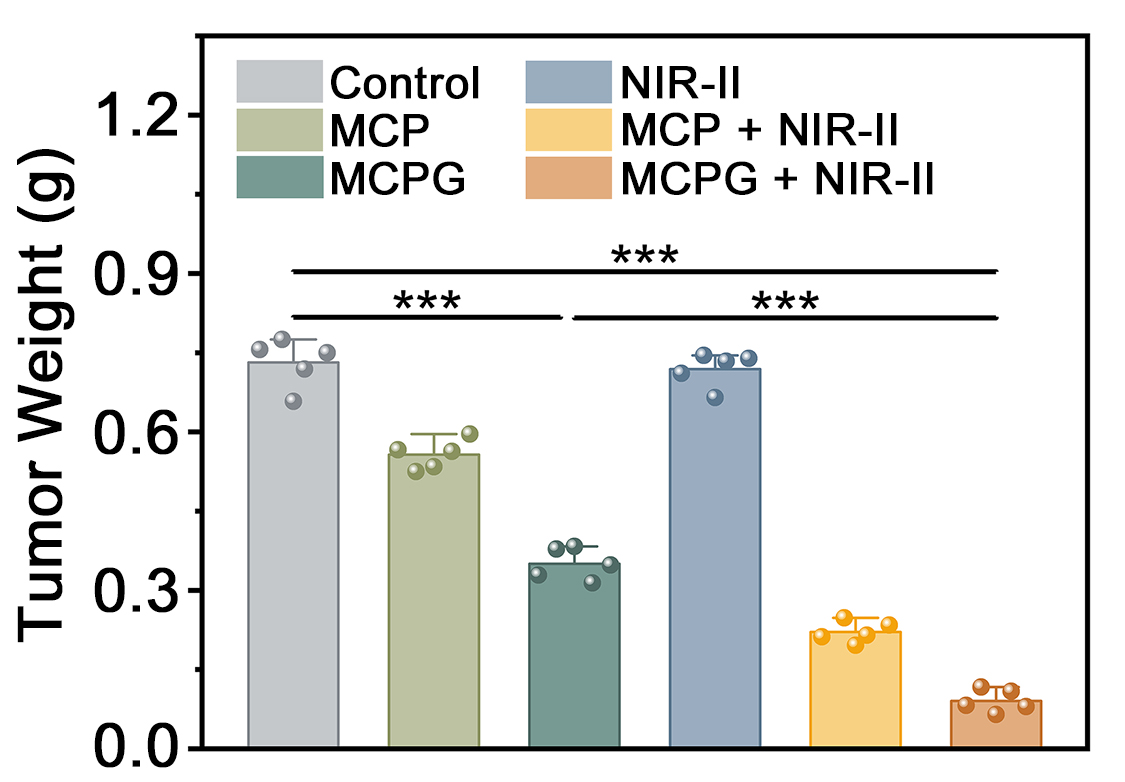


**Fig. S49** The average tumor weight of representative mice in varying treatment groups


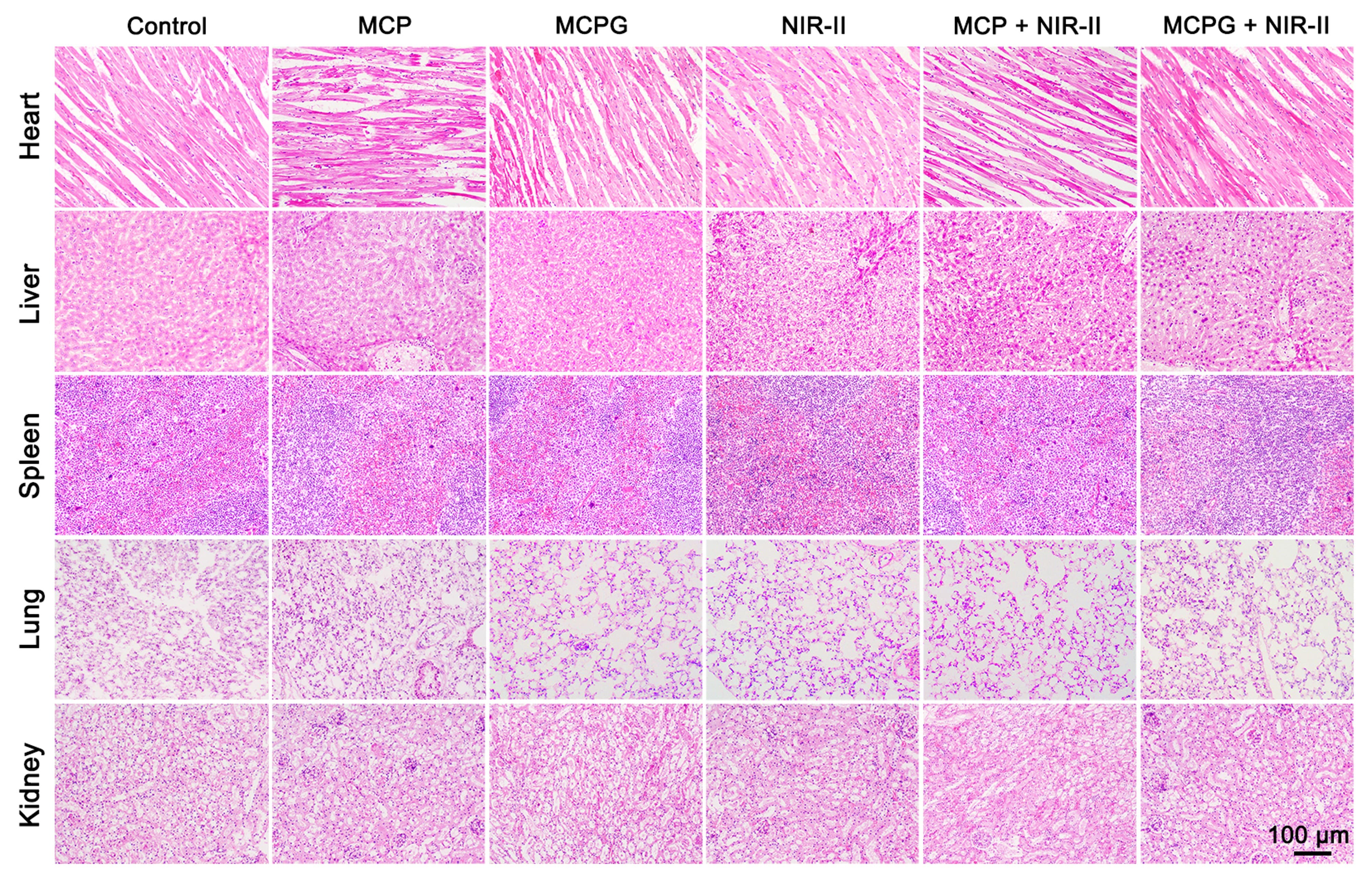


**Fig. S50** H&E staining images of heart, liver, spleen, lung, and kidney obtained from the representative mice in various groups after 14 days of treatment


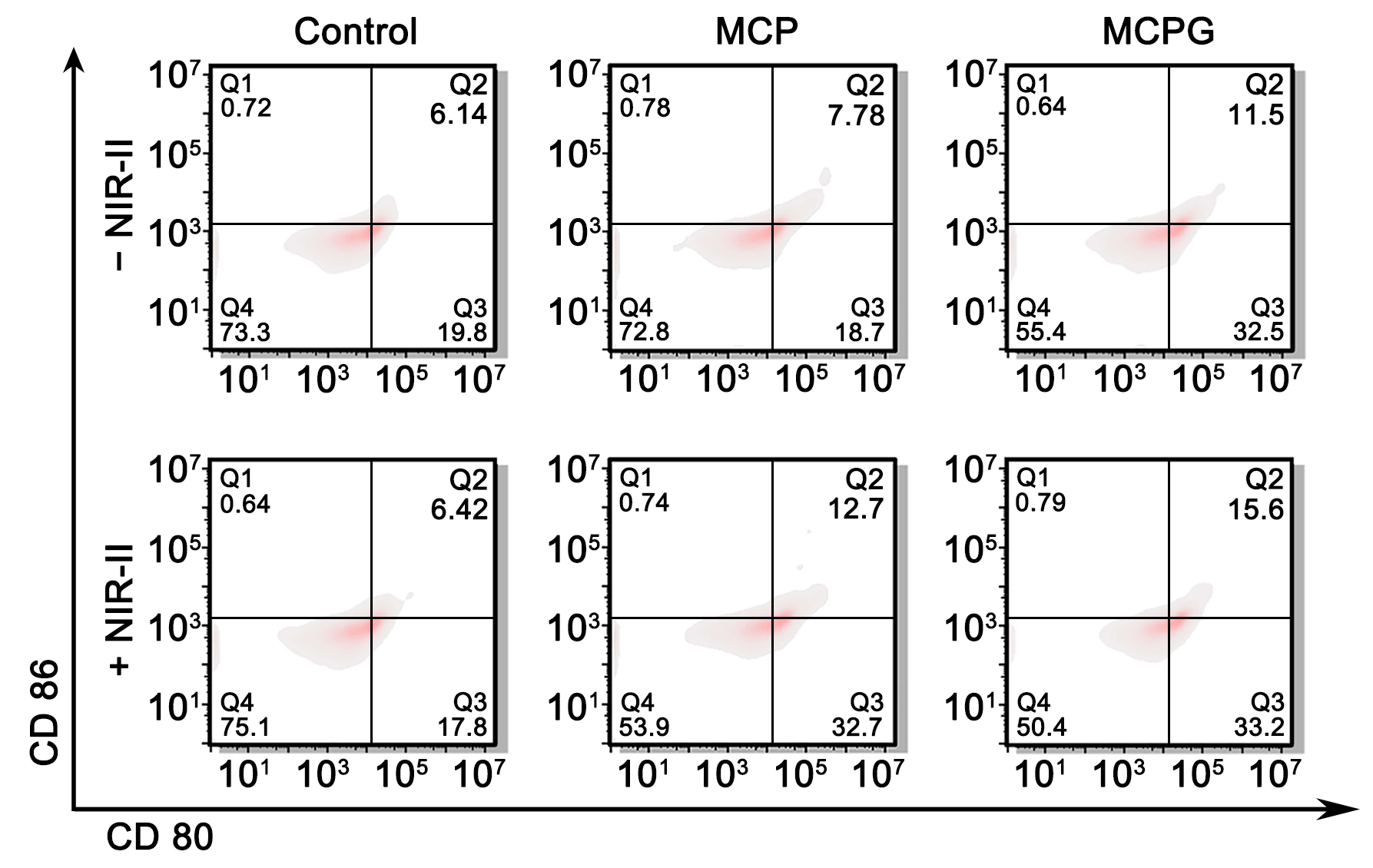


**Fig. S51** Flow cytometry assay of CD80 and CD86 after varying treatments

**Supplementary References**

1. L. Wang, M. Huo, Y. Chen, J. Shi, Iron-engineered mesoporous silica nanocatalyst with biodegradable and catalytic framework for tumor-specific therapy. Biomaterials **163**, 1–13 (2018). <https://doi.org/10.1016/j.biomaterials.2018.02.018>
2. L. Feng, S. Gai, F. He, P. Yang, Y. Zhao, Multifunctional bismuth ferrite nanocatalysts with optical and magnetic functions for ultrasound-enhanced tumor theranostics. ACS Nano **14**(6), 7245–7258 (2020). <https://doi.org/10.1021/acsnano.0c02458>
3. S. Sun, X. Liu, X. Meng, Z. Yang, X. Zhang et al., Bimetallic metal-organic framework microneedle array for wound healing through targeted reactive oxygen species generation and electron transfer disruption. ACS Nano **19**(15), 15109–15119 (2025). <https://doi.org/10.1021/acsnano.5c02923>
4. Y. Cheng, Y.-D. Xia, Y.-Q. Sun, Y. Wang, X.-B. Yin, “Three-in-one” nanozyme composite for augmented cascade catalytic tumor therapy. Adv. Mater. **36**(8), e2308033 (2024). <https://doi.org/10.1002/adma.202308033>
5. Y. Du, X. Zhao, F. He, H. Gong, J. Yang et al., A vacancy-engineering ferroelectric nanomedicine for cuproptosis/apoptosis co-activated immunotherapy. Adv. Mater. **36**(30), 2403253 (2024). <https://doi.org/10.1002/adma.202403253>
6. L. Fang, Y. Zhang, H. Ding, S. Liu, J. Wei et al., PdCu_x_ bimetallic nanoalloys with "hand-in-hand" collaboration in pod-like activity and "back-to-back" confrontation in SPR effect for tumor redox system control. Adv. Funct. Mater. **34**, 13 (2024). <https://doi.org/10.1002/adfm.202309338>
7. S. Dong, Y. Dong, B. Liu, J. Liu, S. Liu et al., Guiding transition metal-doped hollow cerium tandem nanozymes with elaborately regulated multi-enzymatic activities for intensive chemodynamic therapy. Adv. Mater. **34**(7), 2107054 (2022). <https://doi.org/10.1002/adma.202107054>
8. S. Dong, Y. Dong, T. Jia, S. Liu, J. Liu et al., GSH-depleted nanozymes with hyperthermia-enhanced dual enzyme-mimic activities for tumor nanocatalytic therapy. Adv. Mater. **32**(42), 2002439 (2020). <https://doi.org/10.1002/adma.202002439>
9. B. Shao, Y. Zhu, Y. Du, D. Yang, S. Gai et al., Mn-doped single atom nanozyme composited Au for enhancing enzymatic and photothermal therapy. J. Colloid Interface Sci. **628**, 419–434 (2022). <https://doi.org/10.1016/j.jcis.2022.08.053>
10. Z. Wang, B. Liu, Q. Sun, L. Feng, F. He et al., Upconverted metal-organic framework Janus architecture for near-infrared and ultrasound co-enhanced high performance tumor therapy. ACS Nano **15**(7), 12342–12357 (2021). <https://doi.org/10.1021/acsnano.1c04280>
